# Influence of the heavy-atom effect on singlet fission: a study of platinum-bridged pentacene dimers†
†Electronic supplementary information (ESI) available: Experimental procedures, synthetic protocols, spectroscopic details, analysis methods for the spectroscopic data, supplemental theory results, supplemental Tables S1–S4, Fig. S1–S30. See DOI: 10.1039/c9sc04410h


**DOI:** 10.1039/c9sc04410h

**Published:** 2019-10-21

**Authors:** Bettina S. Basel, Ryan M. Young, Matthew D. Krzyaniak, Ilias Papadopoulos, Constantin Hetzer, Yueze Gao, Nathan T. La Porte, Brian T. Phelan, Timothy Clark, Rik R. Tykwinski, Michael R. Wasielewski, Dirk M. Guldi

**Affiliations:** a Department of Chemistry and Pharmacy & Interdisciplinary Center for Molecular Materials (ICMM) , Friedrich-Alexander-Universität Erlangen-Nürnberg (FAU) , Egerlandstrasse 3 , 91058 Erlangen , Germany . Email: dirk.guldi@fau.de; b Department of Chemistry and Institute for Sustainability and Energy at Northwestern (ISEN) , Northwestern University , Evanston , IL 60208-3113 , USA . Email: m-wasielewski@northwestern.edu; c Department of Chemistry and Pharmacy & Interdisciplinary Center for Molecular Materials (ICMM) , Friedrich-Alexander-Universität Erlangen-Nürnberg (FAU) , Nikolaus-Fiebiger-Strasse 10 , 91058 Erlangen , Germany; d Department of Chemistry , University of Alberta , Edmonton , Alberta T6G 2G2 , Canada . Email: rik.tykwinski@ualberta.ca; e Department of Chemistry and Pharmacy & Computer-Chemistry-Center (CCC) , Friedrich-Alexander-Universität Erlangen-Nürnberg (FAU) , Nägelsbachstrasse 25 , 91052 Erlangen , Germany . Email: tim.clark@fau.de

## Abstract

The process of singlet fission (SF) produces two triplet excited states (T_1_ + T_1_) from one singlet excited exciton (S_1_) and a molecule in its ground state (S_0_). It, thus, possesses the potential to boost the solar cell efficiency above the thermodynamic Shockley–Queisser limit of 33%. A key intermediate in the SF mechanism is the singlet correlated triplet pair state ^1^(T_1_T_1_). This state is of great relevance, as its formation is spin-allowed and, therefore, very fast and efficient. Three fundamentally different pathways to formation of ^1^(T_1_T_1_) have been documented so far. The factors that influence which mechanism is associated with which chromophore, however, remain largely unknown. In order to harvest both triplet excitons independently, a decorrelation of the correlated triplet pair state to two individual triplets is required. This second step of the SF process implies a change in the total spin quantum number. In the case of a dimer, this is usually only possible if the coupling between the two pentacenes is sufficiently weak. In this study, we present two platinum-bridged pentacene dimers in which the pentacenes are coupled strongly, so that spin-decorrelation yielding (T_1_ + T_1_) was initially expected to be outcompeted by triplet–triplet annihilation (TTA) to the ground state. Both platinum-bridged pentacene dimers undergo quantitative formation of the (T_1_T_1_) state on a picosecond timescale that is unaffected by the internal heavy-atom effect of the platinum. Instead of TTA of (T_1_T_1_) to the ground state, the internal heavy-atom effect allows for ^1^(T_1_T_1_)–^3^(T_1_T_1_) and ^1^(T_1_T_1_)–^5^(T_1_T_1_) mixing and, thus, triggers subsequent TTA to the (T_1_S_0_) state and minor formation of (T_1_ + T_1_). A combination of transient absorption and transient IR spectroscopy is applied to investigate the mechanism of the (T_1_T_1_) formation in both dimers. Using a combination of experiment and quantum chemical calculations, we are able to observe a transition from the CT-mediated to the direct SF mechanism and identify relevant factors that influence the mechanism that dominates SF in pentacene. Moreover, a combination of time-resolved optical and electron paramagnetic resonance spectroscopic data allows us to develop a kinetic model that describes the effect of enhanced spin–orbit couplings on the correlated triplet pair state.

## Abstract 2

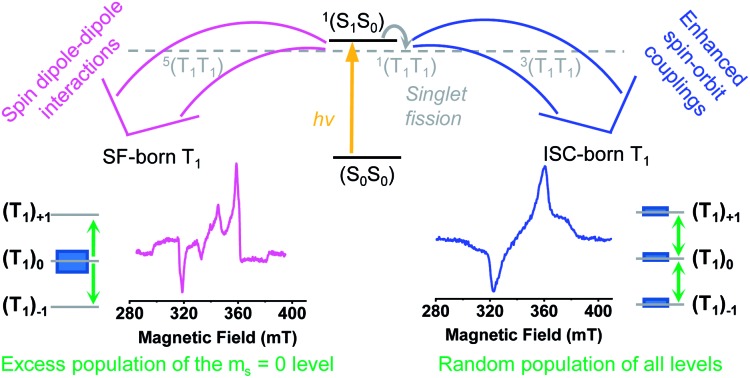
Two platinum-bridged pentacene dimers undergo efficient singlet fission to form a correlated triplet pair (T_1_T_1_). The internal heavy-atom effect of the platinum allows for ^1^(T_1_T_1_)–^3^(T_1_T_1_) mixing leading to the formation of mainly (T_1_S_0_).

## Introduction

Singlet fission (SF) is a fundamental photophysical process that describes the spin conserved reaction of an singlet excited state (S_1_) with a molecule in its electronic ground state (S_0_) to provide a singlet spin-correlated triplet pair state ^1^(T_1_T_1_) (eqn (1)).^1^

1 S_1_ + S_0_ → (T_1_T_1_)^1^

The singlet states (S_1_) and (S_0_), in the case of two monomers, are typically summarized as (S_1_S_0_) in the case of a covalent dimer. The energetic requirement for SF is that the energy of the singlet states (S_1_ + S_0_) or (S_1_S_0_) should be close to or larger than the energy of the triplet product ^1^(T_1_T_1_).^2^ As the total spin does not change during this first step of the SF process, it is spin-allowed; this is fundamentally different from conventional triplet formation *via* intersystem crossing (ISC). ISC is a radiationless transition from one electronic state to another of different spin multiplicity.^3^ If the coupling between the triplet states is weak enough, the ^1^(T_1_T_1_) state can subsequently undergo spin evolution mixing with the quintet spin-correlated triplet pair state, ^5^(T_1_T_1_), and eventually decorrelation that results in two individual triplet states (T_1_ + T_1_) (eqn (2)).^4^–^6^

2 (T_1_T_1_)^1^ → T_1_ + T_1_

This second step of SF (eqn (2)), the decorrelation of the triplet pair, occurs when there is a change in the coupling between triplets, either through triplet diffusion in a material or through a structural change in a discrete dimer.^4^,^7^,^8^ Although SF was first described in the literature in 1965,^9^ limitations of temporal resolution in transient optical and magnetic resonance spectroscopies, as well as the restricted computation power of quantum chemistry calculations at that time prevented intensive and detailed examination of the states involved. Therefore, interest in SF decreased over the years until 2006, when Hanna and Nozik^10^ pointed out that SF provides a possibility to overcome the Shockley–Queisser limit,^11^ the upper thermodynamic limit of the energy conversion efficiency in single-junction solar cells.

Since then, SF has been extensively studied with a focus on identifying the relevant interacting states and understanding the role of coupling between them. These studies have involved a variety of different molecules like pentacenes,^12^,^13^,^65^ tetracenes,^14^,^15^ hexacenes,^16^,^17^ rylenes,^18^,^19^ carotenoids,^20^,^21^ diketopyrrolopyrroles^22^,^23^ and others^24^–^26^ as monomers in the solid state, concentrated solutions, or as covalent dimers^27^ in dilute solution. For many of these investigations, it is generally accepted that the first step of SF, namely the formation of ^1^(T_1_T_1_), strongly depends on the interacting chromophores, as well as the strength and type of coupling between them. So far, three different mechanisms have been proposed, observed, and studied in-depth. In the direct mechanism,^28^,^29^ the (S_1_S_0_) state transforms directly into ^1^(T_1_T_1_), while in the mediated mechanism this step is facilitated by coupling to virtual higher-lying charge-transfer (CT) states.^30^,^31^ In noncoherent SF, the CT-mediated and direct mechanisms both contribute to the formation of the ^1^(T_1_T_1_) states and an interplay of energetics and electronic coupling determines which mechanism dominates.^32^ This interplay, however, is still poorly understood and is subject to considerable debate. Some pentacene dimers, for example, have been reported to undergo the CT-mediated mechanism, while others proceed *via* the direct mechanism.^29^,^30^ A clear understanding of the factors that dictate the dominant mechanism would help to optimize the design of highly efficient SF chromophores for solar cell applications. The third mechanism, the so-called quantum coherent mechanism, involves a coherent superposition of the (S_1_S_0_) and ^1^(T_1_T_1_) states, sometimes including the CT state, that is produced by electronic coupling directly after excitation.^33^–^35^ Investigations focusing on the second step of SF have shown that in a closely packed environment like the solid state, the correlated triplets can undergo triplet decorrelation as the triplet excitons diffuse away from each other as this reduces the exchange coupling between the triplets.^7^,^26^,^36^ In a dimer, however, the two triplets can only undergo decorrelation if the coupling between the two monomers is small right from the beginning. Such weak coupling typically renders ^1^(T_1_T_1_)–^5^(T_1_T_1_) mixing possible, which additionally facilitates triplet decorrelation. In the case of strong coupling between the chromophores, ^1^(T_1_T_1_) will deactivate by triplet–triplet annihilation (TTA) to the ground state.

The enhancement of spin–orbit coupling induced by a heavy atom, known as the internal heavy-atom effect, was first observed almost 70 years ago by McClure.^37^ Generally speaking, the spin–orbit interaction removes the intercombination selection rule for electronic transitions, Δ*S* = 0, whereby *S* is the total spin quantum number of a molecule or atom.^38^ Spin–orbit coupling is clearly a relativistic effect, but can be described nonrelativistically as the electromagnetic interaction between the spin magnetic moment of the electron orbiting a nucleus with charge *Z* and the Coulomb field of this nucleus.^38^ It is known that the enhancement of the spin-forbidden processes *via* the heavy-atom effect causes a decrease of the singlet excited-state lifetime that is directly linked to a population of the (T_1_) state, and a further quenching of the lifetime of the (T_1_) state. Additionally, the oscillator strength of spin-forbidden T_1_ ← S_0_ absorptions can increase.^39^

Musser *et al.*^40^ reported singlet fission in polythienylenevinylene and its selenophene and tellurophene derivatives. They found no significant differences in the (T_1_T_1_) formation dynamics between the three polymers. However, once the (T_1_T_1_) state is formed, its lifetime was found to depend on the identity of heteroatom. Musser *et al.* attributed this dependence to the heavy-atom effect of the heteroatom.

In 2012, Nguyen and Yip presented pentacene and tetracene dimers and monomers linked to a platinum atom.^41^ They observed a strong fluorescence quenching for all pentacene-platinum compounds; however, the quenching was stronger in the dimers. The tetracene-platinum monomers did not show fluorescence quenching, while the fluorescence of the platinum-bridged tetracene dimer was strongly quenched. They attributed the fluorescence quenching to excitonic coupling. It is quite possible, however, that the fluorescence of the pentacene and tetracene dimers is quenched due to SF. This is a first indication that SF may outcompete the heavy-atom effect of the platinum in such dimers. Motivated by these findings, we designed two pentacene dimers that are linked in different geometries by a platinum atom and a pentacene-platinum monomer. We employed transient absorption spectroscopy to corroborate that the coupling strength of both pentacenes is sufficiently strong to quantitatively produce the correlated triplet pair ^1^(T_1_T_1_) *via* SF on a picosecond timescale. Furthermore, the substantial rates for the first step of SF establish that the two pentacenes are coupled too strongly for ^1^(T_1_T_1_) to undergo spin decoherence facilitated by small geometrical changes. However, we observe quantitative amounts of long-lived triplets. A combination of transient absorption (TA) and EPR spectroscopy allows us to verify that the heavy-atom effect of the platinum enables ^1^(T_1_T_1_)–^3^(T_1_T_1_) and ^1^(T_1_T_1_)–^5^(T_1_T_1_) mixing that facilitates a subsequent population of (T_1_S_0_) and a minor amount of (T_1_+T_1_).

Interestingly, the mechanism of the ^1^(T_1_T_1_) formation for one of the Pt-linked dimers differs from that typically observed for pentacene dimers linked at the central C-atom: while the CT-mediated mechanism is the normal case for pentacene dimers that are linked at the central C-atom,^12^,^30^ at least one of the dimers presented shows clear evidence for the direct SF mechanism, which is usually observed for pentacene dimers that are coupled at their 2-position.^29^ Employing transient IR (fsIR) and TA spectroscopy, supported by quantum chemical calculations, enables us to gain insight in the parameters that influence the first step of SF and in how these can be fine-tuned.

## Results

### Synthesis

**Scheme 1:**
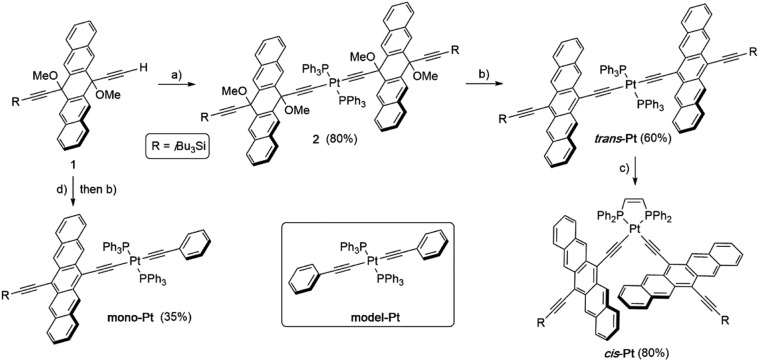
Synthesis of *trans*- and *cis*-acetylides **mono-Pt**, ***trans*-Pt**, and ***cis*-Pt**. Reagents and conditions: (a) *cis*-Pt(PPh_3_)_2_Cl_2_, CuI, *i*Pr_2_NH, THF, rt, 48 h. (b) SnCl_2_·2H_2_O, 10% aq. H_2_SO_4_, THF, rt, 4–5 h. (c) *cis*-1,2-(diphenylphosphino)ethylene, CH_2_Cl_2_, rt, 48 h. (d) *cis*-Pt(PPh_3_)_2_Cl_2_, phenylacetylene, CuI, Et_2_NH, 50 °C, 16.5 h.

The synthesis of Pt-acetylide complexes is well-documented in the literature.^41^–^44^ They are typically formed *via* the reaction of a terminal acetylene with *cis*- or *trans*-PtCl_2_(R_3_P)_2_, giving the thermodynamically more stable *trans*-isomer in both cases when the reaction is conducted at room temperature. Thus, terminal acetylene **1** (ref. 45) reacted with *cis*-Pt(PPh_3_)_2_Cl_2_ in the presence of CuI and *i*Pr_2_NH to give **2** (Scheme 1, see ESI† for details). With **2** in hand, reductive aromatization^46^ with Sn(ii)Cl_2_ and dilute acid furnished *trans*-dimer ***trans*-Pt** in 60% yield. The *trans*-dimer ***trans*-Pt** was then used in a ligand exchange reaction^47^,^48^ with *cis*-1,2-(diphenylphosphino)ethylene to give *cis*-isomer ***cis*-Pt**. The model compound **mono-Pt**, featuring only one pentacene moiety, was assembled in a similar fashion using equal molar amounts of **1** and phenylacetylene in a reaction with *cis*-PtCl_2_(PPh_3_)_2_. Without purification of the resulting intermediate, reductive aromatization with Sn(ii)Cl_2_ gave **mono-Pt** in 35% yield over the two steps (Scheme 1). Finally, phenyl acetylide model compound **model-Pt** was synthesized as reported in the literature.^49^ The products **model-Pt**, **mono-Pt**, ***trans*-Pt**, and ***cis*-Pt** were purified *via* column chromatography and show good solubility in common organic solvents such as THF, CH_2_Cl_2_, and toluene (*ca.* ≥ 8 mg mL^–1^). Compounds **mono-Pt**, ***trans*-Pt**, and ***cis*-Pt** are stable under ambient laboratory conditions for days, although they slowly decompose over several days in solution under exposure to both oxygen and light.

### Establishing the effect of platinum on the electronic structure of pentacene – investigations on **mono-Pt**

**Fig. 1:**
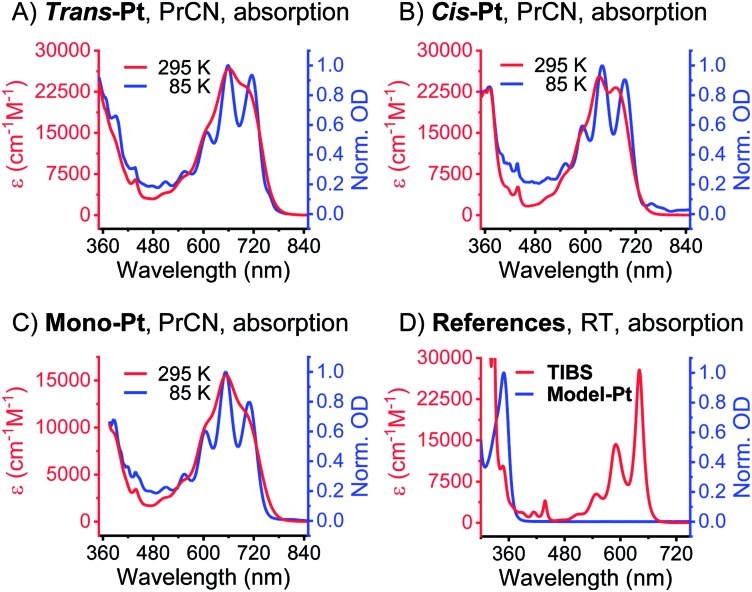
Steady-state absorption spectra of ***trans*-**, ***cis*-**, **mono-**, **model-Pt** and **TIBS**. (A) ***Trans*-Pt** in butyronitrile (PrCN). (B) ***Cis*-Pt** in PrCN. (C) **Mono-Pt** in PrCN. (D) **Model-Pt** and **TIBS** in butyronitrile and at room temperature.

The first insight into the effect of platinum on the electronic properties of the platinum-pentacene derivatives comes from steady-state absorption and fluorescence measurements performed in butyronitrile (PrCN). The steady-state spectra were compared to those of 6,13-bis(tri-isobutylsilylethynyl)pentacene (**TIBS**) and evaluated with respect to the influence of the heavy atom.^45^ The 500–650 nm range of the **TIBS** absorption spectrum is governed by well-resolved vibrational fine structure.^30^ In stark contrast, **mono-Pt**, ***trans*-Pt**, and ***cis*-Pt** all show a broad and rather featureless absorption that ranges up to 800 nm (see Fig. 1A–C). This finding is in sound agreement with the absorption spectra reported by Nguyen and Yip.^41^ Upon cooling to 85 K, an overall sharpening of the absorption features allows for determining the precise position of the maxima in PrCN: 510, 553, 604, 652 and 709 nm for **mono-Pt**; 510, 555, 608, 658 and 715 nm for ***trans*-Pt**; and 510, 553, 592, 640 and 694 nm for ***cis*-Pt**. Of particular interest is the 709 nm maximum for **mono-Pt**, which red-shifts to 715 nm in the case of ***trans*-Pt** and blue-shifts to 694 nm in the case of ***cis*-Pt**. Based on the fact that neither **model-Pt** nor **TIBS** exhibit these long-wavelength absorptions (see Fig. 1D), we considered the potential roles of intramolecular exciton splitting, aggregation, and charge transfer. All of these were, however, ruled out as possible rationales by conducting concentration- and solvent-dependence studies of **mono-Pt** (see Fig. S13 and S14†).

**Fig. 2:**
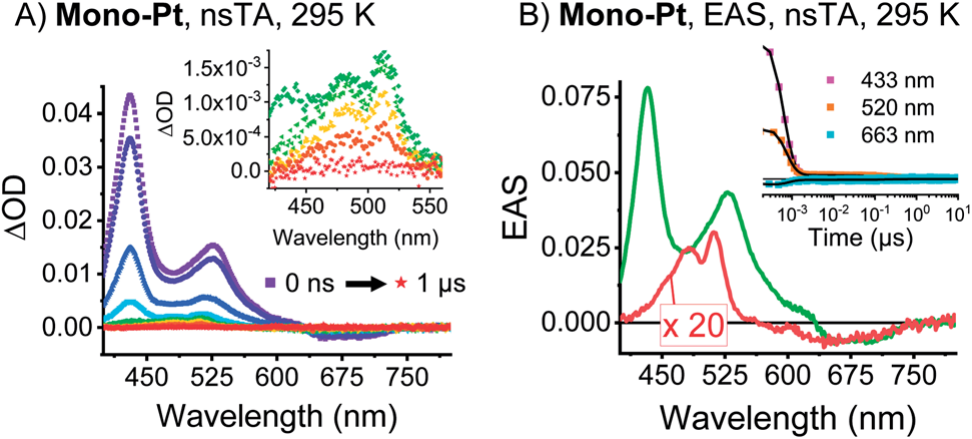
nsTA data of **mono-Pt**. (A) nsTA raw data of **mono-Pt** measured at RT in oxygen-free butyronitrile (excitation at 590 nm). The inset shows a zoom-in on the triplet absorptions. (B) Deconvoluted, evolution-associated spectra (EAS) of the singlet state (green) and the triplet state (red) of **mono-Pt** at room temperature obtained upon global analysis with a sequential model of TA data measured in oxygen-free butyronitrile. See (A) for the raw data. Inset: raw data single-wavelength kinetics and fits to the data.

As documented in Fig. S15† and Tables S1–S2,† the vibronic maxima of the S_2_ ← S_0_ absorptions are of the same energies in **mono-Pt**, ***trans*-Pt**, ***cis*-Pt**, and **TIBS**. In contrast, an additional feature of the S_1_ ← S_0_ absorptions is seen in **mono-Pt**, ***trans*-Pt**, and ***cis*-Pt** at around 700 nm. Thus, we conclude that the origin of the broadening and the additional absorption band to be a distortion of the (S_1_) potential energy surface by the platinum. Next, we performed femtosecond and nanosecond transient absorption (TA) spectroscopy on a dilute solution of **mono-Pt** in PrCN at room temperature to investigate the effect of the platinum linker on the excited state dynamics of pentacene. Upon excitation at 600 nm, an excited state is populated that possesses similar transient absorption features as found for the **TIBS** singlet excited state (S_1_) (see Fig. 2A): two maxima appear, at 430 and 527 nm, concomitant with a broad minimum between 630 and 740 nm, which resembles the broad and featureless ground-state absorption.^30^ The (S_1_) state exhibits an almost quantitative decay to the ground state (S_0_) with a time constant of 0.36 ns. A minor component (∼1%) of (S_1_) undergoes intersystem crossing (ISC) to afford the corresponding triplet excited state (T_1_). A comparison to **TIBS** reveals that the (S_1_) lifetime of **mono-Pt** is quenched by a factor of ∼30, while the triplet yield remains almost constant.^30^ Importantly, the low triplet yield precludes quenching of the (S_1_) state *via* ISC enhanced by a heavy-atom effect of the platinum, as this would lead to a significantly higher triplet yield.^38^ It is likely, that the distortion of the (S_1_) potential energy surface observed in the absorption spectra (*vide supra*) goes hand-in-hand with changes in the (S_1_) lifetime in **mono-Pt** compared to **TIBS**.^50^,^51^

Upon cooling to cryogenic temperatures (85 K), the lifetime of the (S_1_) state increases significantly by a factor of 6.5 to 2.34 ns (see Table S3†). The triplet yield, however, remains unchanged (see Fig. S21A†), and the (T_1_) lifetime increases only slightly by 21% to 370 ns. The (T_1_) state shows maxima at 480 and 511 nm as well as minima at 586 nm and from 610 to 740 nm. This state decays quantitatively to (S_0_) with a lifetime of 305 ns, which indicates a shortening of the (T_1_) lifetime by a factor of ∼100 *versus***TIBS**.^30^ This quenching is most likely caused by enhanced ISC induced by the heavy-atom effect of the platinum.^38^ Thus, it appears that the heavy-atom effect only influences the available triplet population at longer timescales, but does not affect the singlet state as a result of accelerated IC.

**Fig. 3:**
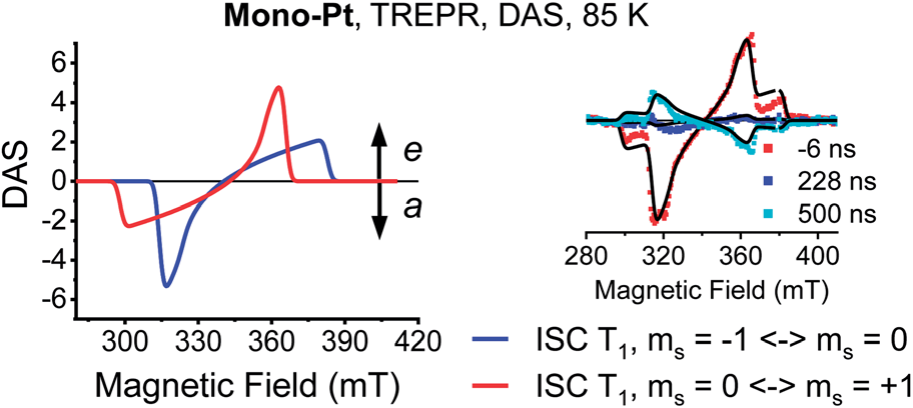
Transient EPR data of **mono-Pt**. TREPR decay-associated spectra (DAS) of the (T_1_) state (–1 ↔ 0 transition, blue; 0 ↔ +1 transition, red) for **mono-Pt** at 85 K in oxygen-free butyronitrile. Inset: overlay of spectra at the indicated time after excitation and the fits (black lines) to the data. Fit parameters: *k*_*z*_ : *k*_*y*_ : *k*_*x*_ = 0.6 : 1.3 : 2, |*D*| = 1190 MHz, *E* = 86 MHz.

Time-resolved EPR (TREPR) measurements give the most insight into the mechanism that leads to triplet formation: ISC or SF. The relative populations of the triplet sublevels are strongly mechanism-dependent, which leads to different polarization patterns for ISC- *vs.* SF-born triplet states. TREPR experiments of **mono-Pt** in PrCN at 85 K confirm that ISC is responsible for populating (T_1_) following laser excitation (7 ns pulses) at 590 nm (Fig. 3). The polarization pattern (e, e, e, a, a, a) (a = absorption, e = emission) for a low-to-high magnetic field analysis is characteristic for a triplet excited state whose origin is ISC.^52^ The zero-field splitting (ZFS), |*D*| = 1190 MHz and *E*/*D* = 0.0723, determined by fitting the TREPR spectrum, agree well with the literature values reported for the pentacene triplet state.^4^,^6^,^53^ This finding corroborates that the formation of (T_1_) in **mono-Pt** is caused by ISC.

### Singlet fission in ***cis*-** and ***trans*-Pt**

**Fig. 4:**
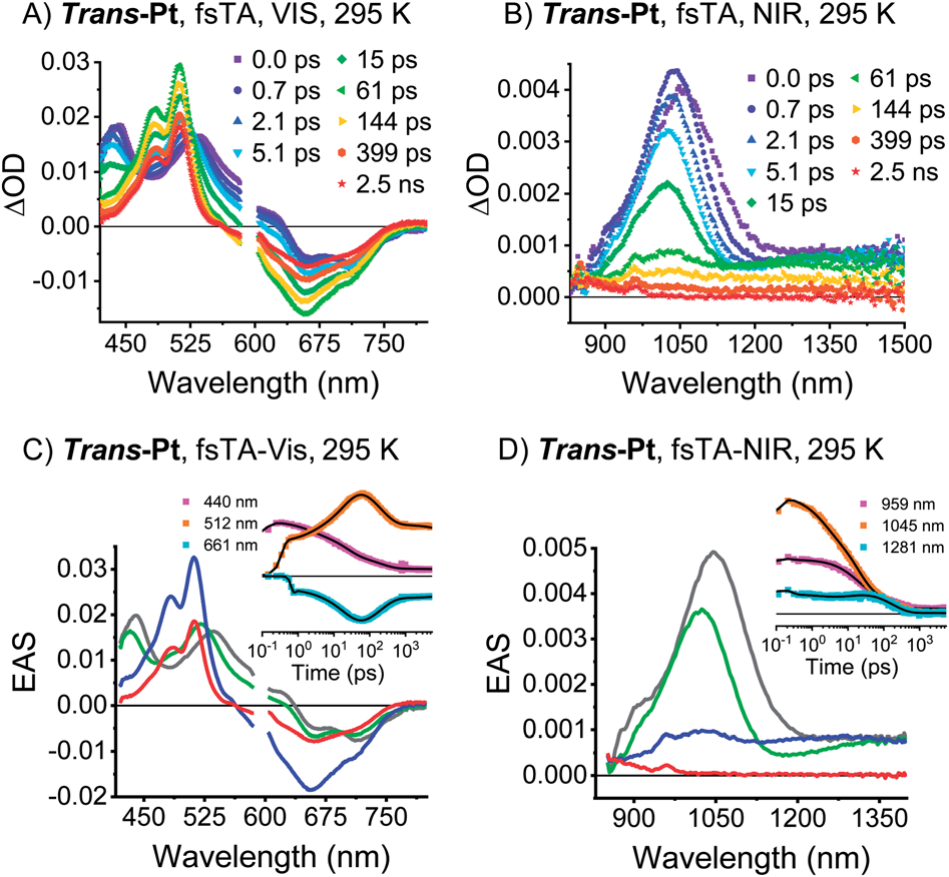
TA data of ***trans*-Pt**. (A and B) fsTA raw data of ***trans*-Pt** measured in oxygen-free butyronitrile (VIS & NIR) at room temperature, excitation at 590 nm. (C and D) EAS of the (S_0_S_1_)* state before solvent relaxation (gray), the (S_0_S_1_) state after solvent relaxation (green), the correlated triplet pair state (T_1_T_1_) (blue) and the decorrelated triplet state (S_0_T_1_) (red) of ***trans*-Pt** obtained upon global analysis with a sequential model of TA data measured in oxygen-free butyronitrile at room temperature. (C) ***Trans*-Pt**, visible. See (A) for the raw data (D) ***trans*-Pt**, NIR. See (B) for the raw data. Insets: raw data single-wavelength kinetics and fits to the data.

TA spectroscopy was performed in dilute solutions at room temperature to investigate whether ***trans*-** and ***cis*-Pt** undergo singlet fission. Upon photoexcitation of ***trans*-Pt**, singlet excited-state (S_1_S_0_) characteristics typical of pentacene derivatives are discernable (Fig. 4): minima in the visible region at 663 and 713 nm as well as maxima in the visible and near-infrared regions at 440, 535, and 1045 nm. For ***cis*-Pt**, minima at 634 and 689 nm evolve simultaneously with maxima at 443, 538 and 1097 nm (Fig. S18†). These features all blue shift by a few nanometers within the first 2 ps for ***trans*-Pt** or 3 ps for ***cis*-Pt**. The analogous blue-shifts in pentacene derivatives were recently linked to solvent rearrangement as a response to a more polar character of (S_1_S_0_) compared to (S_0_S_0_) inferring a coupling of (S_1_S_0_) to a higher-lying CT.^30^

The (S_1_S_0_) states of both compounds are relatively short-lived with lifetimes of 21 and 49 ps for ***trans*-Pt** and ***cis*-Pt**, respectively, and transform into new transients. In ***trans*-Pt**, they possess rather sharp maxima at 480, 515, ∼850, and 958 nm next to broad and featureless maxima at 1028 nm and another spanning from 1200 to 1350 nm. Some of the observed maxima resemble those found for the triplet excited states of **TIBS** and **mono-Pt** and, in turn, we postulate the formation of a correlated triplet pair (T_1_T_1_). ***Cis*-Pt** gives rise to similar maxima at 477, 506, 848, 962 nm and a broad feature at 1069 nm. It is noteworthy that strong, broad maxima in the near-infrared have not, however, been reported for pentacene triplet excited states.^30^ From a closer inspection of the near-infrared spectra we discern similarities between the broad (T_1_T_1_) maxima in the near-infrared and (S_1_S_0_) features in the near-infrared. A likely rationale for this observation is diabatic state mixing. Recently, a similar mixing of (T_1_T_1_), (S_1_S_0_), and a CT states was observed in a covalent slip-stacked terylene-3,4 : 11,12-bis(dicarboximide) dimer.^54^ It is important to emphasize that the diabatic (S_1_S_0_) character of the adiabatic (T_1_T_1_) state is smaller in ***cis*-Pt** than in ***trans*-Pt**, based on the different intensities across the near-infrared region of the TA spectra (compare Fig. 4 and S18†). Ground-state bleaching was detected in addition to the aforementioned maxima. This consists of minima at 656 and 712 nm for ***trans*-Pt** and at 632 and 671 nm for ***cis*-Pt**. Crucially, the ground-state bleaching intensifies during decay of (S_1_S_0_) for ***trans*-Pt** and ***cis*-Pt**. If the two pentacene chromophores per dimer are considered individually, triplet quantum yields well above 100% are deduced. As the shape of the ground-state bleaching changes during the transition from (S_1_S_0_) to (T_1_T_1_) (adiabatic states), we did not only rely on the TA data, but measured in addition transient IR spectra for a detailed calculation of the exact SF quantum yields – *vide infra*. Indeed, SF results in singlet lifetimes that are 6- (***cis*-Pt**) and 15-times (***trans*-Pt**) shorter than the deactivation *via* enhanced IC as observed in **mono-Pt**; this corresponds to an SF quantum efficiency of 85% (***cis*-Pt**) and 94% (***trans*-Pt**). In turn, SF outcompetes the S_1_ → S_0_ transition *via* a conical intersection or any other effects of the heavy atom on the singlet population, *e.g.* enhanced spin–orbit couplings. Based on the observed rates and enhanced ground-state bleaching, the triplet excited state found in ***trans*-Pt** and ***cis*-Pt** is, consequently, confirmed to be a correlated triplet pair (T_1_T_1_). Within a few hundreds of picoseconds, the features of (T_1_T_1_) in the visible region decay to about 50% of their initial amplitudes, while the broad and featureless maxima in the near-infrared region disappear completely. After decay times of 200 ps for ***trans*-Pt** and 624 ps for ***cis*-Pt**, the transient absorption spectra are in excellent agreement with the (T_1_) spectra of **mono-Pt**, **TIBS** and other pentacene derivatives.^30^ This constitutes further evidence for the fact that the adiabatic (T_1_T_1_) state is a linear combination of the (T_1_T_1_) and (S_1_S_0_) diabats, most likely due to interdiabatic electronic couplings.^55^ Interestingly, the lifetimes of 330 ns for ***trans*-Pt** and 609 ns for ***cis*-Pt** (see Fig. S20†) are comparable to the (T_1_) lifetime of **mono-Pt**. We hypothesize that the second triplet species in ***trans*-Pt** and ***cis*-Pt** must be an uncorrelated triplet species, either in the form of (T_1_ + T_1_) or (S_0_T_1_), which exhibits a lifetime that is reduced by the heavy-atom effect. As the yield of this uncorrelated triplet state is about 50% of the yield of the correlated triplet pair state (T_1_T_1_) of both dimers, the uncorrelated state of ***trans*-** and ***cis*-Pt** is most likely (S_0_T_1_). As a complement, room temperature transient femtosecond IR (fsIR) absorption spectroscopy was performed. Dichloromethane solutions of **mono-**, ***trans*-**, and ***cis*-Pt** were excited at 650 nm and probed between 1900 and 2300 cm^–1^ to focus on the alkyne stretching region between 2000 and 2200 cm^–1^.^56^ Overall, **mono-Pt** shows a decay in the fsIR experiments that is similar to that observed in the TA experiments (*vide supra*). Transient (S_1_) and (T_1_) state spectra were identified based on a correlation with the TA data. (Fig. S29†). The (S_1_) spectrum has a maximum at 2017 cm^–1^ and two minima at 2088 and 2122 cm^–1^ as a result of ground-state bleaching. This state decays with a lifetime of 284 ps and ultimately results in an extremely weak ground-state bleaching and essentially no maximum due to (T_1_). The long (T_1_) lifetime, which is beyond the range of the fsIR measurements, hampers meaningful determination with this method.

For ***cis*-Pt**, the best fits were realized when a kinetic model based on four transient states was employed (Fig. S29†) and interpreted with the aid of TA measurements as well as a comparison to the fsIR data of **mono-Pt**. In the applied model, the first transient species is the initially populated (S_1_S_0_)* state. Solvent rearrangement transforms this (S_1_S_0_)* into a solvent-relaxed (S_1_S_0_) state. This state then interconverts into (T_1_T_1_) prior to decorrelation into (S_0_T_1_) as the third and fourth transient species, respectively. The relevant lifetimes for the first three processes are 1.2, 40, and 566 ps, respectively, while exact determination of the lifetime of (S_0_T_1_) was again limited due to the slow decay kinetics that are out of the range of the fsIR measurements. Notably, the two minima at 2088 and 2122 cm^–1^ form the ground-state bleaching. The shape of the ground-state bleaching as an exact mirror image of the ground-state absorption (see Fig. S29†) does not change throughout the (S_1_S_0_)-to-(T_1_T_1_) transformation. This rendered a precise calculation of triplet quantum yields possible (see ESI for details). Considering both pentacenes per dimer individually for (T_1_T_1_), the intensification of the 2130 cm^–1^ minimum corresponds to a triplet quantum yield of 190%. The transient IR spectra of ***cis*-Pt** look very similar for all three states: (S_1_S_0_), (T_1_T_1_), and (S_0_T_1_) (Fig. S29†).

**Fig. 5:**
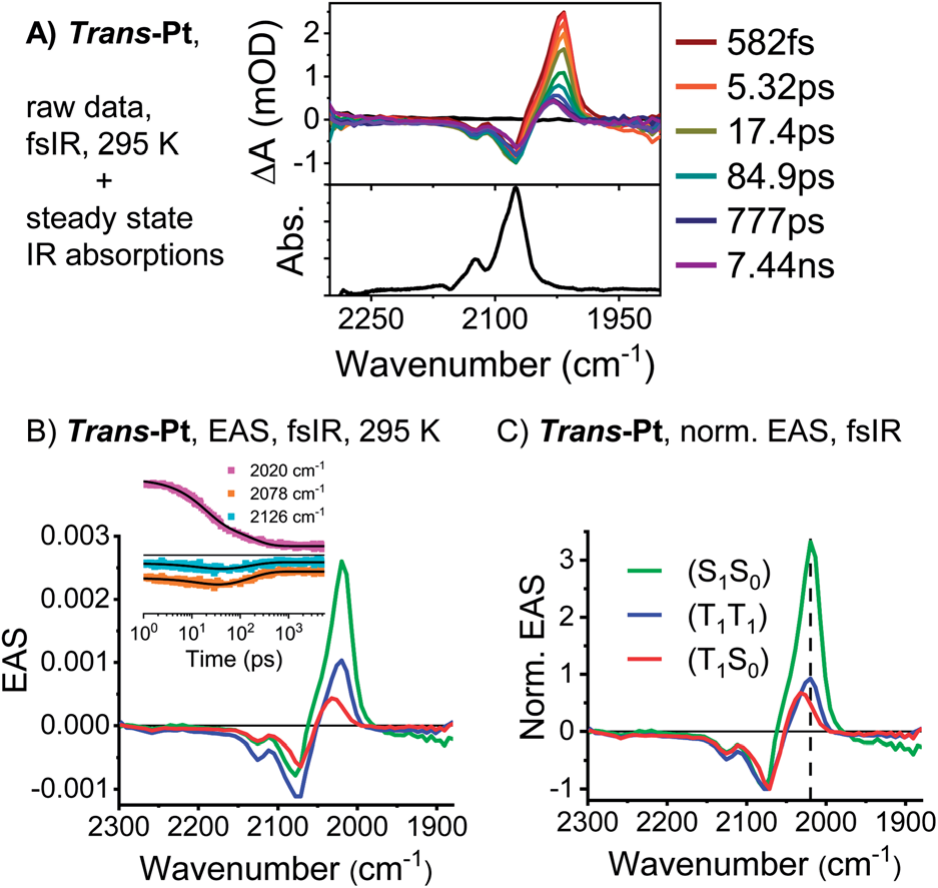
fsIR data of ***trans*-Pt**. (A) Transient and steady-state IR absorption spectra of ***trans*-Pt** measured at room temperature in oxygen-free dichloromethane. The transient IR spectra were measured upon excitation at 650 nm. (B) EAS of the (S_0_S_1_) state after solvent relaxation (green), the correlated triplet pair state (T_1_T_1_) (blue) and the decorrelation triplet state (T_1_S_0_) (red) of ***trans*-Pt** obtained upon global analysis with a sequential model of fsIR data measured in oxygen-free dichloromethane at room temperature. See (A) for the raw data. Insets: raw data single-wavenumber kinetics and fits to the data. (C) EAS of ***trans*-Pt** normalized at the ground-state bleaching. This corresponds to a normalization of the number of excited states, under the assumption that both pentacenes per dimer can be treated individually.

Analysis of the fsIR spectra of ***trans*-Pt** suggests three transient species (see Fig. 5). Compared to the four transient species observed for ***cis*-Pt**, the initial solvent rearrangement is not deconvoluted in the global analysis.§
§This does not imply that for ***trans*-Pt** (S_1_S_0_) does not undergo solvent rearrangement in dichloromethane, but this lifetime might be too short for our detection range (∼500 fs). The remaining transients are the solvent-relaxed (S_1_S_0_), the correlated (T_1_T_1_), and the uncorrelated (S_0_T_1_) states with lifetimes of 18, 159, and ≫5000 ps, respectively. Similar to what we observed for ***cis*-Pt**, the amplitude of the ground-state bleaching strongly intensifies throughout the (T_1_T_1_) formation. Taking for example the intensification of the 2126 cm^–1^ minimum, we derive a triplet quantum yield of 190%. In contrast to ***cis*-Pt**, the fsIR spectra of correlated (T_1_T_1_) and uncorrelated triplet (S_0_T_1_) show differences for ***trans*-Pt**. On the one hand, (T_1_T_1_) possesses a maximum at 2019 cm^–1^ and two minima at 2078 and 2126 cm^–1^, while, on the other hand, the (S_0_T_1_) state features a maximum at 2032 cm^–1^ and two minima at 2071 and 2126 cm^–1^. To this end, (T_1_T_1_) appears as composition of the characteristics of the uncorrelated triplet (S_0_T_1_) and (S_1_S_0_) states; see Fig. 5. The oscillator strength of the maximum relative to that of the two ground-state bleaching minima is almost identical in the case of the correlated (T_1_T_1_) and the uncorrelated triplet (S_0_T_1_) states. In addition, the relative positions of the maximum and the two minima are the same for both (T_1_T_1_) and (S_1_S_0_). Taken the aforementioned into concert, these data corroborate the hypothesis that the adiabatic (T_1_T_1_) possesses diabatic (S_1_S_0_) character in ***trans*-Pt**.

In order to draw comparisons between optical and EPR data, TA was also performed at cryogenic temperatures (85 K) in glassy PrCN (see Fig. S21–S22†). As for **mono-Pt**, the lifetimes of the observed states increase at low temperature for ***cis*-Pt** (see Table S3†), while the overall kinetic model remains identical. For ***trans*-Pt**, the (S_1_S_0_) lifetime does not change upon cooling to 85 K, while other lifetimes increase as for ***cis*-Pt**. Notably, the (T_1_T_1_) lifetimes for both dimers (8.52 ns for ***cis*-Pt** and 1.11 ns for ***trans*-Pt** from the TA measurements) are below the time resolution of the TREPR apparatus.

**Fig. 6:**
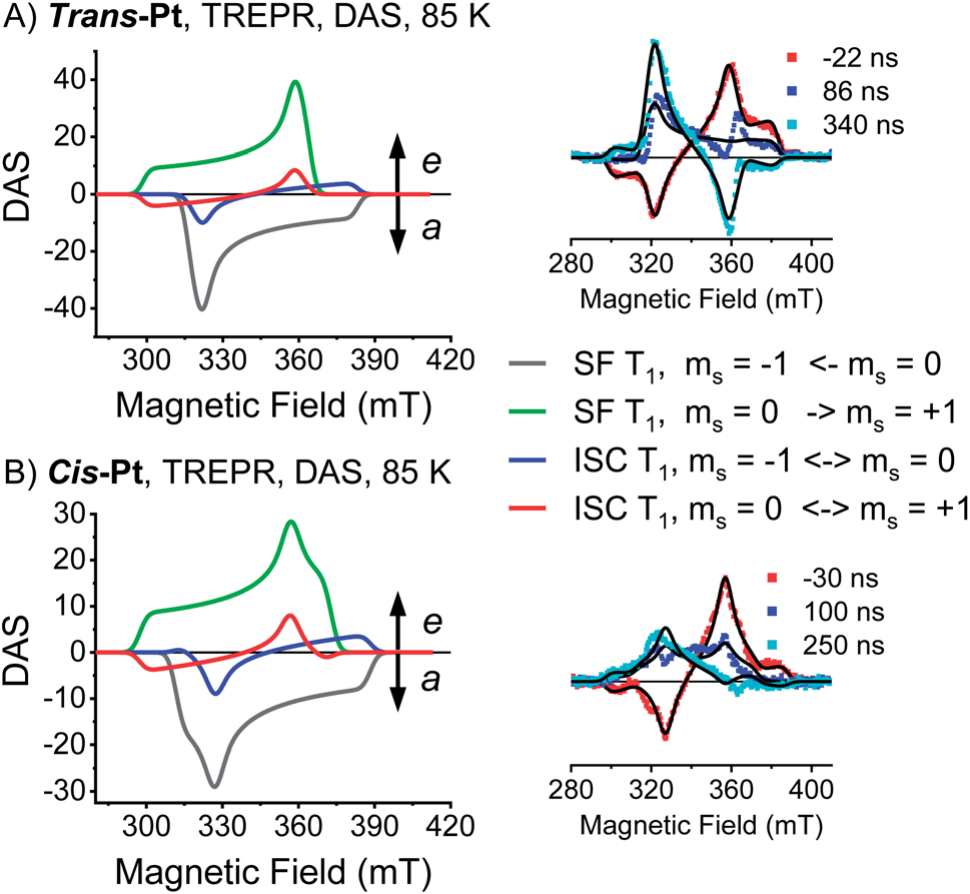
Transient EPR data of ***trans*-** and ***cis*-Pt**. (A) TREPR DAS of the SF-born (T_1_) state (–1 ← 0 transition, gray; 0 → +1 transition, green) and ISC-born (T_1_) state (–1 ↔ 0 transition, blue; 0 ↔ +1 transition, red) for ***trans*-Pt** at 85 K in oxygen-free butyronitrile. Inset: overlay of spectra at the indicated time after excitation and the fits (black lines) to the data. Fit parameters: *k*_*z*_ : *k*_*y*_ : *k*_*x*_ = 0 : 1 : 1, |*D*| = 1190 MHz, *E* = 40 MHz. (B) TREPR DAS of the SF-born (T_1_) state (–1 ← 0 transition, gray; 0 → +1 transition, green) and ISC-born (T_1_) state (–1 ↔ 0 transition, blue; 0 ↔ +1 transition, red) for ***cis*-Pt** at 85 K in oxygen-free butyronitrile. Inset: overlay of spectra at the indicated time after excitation and the fits (black lines) to the data. Fit parameters: *k*_*z*_ : *k*_*y*_ : *k*_*x*_ = 0 : 2.7 : 0.9, |*D*| = 1275 MHz, *E* = 147 MHz.

Consequently, only the long-lived uncorrelated triplet species (S_0_T_1_) can be examined by TREPR spectroscopy. TREPR spectra of ***trans*-** and ***cis*-Pt** were recorded in PrCN at 85 K following photoexcitation at 590 nm with 7 ns pulses (Fig. 6). If the SF-born ^1^(T_1_T_1_) state mixes with ^5^(T_1_T_1_), upon decoherence of ^5^(T_1_T_1_) only the *m*_s_ = 0 states of the (T_1_ + T_1_) are populated, conserving the spin multiplicity. Overall, this leads to a polarization pattern of the triplet EPR spectrum that indicates an excess population in the central *m*_s_ = 0 sublevel of any triplet excited state.^61^ Such a polarization pattern is easy to distinguish from an ISC-born triplet excited state whose sublevels are populated with differing rate constants due to spin–orbit coupling.^52^ Both ***trans*-Pt** and ***cis*-Pt** give rise to triplet species with (e, e, e, a, a, a) spin polarization patterns, which are similar to that of **mono-Pt**. If the triplets were generated through the decorrelation of ^5^(T_1_T_1_), we should expect a (e, a, a, e, e, a) polarization pattern, as has been previously observed.^4^,^6^,^30^ Our results exclude that ^1^(T_1_T_1_)– ^5^(T_1_T_1_) mixing is the dominant pathway for forming long-living triplet states in ***trans*-Pt** and ***cis*-Pt**.

A closer look at the TREPR spectra reveals, however, that the amplitudes of the features from low-to-high field differ from the spectrum of **mono-Pt** and that a fit of the data to a pure ISC-born triplet excited state is impossible. Agreement is reached only when ISC- and SF-born triplet excited states are considered as major and minor pathways, respectively. The latter correlates with an excess population of the central *m*_s_ = 0 sublevel. This points to a change in the mechanism, with respect to the electron spins, for either the generation of (T_1_T_1_) or its subsequent decorrelation in the presence of the heavy-atom.

### The mechanism of the first step of SF, the formation of the correlated triplet pair

In order to further interrogate the mechanism of (T_1_T_1_) formation (Eqn (1)), TA measurements for ***cis*-** and ***trans*-Pt** were performed in solvents of different polarity (see Fig. S25–S28†). Specifically, toluene, THF, and benzonitrile were used in addition to butyronitrile. Triplet yields do not change compared to those observed in butyronitrile. For all solvents, differences in the transient absorption spectra of the correlated and uncorrelated triplets in the NIR region are much more prominent for ***trans*-Pt** (two additional bands at 1000–1050 nm and between 1200 and 1400 nm) than for ***cis*-Pt** (one additional band at 1050–1100 nm). The shape of the transient spectra stays constant for ***trans*-Pt**, while for ***cis*-Pt** the intensity of the additional NIR bands decreases with polarity (see Fig. S25†). Interestingly, the lifetime of the (S_1_S_0_) state shows a polarity dependence for ***cis*-Pt**. It decreases from ∼66 ps in the solvents of low polarity (toluene and THF), to 48 ps in polar solvents (benzonitrile and butyronitrile, see Table 1).

**Table 1:** Lifetimes of the (S_1_S_0_) and (T_1_T_1_) states of ***cis*-** and ***trans*-Pt** in different polarity solvents at room temperature

Solvent	Dipole moment	Dielectric constant	***Cis*-Pt**		***Trans*-Pt**	
			*τ* (S_1_S_0_)	*τ* (T_1_T_1_)	*τ* (S_1_S_0_)	*τ* (T_1_T_1_)
Toluene	1.3 × 10^–30^ cm (ref. 57)	2.38 (ref. 58)	63 ± 3 ps	490 ± 30 ps	18 ± 1 ps	190 ± 10 ps
THF	5.7 × 10^–30^ cm (ref. 57)	7.52 (ref. 58)	69 ± 4 ps	460 ± 20 ps	23 ± 1 ps	200 ± 10 ps
Benzonitrile	10.7 × 10^–30^ cm (ref. 57)	26.5 (ref. 59)	46 ± 2 ps	630 ± 30 ps	20 ± 1 ps	200 ± 10 ps
Butyronitrile	11.7 × 10^–30^ cm (ref. 60)	—	49 ± 3 ps	620 ± 30 ps	21 ± 1 ps	200 ± 10 ps

For ***trans*-Pt**, however, no influence on the solvent polarity could be detected. This finding suggests that the (T_1_T_1_) formation proceeds by slightly different mechanisms in ***cis*-** and ***trans*-Pt**. ***Trans*-Pt** appears to proceed by a direct mechanism, due to the lack of solvatochromic effects. In contrast, for ***cis*-Pt**, the main driving force for SF is mediation by a virtual CT state, as supported by the polarity dependence.

## Discussion

### The mechanism of the first step of SF in ***trans*-** and ***cis*-Pt**

Mechanistic insight into the first step of SF in ***cis*-** and ***trans*-Pt** is based on three independent observations: first, strong and broad maxima have been identified in the near-infrared TA spectra of (T_1_T_1_) (Fig. 4 and S18†). These maxima have not been reported for pentacene triplet excited states and were not observed for the (S_0_T_1_) of ***cis*-** and ***trans*-Pt**. Considering their presence in (S_1_S_0_) of ***cis*-** and ***trans*-Pt** we conclude that the adiabatic (T_1_T_1_) state possesses diabatic (S_1_S_0_) character due to strong interdiabatic electronic couplings between the (T_1_T_1_) and (S_1_S_0_) diabats.

**Fig. 7:**
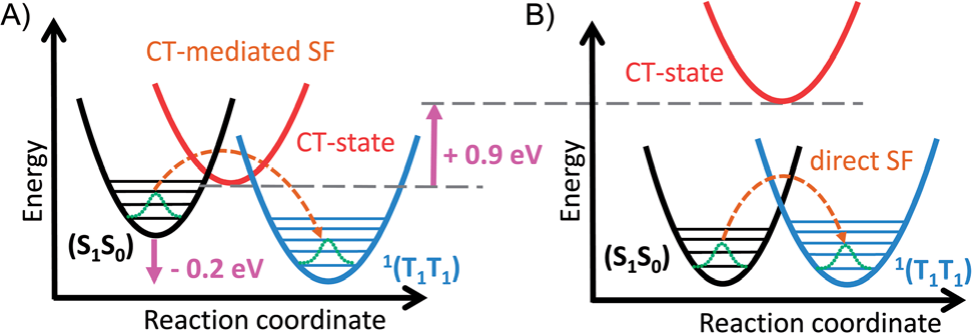
Relative energy level diagram illustrating the influence of the energy shift of the (S_1_S_0_) and the CT-state in ***trans*-Pt**. (A) Energy level diagram in typical 6,6′-linked pentacene dimers. (B) Energy level diagram in ***trans*-Pt**: the (S_1_S_0_) state is lowered by about 0.2 eV compared to other 6,6′-linked pentacene dimers.^4^,^12^,^30^ Compared to ***cis*-Pt**, the lowest CT-state of ***trans*-Pt** is about 0.9 eV higher in energy.

Overall, the maxima in the near-infrared TA spectrum are stronger for ***trans*-Pt** than for ***cis*-Pt**. Second, the (T_1_T_1_) IR spectrum appears for ***trans*-Pt** as a superposition of (S_1_S_0_) and (S_0_T_1_) (Fig. 5). The fsIR measurements were inconclusive for ***cis*-Pt**. Consequently, interdiabatic electronic couplings between the (T_1_T_1_) and (S_1_S_0_) diabats is stronger for ***trans*-Pt**. Third, TA measurements in different solvents reveal a polarity-dependency in terms of the (S_1_S_0_) lifetime for ***cis*-Pt**, but not for ***trans*-Pt**. Importantly, a blue-shift of the NIR maximum of (S_1_S_0_) is seen during the first few picoseconds for ***cis*-Pt** as well as ***trans*-Pt** (Fig. 4 and S23–S26†). A likely rationale is a coupling of (S_1_S_0_) to a higher-lying CT state in both cases.^30^ The CT state and the coupling to it has, however, no appreciable influence on the SF rate for ***trans*-Pt**. Considering the differences in the transient TA and IR experiments of ***cis*-** and ***trans*-Pt**, we conclude that SF in ***trans*-Pt** follows the direct mechanism, while SF in ***cis*-Pt** is governed by the CT-mediated mechanism. It is important to appreciate that (T_1_T_1_)-formation *via* a superexchange CT-mediated mechanism is the normal case for pentacene dimers that are linked at the central 6-position of the chromophore.^12^,^30^ Note that, although CT states in perfect static symmetrical conformations would be a 1 : 1 combination of the two possible (+– and –+) degenerate CT states, and therefore not be polar, thermal motion and solvent effects lead to symmetry breaking in the real situation, so that polar states and solvatochromism are expected. In fact, simply optimizing the geometries of ***cis*-Pt** and ***trans*-Pt** without symmetry constraints with density functional theory gives slightly unsymmetrical geometries. If these are used for semiempirical (CIS/PM6) molecular-orbital configuration interaction calculations of the excited states gives a series of clear CT states that lie approximately 0.9 eV lower in energy for ***cis*-Pt** than for ***trans*-Pt** and that are stabilized by approximately 1.6 eV relative to the ground state in benzonitrile. Details of the calculations are given in the ESI.† So the question becomes: why does ***trans*-Pt** follow the direct mechanism? It is likely that the interdiabatic electronic coupling between the (T_1_T_1_) and (S_1_S_0_) diabats is sufficiently strong for ***trans*-Pt** to promote direct SF and that the CT-state is too high in energy to really impact the SF process. This hypothesis also explains why ***cis*-Pt** behaves slightly different than ***trans*-Pt**, as interdiabatic electronic couplings are weaker for ***cis*-Pt** than for ***trans*-Pt** and the CT state is lower in energy for ***cis*-** than for ***trans*-Pt**. The cause of the direct interdiabatic electronic couplings between the (T_1_T_1_) and (S_1_S_0_) diabats may lie in the distortions of the potential energy surface introduced by the platinum – in line with a 50 nm red-shift of the ground-state absorption – as this lowers the energy of the (S_1_S_0_) state. For ***trans*-Pt**, the ground-state absorptions are even more strongly red-shifted, that is, by 20 nm, than for ***cis*-Pt**. This corresponds to a lowering of the (S_0_S_1_) energy of ***trans*-Pt** in comparison to **TIBS** by about 0.2 eV.^30^ It is likely that this red-shift lowers the energy gap between the (S_1_S_0_) and (T_1_T_1_) states and consequently reduces the SF exothermicity in ***trans*-Pt** and ***cis*-Pt** compared to other pentacene dimers. Thus, the reduced exothermicity in combination with the increase of the CT-energy is postulated to be essential for a transition from CT-mediated SF to the direct mechanism (see Fig. 7). Though the two dimers undergo slightly different mechanisms for the formation of (T_1_T_1_), the subsequent decay of (T_1_T_1_) proceeds with similar rates for both ***trans*-** and ***cis*-Pt** and leads to a comparable yield of uncorrelated triplet for both dimers.

### Influence of the heavy-atom effect on the triplet decorrelation in ***cis*-** and ***trans*-Pt**

The most direct way to interrogate the heavy-atom effect of platinum on SF of ***trans*-** and ***cis*-Pt** involves comparisons with recently published pentacene dimers without heavy atoms: ***m*-3**, ***o*-3**, ***p*-3**, **Nc-*m***, and **Nc-*p*** (see Fig. S30†). The closest agreement in terms of (S_1_S_0_) lifetimes is noted for ***m*-3**, a dimer in which the two pentacenes are linked *via* a phenylene spacer in a *meta*-arrangement.^12^ Compound ***m*-3** shows a biphasic (S_1_S_0_) decay with lifetimes of 16 and 63 ps. Notably, the (S_1_S_0_) lifetimes of nonconjugated **Nc-*m*** and **Nc-*p*** are two or three orders of magnitude longer than what is observed for ***trans*-Pt** and ***cis*-Pt**. This implies that the electronic coupling between both pentacenes is rather strong and comparable to the situation in ***m*-3**. Still, the (T_1_T_1_) state in ***m*-3** is subject to a quantitative geminate triplet–triplet annihilation, while both ***trans*-Pt** and ***cis*-Pt** form a long-living triplet species. At first glance this is a contradiction. The exchange interaction (*J*) increases with the strength of the coupling between both pentacenes and controls the energetic splitting between ^1^(T_1_T_1_), ^3^(T_1_T_1_) and ^5^(T_1_T_1_) states.^4^ A large *J* minimizes the mixing of ^1^(T_1_T_1_) with ^5^(T_1_T_1_) and, in turn, triplet–triplet annihilation outcompetes decorrelation. This leads to the following approximation of the binding energy of the two triplets^62^

3 *E*_b_ ≈ *E*[(T_1_T_1_)^5^] – *E*[(T_1_T_1_)^1^]

**Fig. 8:**
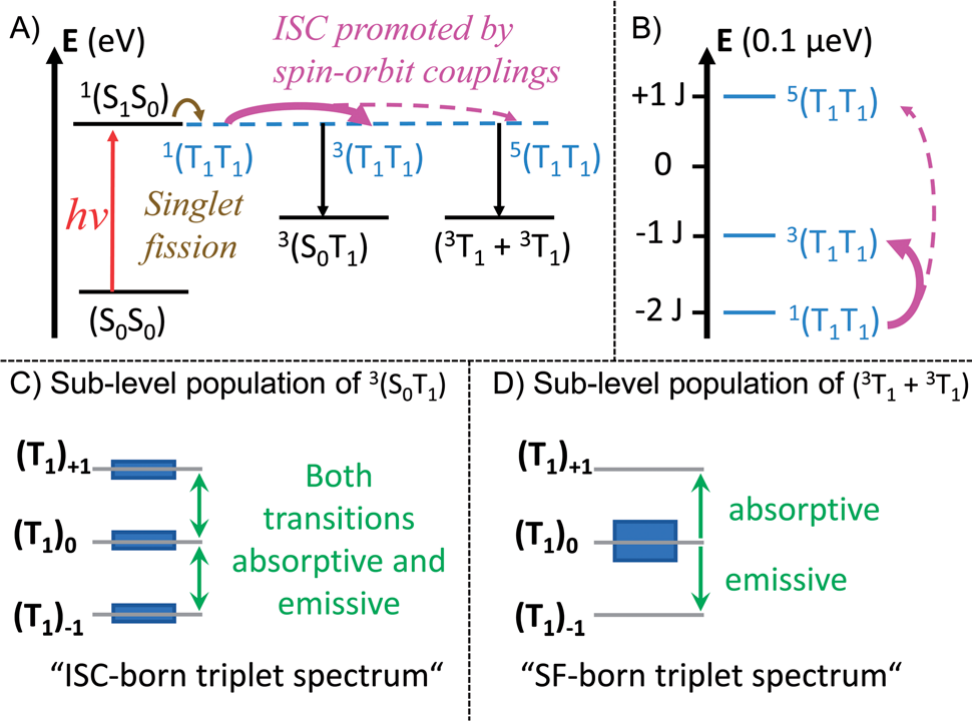
Mechanistic model illustrating the influence of the heavy-atom effect on the correlated triplet pair. In a strongly coupled SF dimer the mixing of the initially formed ^1^(T_1_T_1_) with ^5^(T_1_T_1_) or ^3^(T_1_T_1_) is rather weak. If a heavy atom is implemented in such a system, enhanced spin–orbit coupling enables population transfer from ^1^(T_1_T_1_) to ^3^(T_1_T_1_) and ^5^(T_1_T_1_). (A) Mechanistic model of the singlet fission process in ***trans*-** and ***cis*-Pt**. The energy axis is shown for a TA experiment with an excitation wavelength of ∼600 nm (∼2 eV). (B) Zoom-in on the different spin levels of the correlated triplet pair. Energy splitting of the spin levels (*S*) in the strong exchange limit, according to *E*(*S*) = 0.5*J*[*S*(*S* + 1) – *S*_A_(*S*_A_ + 1) – *S*_B_(*S*_B_ + 1)], with *S*_A_ = *S*_B_ = 1.^63^ The energy axis is shown for an EPR experiment using a typical X-band spectrometer with a frequency of 8–12 GHz (∼33–50 μeV) and a center field of ∼350 mT. This results in a zoom-in on the energy scale by ∼7 orders of magnitude compared to A. (C) Qualitative relative populations of the *m*_s_ sub-levels of (S_0_T_1_). The resulting EPR spectrum possesses a shape typical for ICS-born triplet states. (D) Qualitative relative population of the *m*_s_ sub-levels of (T_1_ + T_1_). The resulting EPR spectrum possesses a shape typical for SF-born, decorrelated triplet states.

A prime example is ***m*-3** where the fate of (T_1_T_1_) is geminate triplet-triplet-annihilation to afford prompt and quantitative recovery of the ground state (S_0_S_0_).^12^ All of these considerations lead us to conclude that the heavy-atom effect of platinum in ***trans*-Pt** and ***cis*-Pt** promotes the formation of free, uncorrelated triplets. The mechanism for this process is not clear from the transient optical data, however, the transient EPR spectroscopy provides clues. From the TA and fsIR data, SF was established as the dominant mechanism for triplet formation based on the triplet quantum yields for (T_1_T_1_). Moreover, quantitative formation of uncorrelated triplets (S_0_T_1_) was determined in TA experiments of ***cis*-** and ***trans*-Pt**. Considering that less than 1% of (S_1_) undergoes ISC to yield (T_1_) for **mono-Pt**, it is clear that the high uncorrelated triplet (S_0_T_1_) population observed in ***cis*-** and ***trans*-Pt** cannot be generated directly from (S_0_S_1_) *via* ISC. The TREPR experiments, however, suggest that ISC dominates the triplet excited state formation in ***trans*-Pt** and ***cis*-Pt**. Taking the high triplet yields observed for ***trans*-** and ***cis*-Pt** into account, coupled with involvement of an ISC mechanism, a reasonable explanation is that the correlated triplet pair (T_1_T_1_), and not (S_0_S_1_), is the direct precursor to triplet formation. Thus, while the heavy-atom effect does not appear to significantly influence the singlet decay kinetics, the effect is still the primary agent dictating dynamics after singlet fission. Such a scenario implies a ^1^(T_1_T_1_)–^3^(T_1_T_1_) mixing by means of enhanced spin–orbit coupling. In turn, the ^3^(T_1_T_1_) state will decay into (S_0_T_1_) and will possess a polarization pattern different from a typical SF-born triplet.^64^ If ^3^(T_1_T_1_) decays with a larger rate than with which it is formed, no observable buildup of ^3^(T_1_T_1_) should be discernable. This is a likely scenario, as the destabilization of the triplet pair is expected to be fairly large in the strongly coupled ^3^(T_1_T_1_) triplet pairs of ***trans*-** and ***cis*-Pt**. The same mechanistic consideration is applicable for the minor triplet excited state component with a SF-born spin polarization pattern. In particular, enhanced spin–orbit coupling makes population of the ^5^(T_1_T_1_) state possible, although the large *J* value precludes conventional spin-mixing. Subsequently, ^5^(T_1_T_1_) populates (T_1_ + T_1_) *via* mixing with *m*_s_ = 0, and an excess population of the *m*_s_ = 0 sublevel is realized. Our mechanistic view is summarized in Fig. 8 which depicts the influence of the heavy-atom effect on the correlated triplet pair (T_1_T_1_). Since the yield *via* ISC is so low for **mono-Pt** – even at low temperature – we do not account for triplet formation by ISC from the (S_1_S_0_) state.

### Influence of the relative spatial orientation of the pentacenes and the point of attachment to the platinum

A comparison of ***trans*-** and ***cis*-Pt** gives interesting insight into the influence of the point of attachment to the platinum. A faster SF rate in ***trans*-Pt** than in ***cis*-Pt** corroborates the notion that the orbital overlap between the pentacenes is stronger for the *trans*- than for the *cis*-orientation. As the through-space couplings are expected to be stronger for ***cis*-** than for ***trans*-Pt**, the only explanation for this is that the platinum allows for through-bond couplings which are stronger in the *trans*- orientation than in the *cis*-orientation. A partially extended π-system over the platinum in the excited state that is more extensive in the *trans*-orientation would explain the impact of the platinum on the SF rate. Moreover, this provides a rationale for the distortion of the potential energy surfaces of pentacene (T_1_T_1_) state induced by the platinum which is also significantly stronger for ***trans*-** than for ***cis*-Pt**. Additionally, this hypothesis is well in line with the observation that in ***trans*-Pt**, the (T_1_S_0_) formation that is induced by the heavy atom effect of the platinum was found to be about 3 times faster than in ***cis*-Pt**. As such, we conclude that both dimers possess an extension of the pentacene π-system over the electronic system of the platinum, at least in their excited states. This extension is stronger in the *trans*- than in the *cis*-compound.

## Conclusions

The utilization of two platinum-bridged pentacene dimers that show efficient singlet fission allows us to study the influence of ISC on the transient states that are involved in SF. We have demonstrated, that a strongly coupled correlated triplet pair state ^1^(T_1_T_1_) can undergo ^1^(T_1_T_1_)–^3^(T_1_T_1_) and ^1^(T_1_T_1_)–^5^(T_1_T_1_) mixing facilitated by enhanced spin–orbit couplings to yield a free triplet, namely (S_0_T_1_), as well as a small amount of (T_1_ + T_1_). Combining transient IR, TA and TREPR data enables us to establish that the effect of enhanced spin–orbit couplings on the (T_1_T_1_) state are independent of its formation. Moreover, we demonstrate how strong interdiabatic electronic couplings between the (T_1_T_1_) and (S_1_S_0_) diabats in combination with an increase of the CT-energy facilitate a direct SF, which outcompetes a CT-mediated SF in a pentacene dimer. As such, we provide insight in the factors that influence the mechanism of (T_1_T_1_) formation in pentacene dimers.

## Conflicts of interest

There are no conflicts to declare.

## Supplementary Material

Supplementary information
