# Supplementary material for: Influence of the heavy-atom effect on singlet fission: a study of platinum-bridged pentacene dimers
Source: Chem Sci. 2019 Oct 21;10(48):11130–40. doi: 10.1039/c9sc04410h (PMC7069226; doi:10.1039/c9sc04410h)
Supplement: Supplementary file 1 [file SC-010-C9SC04410H-s001.pdf]

Electronic Supplementary Material (ESI) for Chemical Science.
This journal is © The Royal Society of Chemistry 2019

Electronic Supplementary Information

for

Influence of the Heavy-Atom Effect on Singlet Fission: A Study of Platinum-Bridged Pentacene Dimers

Bettina S. Basel[a*], Ryan M. Young[b*], Matthew D. Krzyaniak[b*], Ilias Papadopoulos[a*], Constantin Hetzer[c], Yueze Gao[d], Nathan T. La Porte[b], Brian T. Phelan[b], Tim Clark[e], Rik R. Tykwinski[d], Michael R. Wasielewski[b], and Dirk M. Guldi[a]
*Authors contributed equally.

[a]Department of Chemistry and Pharmacy & Interdisciplinary Center for Molecular Materials (ICMM), Friedrich-Alexander-Universität Erlangen-Nürnberg (FAU), Egerlandstrasse 3, 91058 Erlangen, Germany.
[b]Department of Chemistry and Institute for Sustainability and Energy at Northwestern (ISEN), Northwestern University, Evanston, IL 60208-3113, USA.
[c]Department of Chemistry and Pharmacy & Interdisciplinary Center for Molecular Materials (ICMM), Friedrich-Alexander-Universität Erlangen-Nürnberg (FAU), Nikolaus-Fiebiger-Strasse 10, 91058 Erlangen, Germany.
[d]Department of Chemistry, University of Alberta, Edmonton, Alberta, T6G 2G2 Canada.
[e]Department of Chemistry and Pharmacy & Computer-Chemistry-Center (CCC), Friedrich-Alexander-Universität Erlangen-Nürnberg (FAU), Nägelsbachstrasse 25, 91052 Erlangen, Germany.

Correspondence to: dirk.guldi@fau.de, m-wasielewski@northwestern.edu, rik.tykwinski@ualberta.ca, tim.clark@fau.de

Contents

General procedures and methods 3
- Synthesis 3
- Spectroscopy 3
- Modeling 5

Supplemental synthesis procedures 7

Supplemental photophysical characterization 23
- Analysis methods of the transient data 23
  - Analysis of the TA data with sequential global analysis 23
  - Analysis of the fsIR data 23
  - Calculation of the triplet quantum yields 23
  - Analysis of the TA data with target analysis 23
  - Analysis of the TREPR data 24
- Supplemental photophysical data 25
  - Supplemental steady-state characterization 25
  - Supplemental transient absorption data in butyronitrile 30
  - Global target analysis 37
  - Supplemental polarity-dependent characterization 40
  - Supplemental transient and steady-state IR data 44
  - Comparison of *trans*- and *cis*-Pt with other covalent pentacene dimers 45

Supplemental theory results 46
- HF/LANL2DZ-optimized geometries 46
  - *Trans*-Pt 46
  - *Cis*-Pt 51
- PM6 (CIS=96) calculated spectra 56
  - *Trans*-Pt 56
  - *Cis*-Pt 66

References 71

General procedures and methods

Synthesis

Reagents were purchased reagent grade from commercial suppliers and used without further purification. THF was dried under nitrogen in a commercial solvent purification system (LC Technology Solutions INC.). $MgSO_4$ was used as the drying reagent after aqueous work-up. ^{1}H, ^{13}C, and ^{31}P NMR spectra were recorded on a Bruker Advance 300 (^{1}H: 300 MHz, ^{31}P: 121 MHz), a Bruker Advance 400 (^{1}H: 400 MHz, ^{13}C: 100 MHz), and an Agilent/Varian Inova four-channel 500 (^{1}H: 500 MHz, ^{13}C: 126 MHz, and ^{31}P: 202 MHz). NMR spectra were recorded at ambient probe temperature. For simplicity, the coupling constants of protons in all ^{1}H spectra have been reported as pseudo first-order when possible, even though they can be higher-order (ABC, ABX, AA'XX') spin systems, and coupling constants have been reported as observed. UV/Vis measurements were carried out on a Varian Cary 5000 UV-Vis-NIR spectrophotometer or a Cary-400 spectrophotometer at room temperature. IR spectra were recorded on a Varian 660-IR spectrometer as solids in ATR mode or on a Thermo Nicolet 8700 FTIR spectrometer and continuum FTIR microscope as a film. Melting points were measured with a Melting Point M-560 instrument. Differential scanning calorimetry (DSC) measurements were made on a Mettler Toledo TGA/STDA 851e/1100/SF. TLC analyses were carried out on TLC plates from Macherey-Nagel (ALUGRAM® SIL G/UV254) and visualized via UV-light (264/364 nm) or standard coloring reagents. Column chromatography used Silica Gel 60M (Merck).

Compounds **1**[1] and **model-Pt**[2, 3] were synthesized as previously reported.

Spectroscopy

All solvents were purchased from commercial suppliers and used without further purification.
Steady-state UV-vis absorption spectra were acquired at room temperature (RT) using a Perkin Elmer Lambda 2 spectrometer. Steady-state fluorescence spectra were carried out at a FluoroMax3 spectrometer from Horiba in the visible detection range (RT). Fluorescence quantum yields were measured relative to a zinc phthalocyanine used as a reference compound.[4]

Fourier transform infrared spectroscopy (FTIR) was performed with a Shimadzu IRAffinity-1 spectrometer. A liquid demountable cell (Harrick Scientific) with 2.0 mm thick CaF_2 windows and a 500 µm Teflon spacer was used to investigate the dissolved samples.

Femtosecond (fs) and nanosecond (ns) transient absorption (TA) experiments (RT and 85 K) were carried out using the EOS System, a broadband pump-probe sub-nanosecond transient absorption

spectrometer, with an integrated customized HELIOS System, broadband pump-probe femtosecond transient absorption spectrometer from Ultrafast Systems, Inc. The transient absorption pump-probe detection system utilizes shot-by-shot acquisition with balanced detection. Balanced detection is accomplished by using the probe-reference method, where the probe beam is split into two before passing through the sample. While one of the two probe beams travels through the sample, the other is sent directly to the reference spectrometer that monitors the fluctuations in the probe beam intensity. For HELIOS (fsTA) measurements in butyronitrile, a regeneratively amplified Ti:sapphire Spitfire Pro fs laser system (Spectra-Physics Tsunami oscillator/Spitfire amplifier: output 827 nm, 1 kHz, 100 fs pulse width, standard deviation <0.5 %) was used and the excitation wavelengths were generated with a laboratory-constructed collinear optical parametric amplifier (standard deviation <2 %), described previously.[5] For the HELIOS measurements (fsTA) in all other solvent (benzonitrile, toluene and THF), an amplified Ti:Sapphire CPA-2110 fs laser system (Clark MXR: output 775 nm, 1 kHz, 150 fs pulse width, standard deviation <0.5 %) was used. The excitation wavelengths were generated with a noncolinear optical parametric amplifier (NOPA, Clark MXR, standard deviation <2 %). For all HELIOS (fsTA) measurements, the white light probe was generated in sapphire crystals (standard deviation <2 %).

For nsTA measurements two independent pulsed light sources are used: the same laser source for pumping as in HELIOS, and a pulsed supercontinuum laser (output 350–2200 nm, 2 kHz, 700 ps^{-1} ns pulse width, standard deviation <1 %) as white light source for probing as part of the EOS spectrometer.

Low temperature (LT) nanosecond (ns) transient absorption (TA) experiments (85 K) were carried out using the same customized transient absorption pump-probe detection systems as described above. Samples were placed in a liquid nitrogen cryostat (VNF-100, Janus Research Company). Samples for nsTA at 85 K were prepared in an N_2-filled glovebox. The solutions were loaded into an optical sample cell consisting of two quartz windows separated by a 2 mm PTFE spacer, and the cell was sealed under N_2. Low temperature samples in butyronitrile (PrCN) were cooled rapidly to 85 K by flooding the cryostat with liquid N_2 to produce a frozen glass.[6, 7]

Femtosecond transient mid-infrared absorption (fsIR) spectroscopy was accomplished using a previously described instrument.[8] Briefly, the output of an amplified Ti:sapphire laser system (Spectra-Physics Solstice, output: 800 nm, 1 kHz, ~100 fs) was split to pump two collinear optical parametric amplifiers (TOPAS-C, Light-Conversion, LLC) to generate the visible pump (650 nm, 2 μJ/pulse) and mid-IR probe pulses (1900–2300 cm^{-1}), respectively. Pump and probe beams were directed into an N_2-purged commercial spectrometer (customized HELIOS-FIRE, Ultrafast Systems LLC) that utilizes a HgCdTe detector. Samples with a maximum optical density of 1.5 at the excitation wavelength were

prepared were prepared in an N_2-filled glovebox and contained in a liquid demountable cell (Harrick Scientific) with 2.0 mm thick CaF2 windows and a 500 μm Teflon spacer. Samples were rastered normal to both pump and probe beams in a randomized pattern to reduce the effect of localized heating or sample degradation.

For fsTA, nsTA and fsIR, the pump beam polarization was spatially randomized using a commercial depolarizer (DPU-25-A, Thorlabs, Inc.) to eliminate the influence of orientational dynamics.

Femtosecond transient absorption data were fitted using multiwavelength analysis with Origin (OriginLab, Northampton, MA) or global analysis. The global fitting was performed with the open-source software package Glotaran.[9-11] Glotaran is a free, Java-based graphical user interface to the R package TIMP, developed for global and target analysis of time-resolved spectroscopy. The wavelength dependent character (dispersion) of the instrument response function (IRF) was modeled and taken into account.

EPR measurements were made at the X-band (~9.5 GHz) using a Bruker Elexsys E680-X/W EPR spectrometer outfitted with a split ring resonator (ER4118XMS3). Measurements were performed at 85 K using an Oxford Instruments CF935 continuous flow cryostat with liquid N_2, respectively. Time-resolved EPR measurements were performed following photoexcitation with 7 ns, 3.2 mJ, 590 nm pulses using the output of an optical parametric oscillator (Spectra-Physics Basi-scan), pumped with the output of a frequency tripled Nd:YAG laser (Spectra-Physics Quanta-Ray Lab 150).
Transient EPR spectra were collected following laser photoexcitation, the transient magnetization was detected in quadrature under CW microwave irradiation (5 mW), without field modulation. Sweeping the magnetic field gave 2D spectra versus both time and magnetic field. For each kinetic trace, the signal acquired prior to the laser pulse was subtracted from the data. Kinetic traces recorded at magnetic field values off resonance were considered background signals, whose average was subtracted from all kinetic traces. The spectra were processed in Matlab (The Mathworks, Natick, MA, USA) using house written scripts and fit using EasySpin.[12]

Modeling

The geometries of the *cis*- and *trans*-compounds were optimized at the RHF/LANL2DZ[13, 14] level with Gaussian16.[15] The X-ray structure served as the starting point for the *trans*-compound and the initial geometry for the *cis* was built from the structure thus obtained. The geometries thus obtained were used for semiempirical configuration interaction calculations with single excitations (CIS) using the PM6 Hamiltonian[16] with 48 occupied and 48 virtual orbitals as the active space. The spectrum of the *trans*-

compound was also calculated in benzonitrile solvent using the polarizable continuum model[17] without the dispersion contribution. We were unable to calculate a spectrum for the *cis*-compound in solution because it did not give a suitable cavity. All semiempirical calculations used the in-house version of VAMP.[18]

Supplemental synthesis procedures

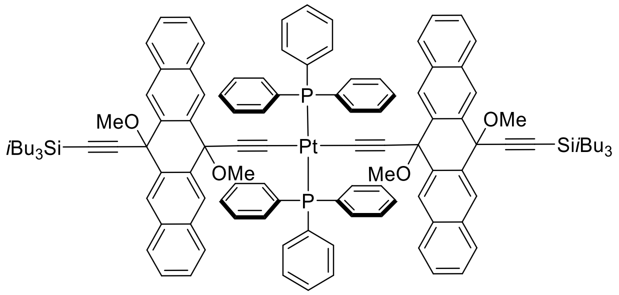

Compound **2**: Acetylene **1** (0.30 g, 0.51 mmol) and *cis*-$Pt(PPh_3)_2Cl_2$ (0.12 g, 0.15 mmol) were suspended in dry, deoxygenated THF (50 mL) and dry, deoxygenated $i$$Pr_2NH$ (30 mL). CuI (0.0023 g, 0.012 mmol) was added and the mixture was allowed to stir for 48 h at room temperature. Water was added (100 mL), and the aqueous phase extracted with CH_2Cl_2 (3 x 50 mL). The combined organic phases were washed with brine (100 mL), dried ($MgSO_4$), and the solvent removed *in vacuo*. Recrystallization from CH_2Cl_2 (5 mL) and MeOH (50 mL) at 5 °C afforded compound **2** (0.227 g, 80%) as a yellowish solid that is unstable on silica gel. Mp 182–184 °C (decomp). IR (ATR, solid state) 3054 (w), 2950 (m), 2866 (m), 2165 (m), 2120 (m), 1434 (m) cm^{-1}. ^{1}H NMR (400 MHz, $CDCl_3$) δ 8.21 (s, 4H), 7.93 (s, 4H), 7.84 (d, J = 7.7 Hz, 4H), 7.72–7.67 (m, 12H), 7.51 (d, J = 7.6 Hz, 4H), 7.46–7.39 (m, 8H), 6.86–6.95 (m, 18H), 2.59 (s, 6H), 2.18 (s, 6H), 1.68 (nonet, J = 6.6 Hz, 6H), 0.78 (d, J = 6.6 Hz, 36H), 0.48 (d, J = 6.9 Hz, 12H). ^{13}C NMR (100 MHz, $CDCl_3$) δ 135.4, 134.9 (pseudo-t, $J_{C–P}$ = 6.1 Hz), 133.7, 132.9, 132.7, 131.3 (pseudo-t, $^{1}J_{C–P}$ = 29.5 Hz), 129.8, 128.3, 128.1, 127.8, 127.6 (pseudo-t, $J_{C–P}$ = 5.3 Hz), 126.5, 125.6, 125.58, 109.2, 89.1, 75.8, 73.4, 51.2, 50.6, 26.2, 24.9, 24.8 (two signals coincident or not observed). ^{31}P NMR (121 MHz, CD_2Cl_2) δ 18.4 (pseudo-t, $J_{P–Pt}$ = 2636 Hz). APPI HRMS calcd for $C_{115}H_{117}O_3P_2{}^{195}PtSi_2$ ($[M – OMe]^+$) 1858.7659, found 1858.7684.

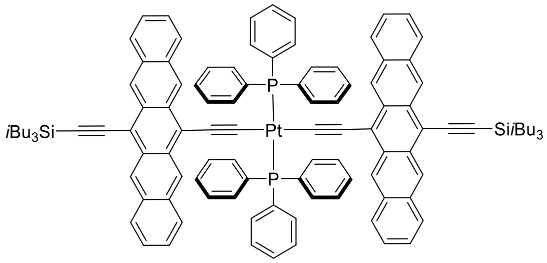

Compound ***trans*-Pt**: To a solution of **2** (0.15 g, 0.079 mmol) in dry, deoxygenated THF (15 mL) was added $SnCl_2$•$2H_2O$ (0.14 g, 0.62 mmol) and 10% H_2SO_4 (0.5 mL) at room temperature. The flask was wrapped in aluminum foil in order to limit light exposure. The reaction mixture was stirred for 5 h and then poured into MeOH (70 mL) and cooled to –78 °C. The suspension was filtered, and the residue was washed with MeOH (3 x 20 mL). The solid was dissolved in CH_2Cl_2 (30 mL), washed with water (100 mL), brine (100 mL), dried ($MgSO_4$), and the solvent removed by passing N_2 over the solution. Column chromatography (silica, CH_2Cl_2/hexanes 1:2) afforded compound ***trans*-Pt** (0.083 g, 60%) as a deep blue solid. Mp 206–208 °C (decomp). R_f = 0.70 (CH_2Cl_2/hexanes 1:3). UV-vis (CH_2Cl_2) λ_{max} (ε): 259 (49600), 316 (278000), 547 (sh, 4000), 599 (sh, 9000), 654 (17500), 702 (sh, 16500). IR (ATR, solid state) 3048 (w), 2948 (m), 2863 (m), 2069 (m), 1432 (m) cm^{-1}. ^1H NMR (300 MHz, CD_2Cl_2) δ 9.09 (s, 4H), 8.54 (s, 4H), 8.08–8.02 (m, 12H), 7.93–7.90 (m, 4H), 7.62–7.60 (m, 4H), 7.39–7.34 (m, 8H), 7.12 (t, J = 7.2 Hz, 12H), 7.01 (t, J = 7.3 Hz, 6H), 2.21 (nonet, J = 6.7 Hz, 6H), 1.20 (d, J = 6.6 Hz, 36H), 0.97 (d, J = 6.9 Hz, 12H). ^{13}C NMR (100 MHz, CD_2Cl_2) δ 135.3 (pseudo-t, J_{C-P} = 6.0 Hz), 133.5, 132.5, 131.4 (pseudo-t, $^1J_{C-P}$ = 29.6 Hz), 131.3, 131.0, 130.95, 129.7, 129.4, 128.6, 128.3 (pseudo-t, J_{C-P} = 5.6 Hz), 128.1, 126.0, 125.9, 125.2, 125.1, 113.8, 112.5, 107.7, 105.8, 26.7, 25.80, 25.77 (J_{C-P} not resolved for acetylenic carbons). ^{31}P NMR (121 MHz, CD_2Cl_2) δ 18.6 (pseudo-t, J_{P-Pt} = 2603 Hz). APPI HRMS calcd for $C_{112}H_{108}P_2{}^{195}PtSi_2$ (M^+) 1765.7107, found 1765.7107. DSC: decomposition, 266 °C (onset), 403 °C (peak).

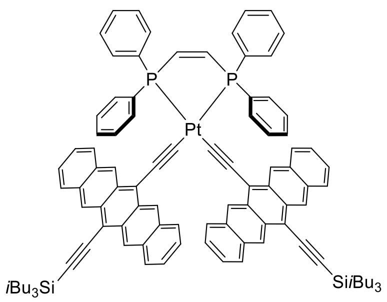

Compound ***cis*-Pt**. *cis*-1,2-(Diphenylphosphino)ethylene (0.026 g, 0.064 mmol) and ***trans*-Pt** (0.100 g, 0.056 mmol) were dissolved in dry, deoxygenated CH_2Cl_2 (20 mL). The flask was wrapped in alumina foil in order to limit light exposure. The mixture was stirred for 48 h at room temperature, and the solvent was removed by passing N_2 over the solution. Purification was achieved by column chromatography (SiO_2, hexanes/CH_2Cl_2 2:1) followed by recrystallization from CH_2Cl_2 (2 mL) and MeOH (20 mL) at 5 °C. The resulting suspension was filtered, and washed with MeOH (30 mL), affording ***cis*-Pt** (0.0734 g, 80%) as a dark blue solid. Mp 194–196 °C. R_f = 0.65 (CH_2Cl_2/hexanes 1:3). UV-vis (CH_2Cl_2) λ_{max} (ε): 273 (55500), 314 (370000), 548 (sh, 6430), 585 (sh, 12500), 632 (23000), 670 (23700). IR (ATR, solid state) 3048 (w), 2948 (m), 2864 (m), 2084 (m), 1434 (m) cm^{-1}. ^{1}H NMR (300 MHz, CD_2Cl_2) δ 9.37 (s, 4H), 9.12 (s, 4H), 8.20–8.14 (m, 8H), 7.82 (d, J = 8.7 Hz, 4H), 7.56–7.43 (m, 14H), 7.22 (t, J = 6.7 Hz, 4H), 7.14 (d, J = 8.7 Hz, 4H), 6.91 (t, J = 6.7 Hz, 4H), 2.21 (nonet, J = 6.7 Hz, 6H), 1.19 (d, J = 6.6 Hz, 36H), 0.97 (d, J = 6.9 Hz, 12H). ^{13}C NMR (100 MHz, CD_2Cl_2) δ 134.1–134.0 (m), 133.9, 132.5, 132.2, 131.7, 131.4, 130.6, 130.5, 130.0, 129.7–129.5 (m), 129.0, 128.4, 128.0, 125.9, 125.6, 125.0, 124.6, 114.4, 110.4, 108.3, 105.7, 26.7, 25.81, 25.78 ($J_{C\text{-}P}$ not resolved for acetylenic carbons). ^{31}P NMR (121 MHz, CD_2Cl_2) δ 55.1 (pseudo-t, $J_{P\text{–}Pt}$ = 2292 Hz). APPI HRMS calcd for $C_{102}H_{100}P_2{}^{195}PtSi_2$ (M^+) 1637.6481, found 1637.6497. DSC: decomposition, 265 °C (onset), 399 °C (peak).

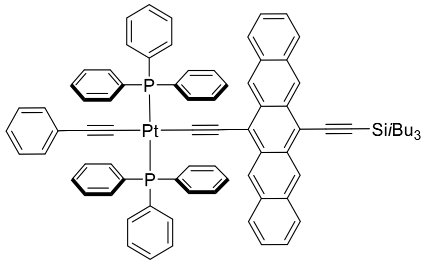

Compound **mono-Pt**: To a freshly deoxygenated solution of acetylene **1** (130mg, 0.221 mmol) in $HNEt_2$ (40 mL) were added *cis*-$Pt(PPh_3)_2Cl_2$ (175 mg, 0.221 mmol), freshly deoxygenated phenylacetylene (22.6 mg, 0.221 mmol), and CuI (8.4 mg, 0.044 mmol). The mixture was stirred for 16.5 h at 50 °C under a N_2 atmosphere. Water was added (50 mL), and the aqueous phase extracted with CH_2Cl_2 (2 x 30 mL). The combined organic phases were washed with brine (100 mL), dried ($MgSO_4$), and the solvent removed *in vacuo*. Without further purification, the product was dissolved in dry, deoxygenated THF (30 mL). To the solution were added $SnCl_2$•$2H_2O$ (130 mg, 0.576 mmol) and 10% H_2SO_4 (0.2 mL) at room temperature. The flask was wrapped in aluminum foil in order to limit light exposure. The mixture was stirred for 4 h at room temperature under a N_2 atmosphere. Water was added (60 mL), and the aqueous phase was extracted with CH_2Cl_2 (2 x 40 mL). The combined organic phases were washed with brine (100 mL) and dried ($MgSO_4$). Solvent removal and purification by column chromatography (silica gel, hexanes/DCM 3:1) afforded **mono-Pt** (105 mg, 35%) as a deep blue solid. Mp 245–248 °C. R_f = 0.54 (CH_2Cl_2/hexanes 1:1). UV-vis (CH_2Cl_2) λ_{max} (ε): 259 (57600), 269 (sh, 53400), 317 (244000), 389 (sh, 8870), 440 (2900), 507 (sh, 1960), 550 (sh, 3750), 602 (sh, 8680), 654 (14100), 702 (sh, 10800). IR (cast film, CH_2Cl_2) 3054 (w), 2952 (m), 2867 (m), 2118 (w), 2079 (m), 1503 (m), 1435 (m) cm^{-1}. ^1H NMR (500 MHz, CD_2Cl_2) δ 9.02 (s, 2H), 8.46 (s, 2H), 7.95–7.91 (m, 12H), 7.86 (d, J = 8.2 Hz 2H), 7.49 (d, J = 8.3 Hz, 2H), 7.34–7.29 (m, 4H), 7.27–7.21 (m, 18H), 6.96–6.94 (m, 3H), 6.35–6.34 (m, 2H), 2.16 (nonet, J = 6.7 Hz, 3H), 1.15 (d, J = 6.6 Hz, 18H), 0.92 (d, J = 6.9 Hz, 6H). ^{13}C NMR (126 MHz, CD_2Cl_2) δ 135.6 (pseudo-t, $J_{C–P}$ = 6.2 Hz), 132.7, 131.8 (pseudo-t, $^1J_{C–P}$ = 29.4 Hz), 131.6, 131.4, 131.2, 131.1, 129.9, 129.5, 128.9, 128.8, 128.5 (pseudo-t, $J_{C–P}$ = 5.4 Hz), 128.3, 127.8, 126.2, 125.4, 125.37, 125.2, 113.9, 113.5, 112.6, 111.5 (pseudo-t, $^1J_{C–P}$ = 29.9 Hz), 107.8, 106.1 (pseudo-t, $^1J_{C–P}$ = 27.6 Hz), 26.9, 26.0, 26.0 (two signals coincident or not observed). ^{31}P NMR (202 MHz, CD_2Cl_2) δ 18.6 (pseudo-t, $J_{P–Pt}$ = 2622 Hz). APPI HRMS calcd for $C_{82}H_{74}P_2{}^{195}PtSi_2$ ($[M + H]^+$) 1344.4761, found 1344.4734. DSC: decomposition, 264 °C (onset), 265 °C (peak).

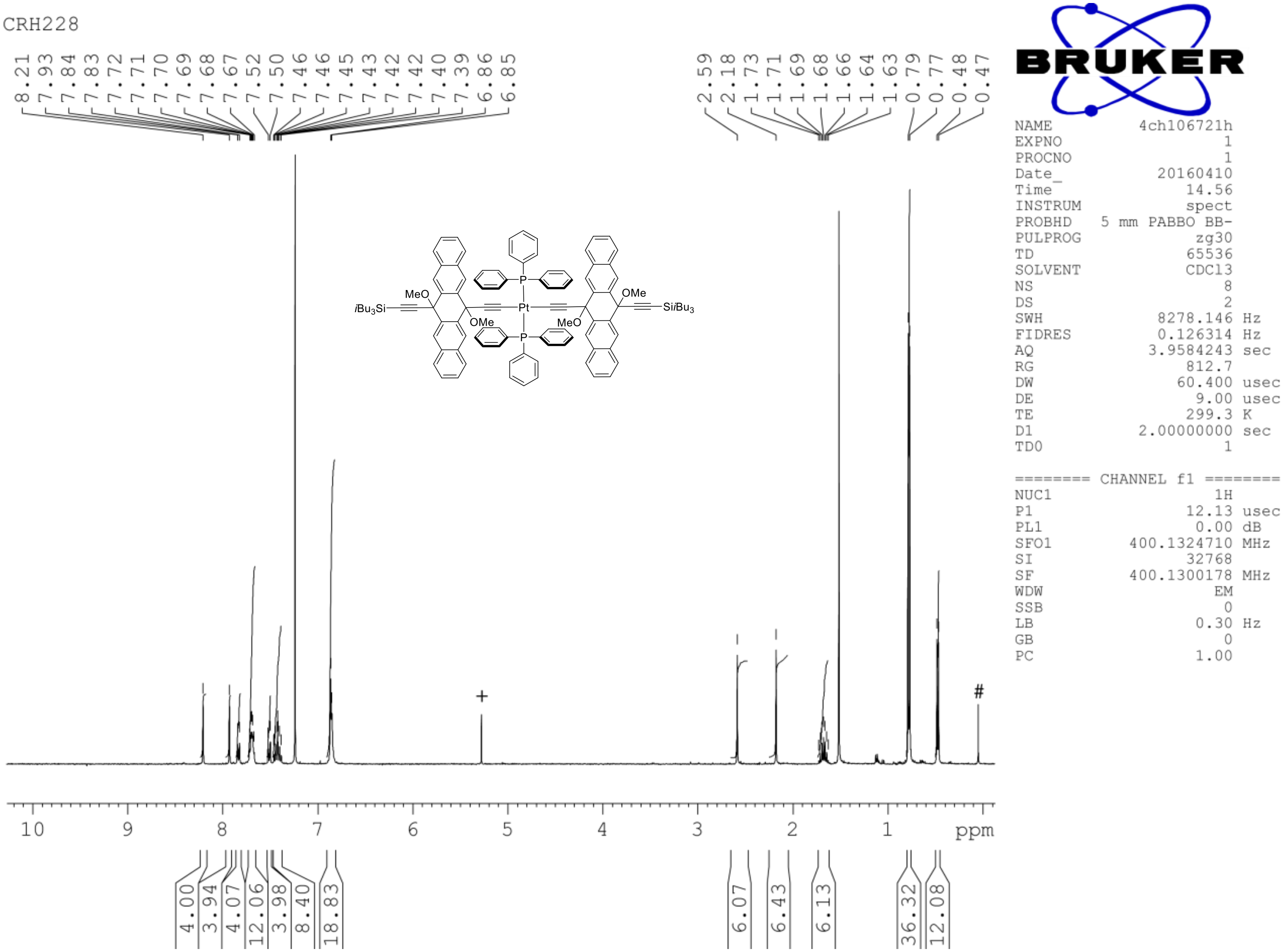

Figure S1. ^{1}H NMR spectrum of compound **2** (400 MHz, $CDCl_3$, rt, + = CH_2Cl_2, # = grease).

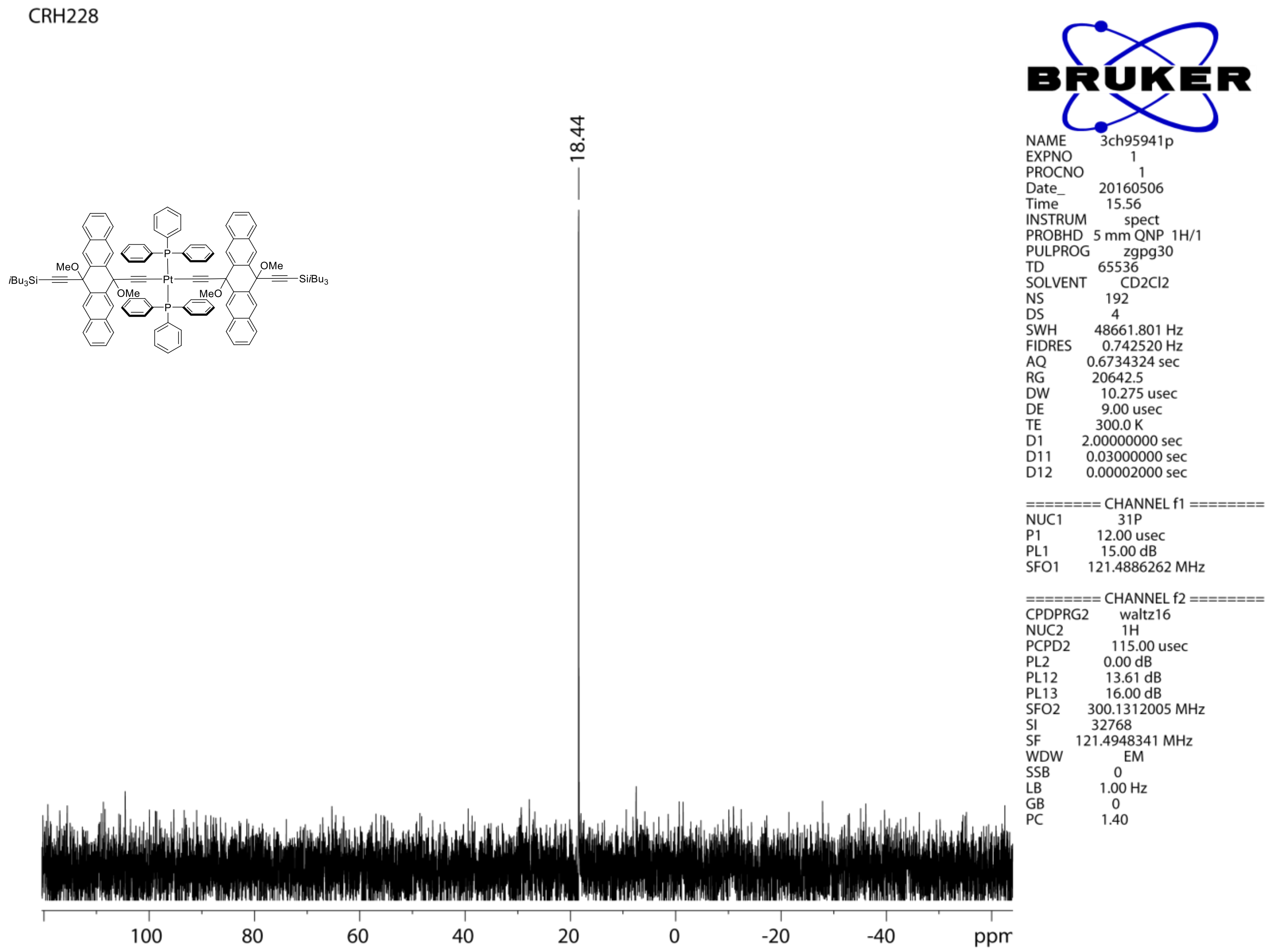

Figure S2. ^{31}P NMR spectrum of compound **2** (121 MHz, CD_2Cl_2, rt).

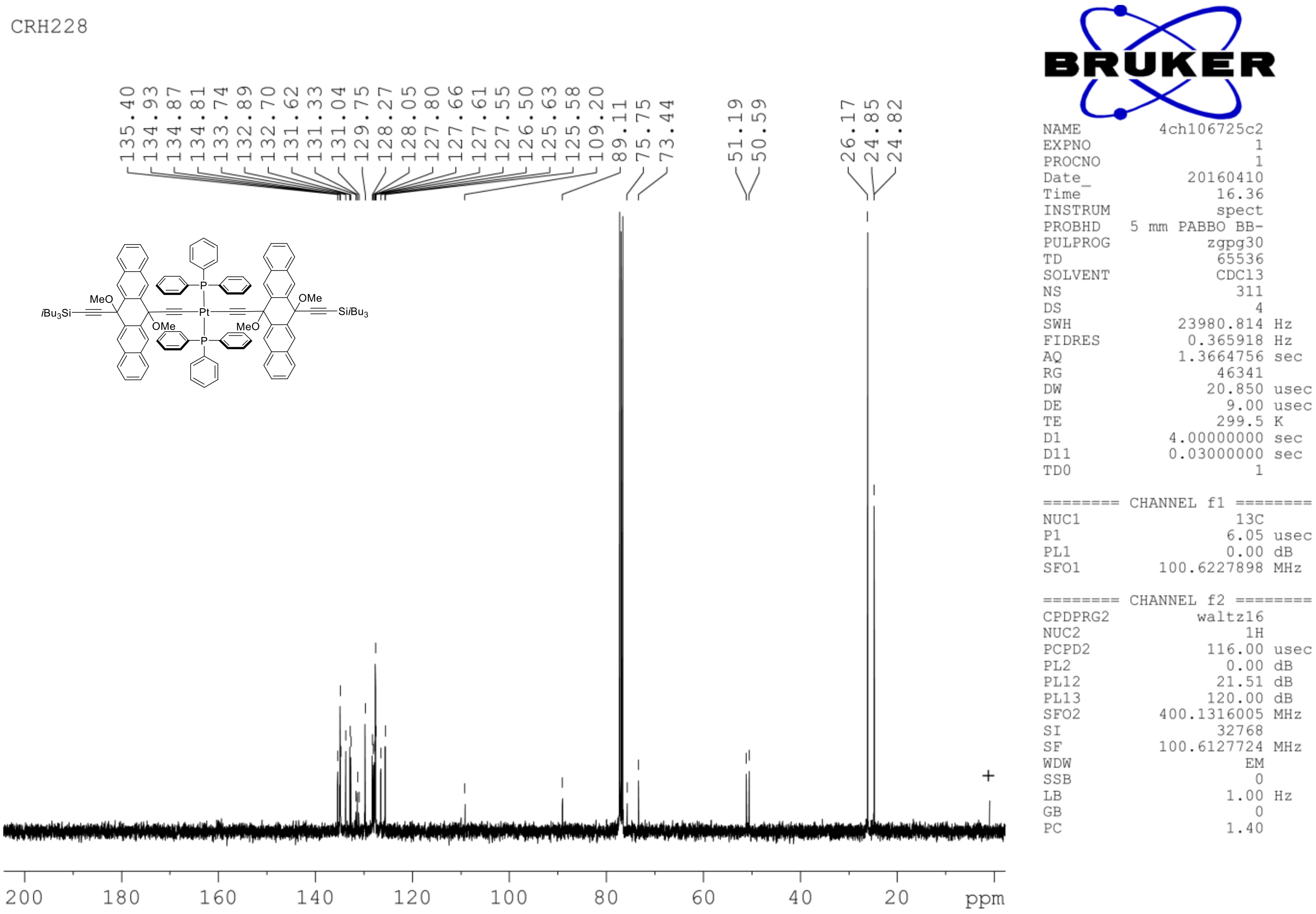

Figure S3. ^{13}C NMR spectrum of compound **2** (100 MHz, $CDCl_3$, rt, + = grease).

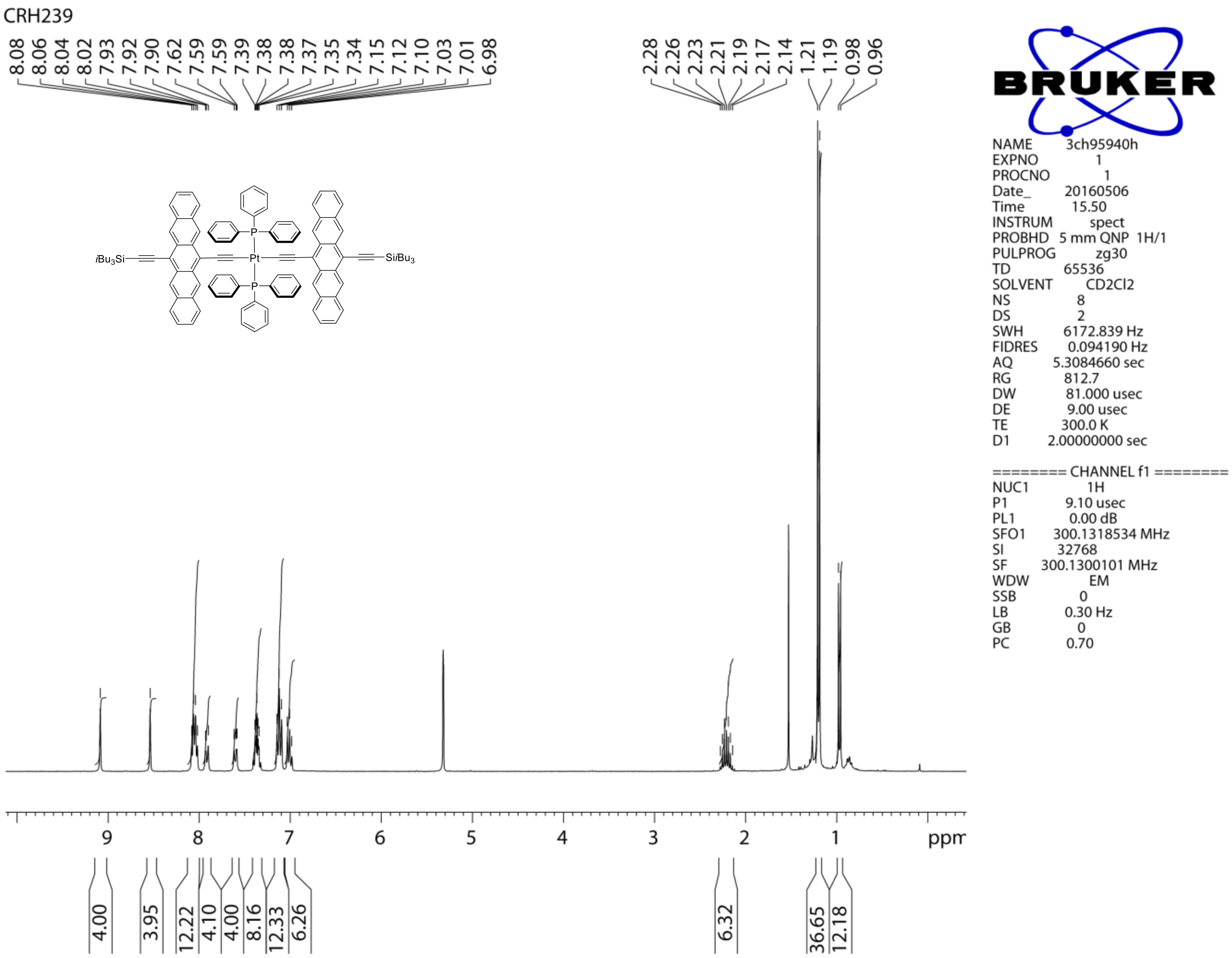

Figure S4. ^{1}H NMR spectrum of compound ***trans*-Pt** (300 MHz, CD_2Cl_2, rt).

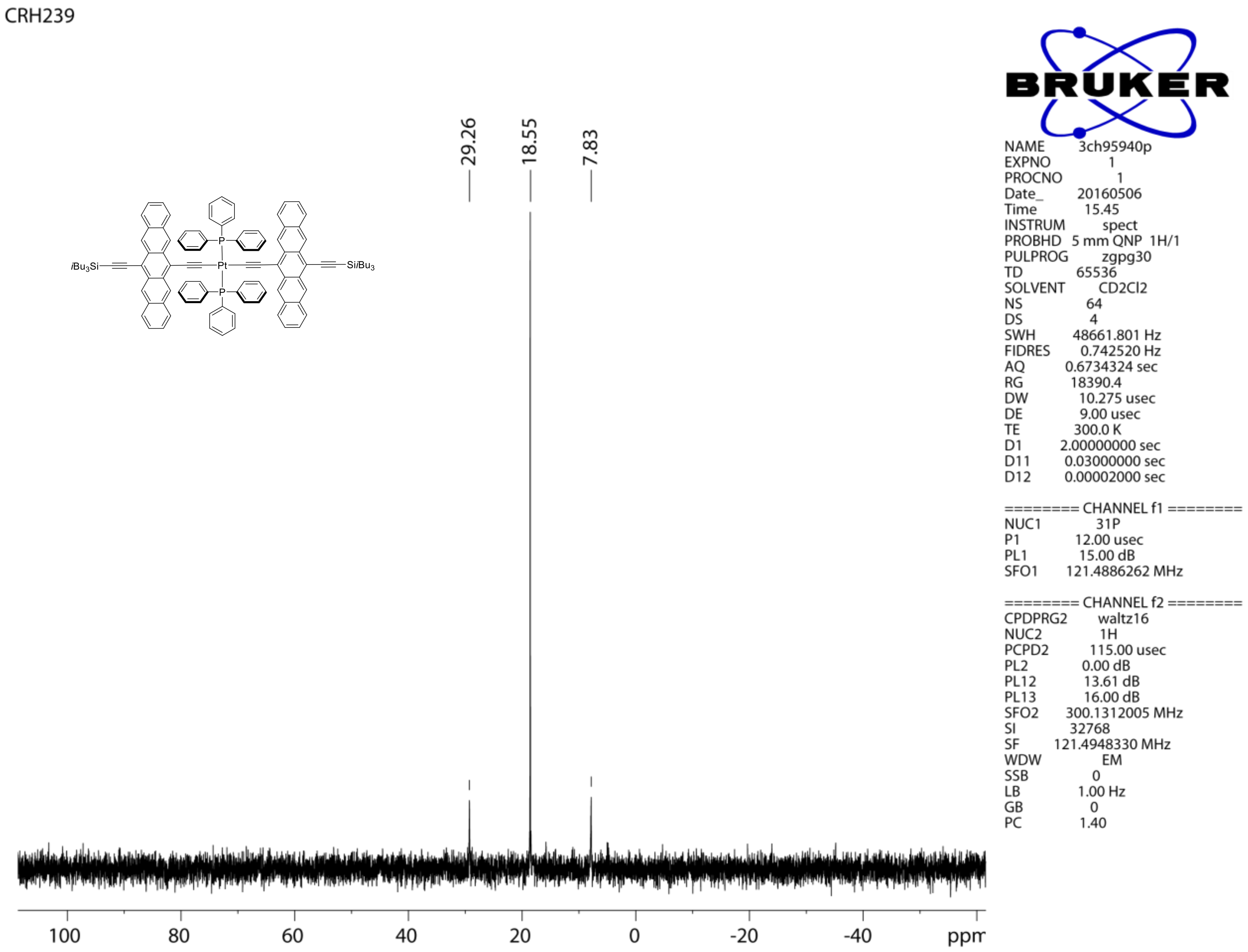

Figure S5. ^{31}P NMR spectrum of compound ***trans*-Pt** (121 MHz, CD_2Cl_2, rt).

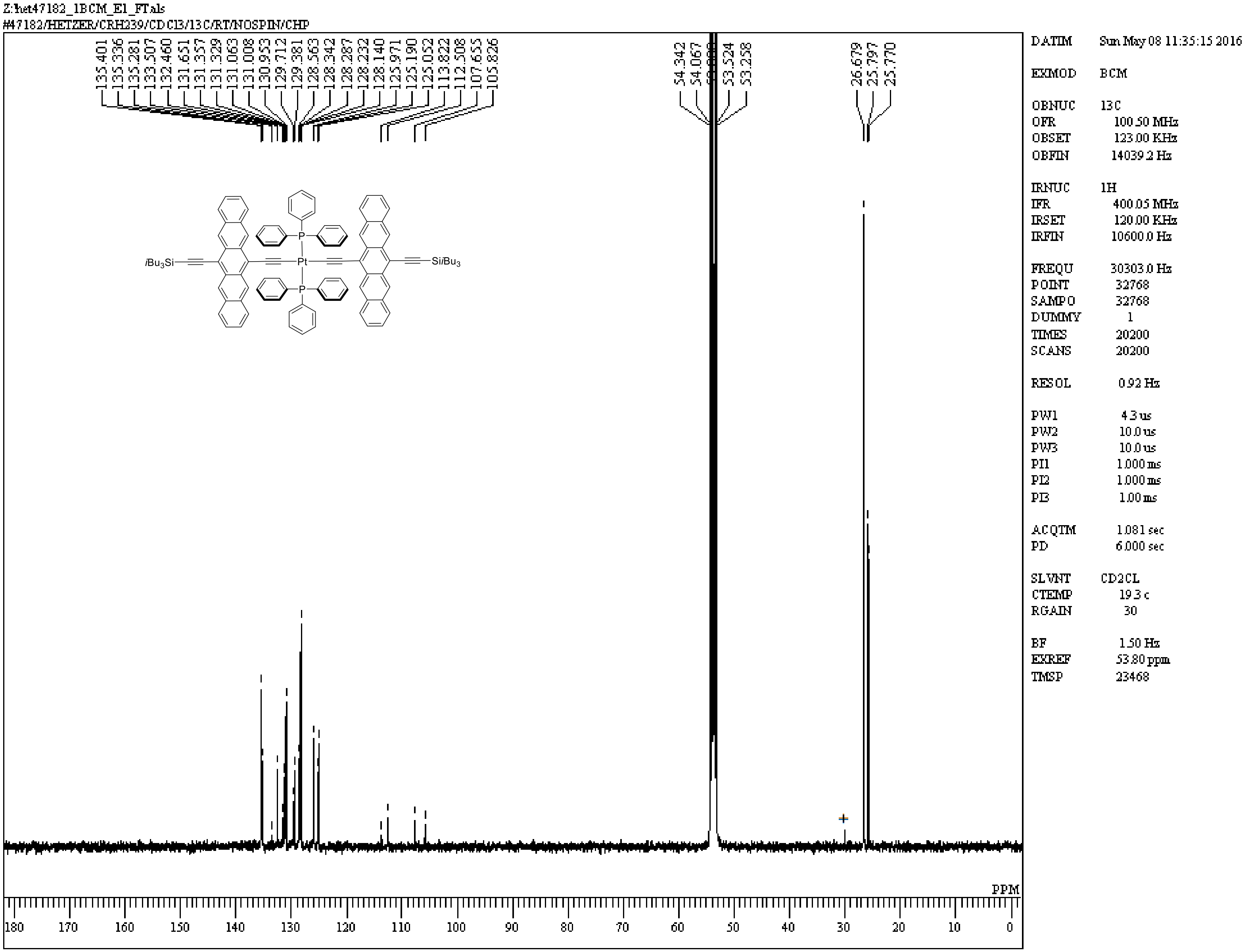

Figure S6. ^{13}C NMR spectrum of compound ***trans*-Pt** (100 MHz, CD_2Cl_2, rt, + = hexanes).

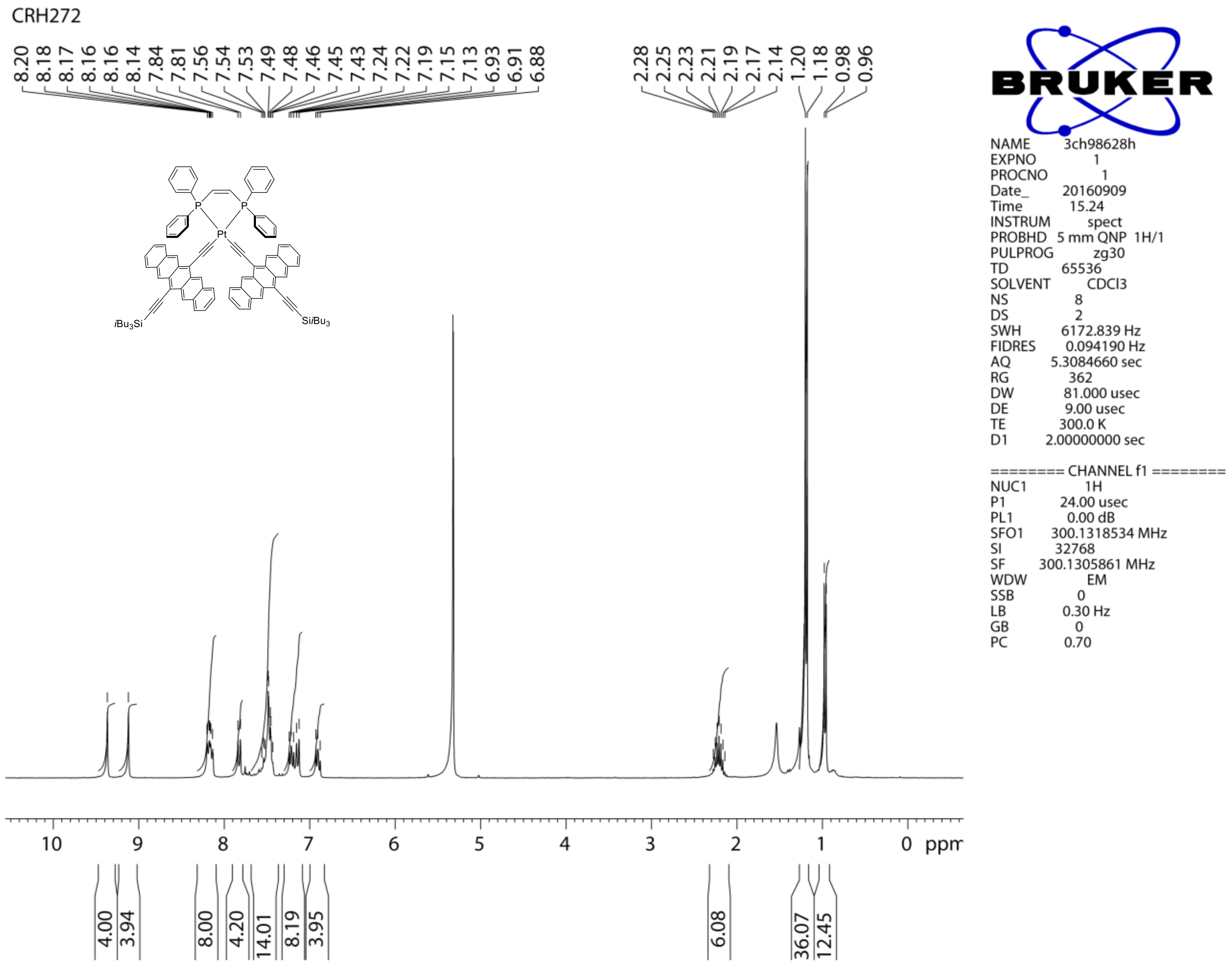

Figure S7. ^{1}H NMR spectrum of compound ***cis*-Pt** (300 MHz, CD_2Cl_2, rt).

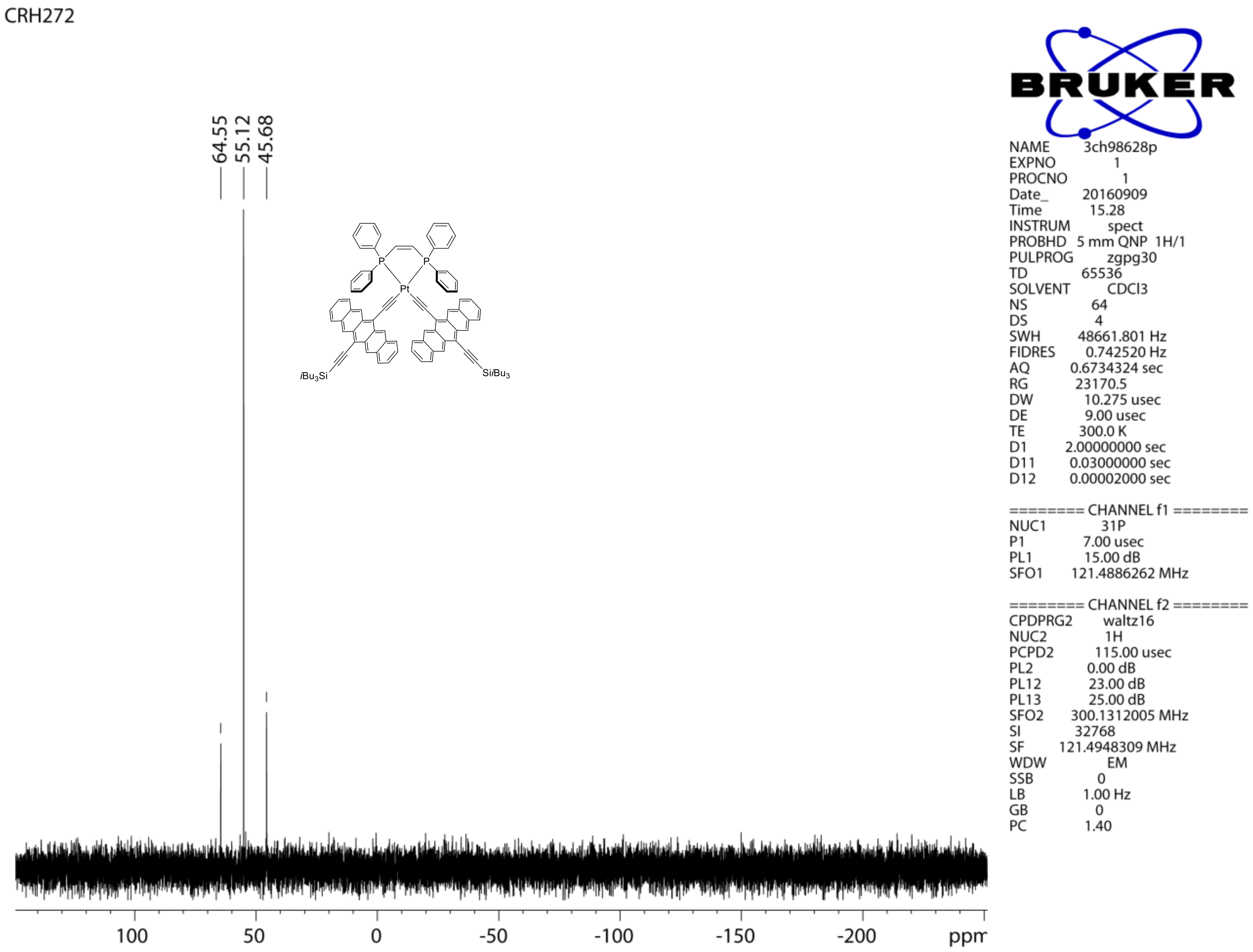

Figure S8. ^{31}P NMR spectrum of compound ***cis*-Pt** (121 MHz, CD_2Cl_2, rt).

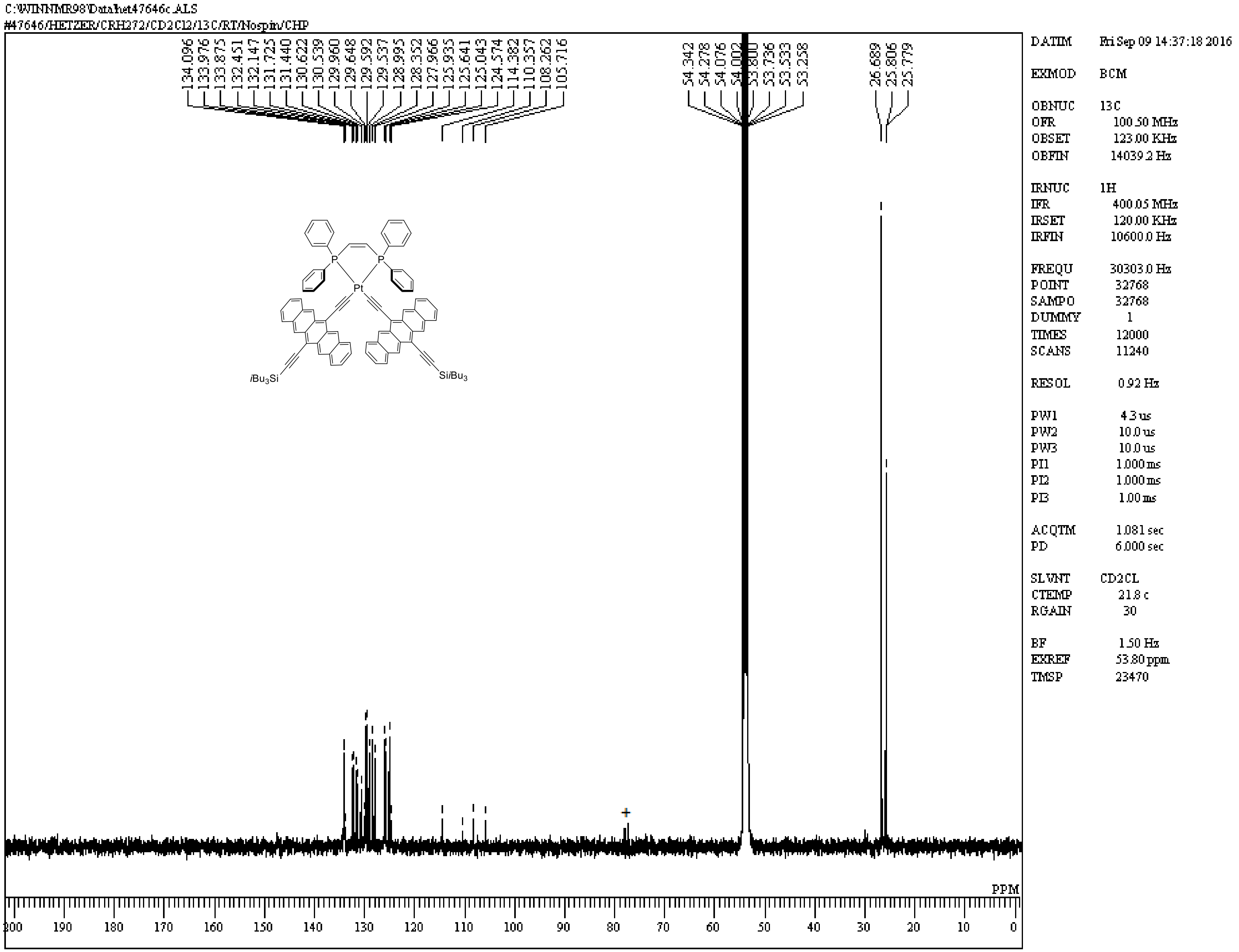

Figure S9. ^{13}C NMR spectrum of compound ***cis*-Pt** (100 MHz, CD_2Cl_2, rt, + = $CDCl_3$).

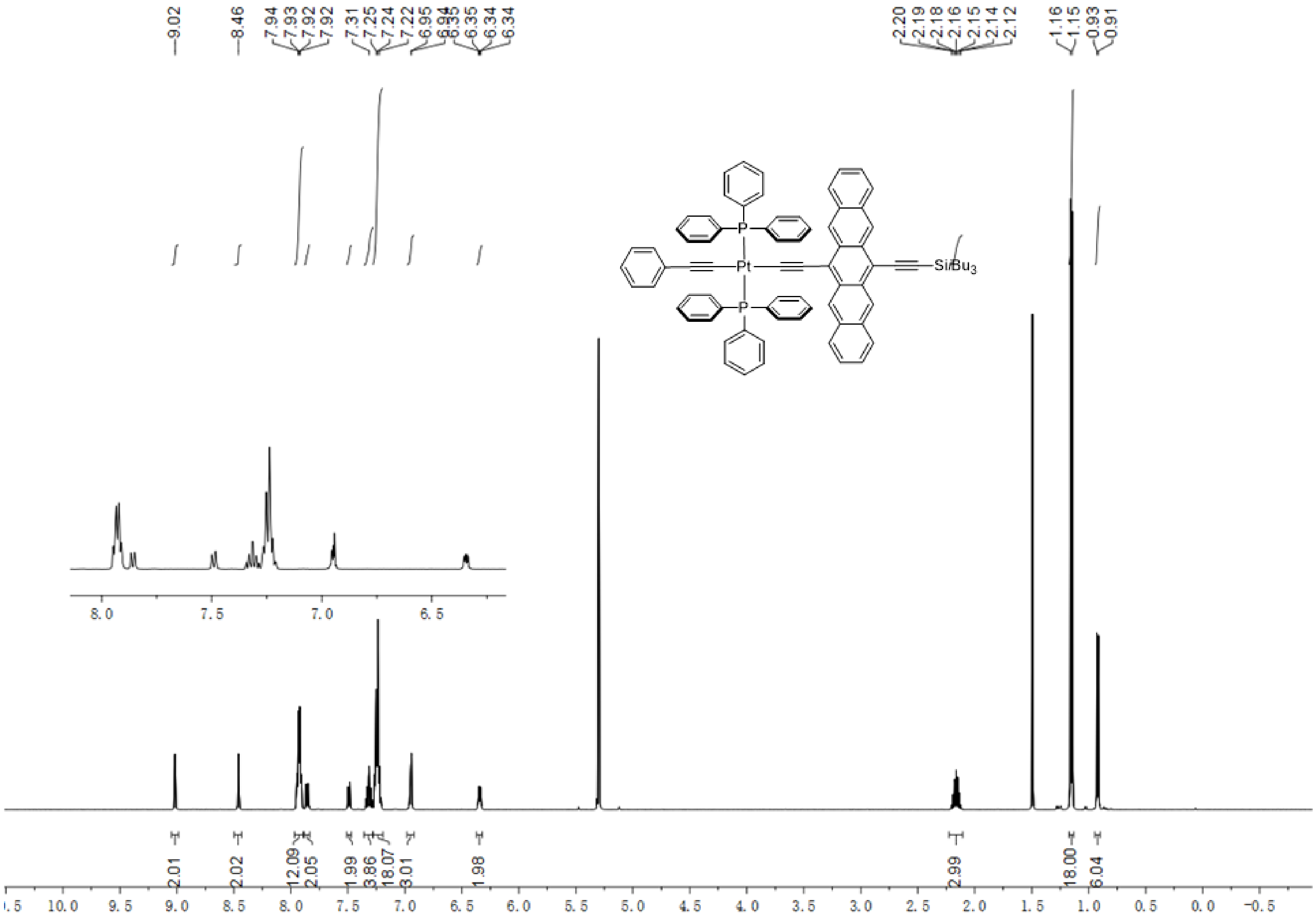

Figure S10. ^{1}H NMR spectrum of compound **mono-Pt** (500 MHz, CD_2Cl_2, rt).

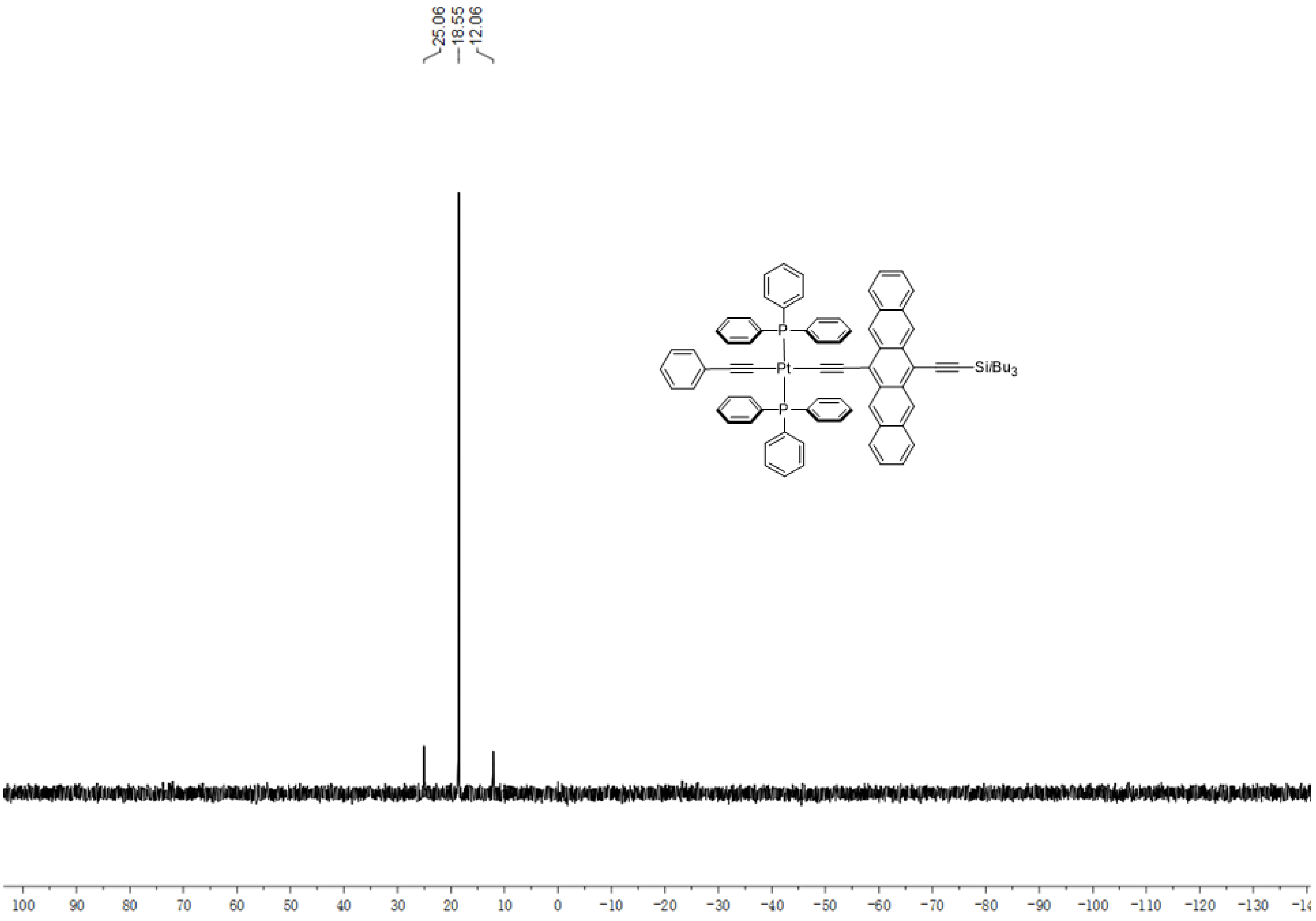

Figure S11. ^{31}P NMR spectrum of compound **mono-Pt** (202 MHz, CD_2Cl_2, rt).

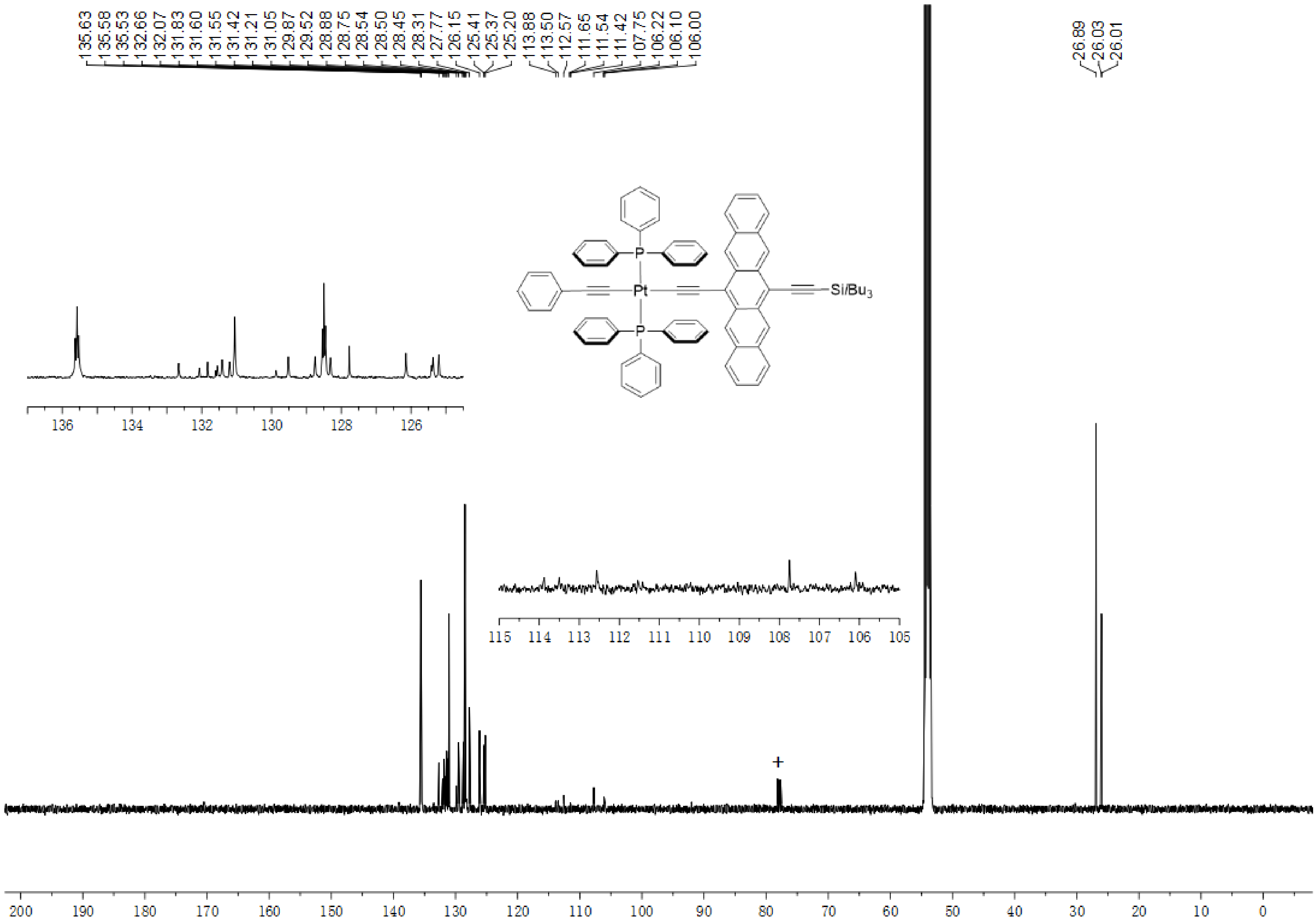

Figure S12. ^{13}C NMR spectrum of compound **mono-Pt** (126 MHz, CD_2Cl_2, rt, + = $CDCl_3$).

Supplemental photophysical characterization

Analysis methods of the transient data

Analysis of the TA data with sequential global analysis

All fs- and nsTA data were corrected for scattered light, time-zero and for probe chirp. All TA data were fit by global analysis using a sequential model.

Analysis of the fsIR data

All fsIR data were corrected for minor wavelengths shifts (~10 cm^{-1}) using the FTIR data as a reference. All fsIR data were fit by global analysis using a sequential model analogously to the analysis of the TA data.

Calculation of the triplet quantum yields

Triplet quantum yields were determined by assuming that neither equilibria nor parallel occurring transient species play a significant role for ***cis*-Pt** or ***trans*-Pt**.[19]
Following the Beer–Lambert law, the ratio of the ΔOD values in the EAS of the individual transient species at a wavelength, where the extinction coefficients are equal for the individual transient species is equal to the relative concentration ratio of the corresponding transient species.[19]
Usually, pentacene excited states (singlet and triplet) do not absorb in the transient absorption experiments, where the ground-state bleaching occurs (at around 660 nm). Therefore, the amplitude of the negative ΔOD value in the transient spectroscopy data at the ground-state bleaching is a direct measure of the amount of excited states.[20]
As the potential energy surface of the pentacene (S_1) is significantly distorted by the platinum linker in ***cis*-** and ***trans*-Pt**, it is, however, impossible to guarantee that (S_1) is non-absorbing at around 660 nm. Therefore, we performed the precise quantum yield calculations with the fsIR data instead of the TA data. The shape and the position of the ground-state bleaching of the alkyne stretching region between 2000 and 2200 cm^{-1} does not change for (S_1S_0) and (T_1T_1). Moreover, the ground-state bleaching strongly resembles the ground-state absorptions (see **Figures 5** and **S29**). This corroborates that the exited states do not possess absorptions at the ground-state bleaching in the fsIR data.

Analysis of the TA data with target analysis

We established a kinetic model for global target analysis of the fsTA data of ***trans-*** and ***cis*-Pt** and applied this to the fsTA data measured in butyronitrile at RT in the visible. We used the triplet yields calculated from the fsIR measurements in DCM. As the majority of the $^1(T_1T_1)$ states decays via the triplet channel of TTA (over $^3(T_1T_1)$) to (T_1S_0) and only a very small, undeterminable amount of (T_1+T_1) is formed, we did not implement (T_1+T_1) in the kinetic model. The lifetime of each state was first extracted from global analysis. For this purpose, the fsTA data was globally fit with 4 sequential transients: the singlet excited state (S_1S_0)* before solvent relaxation, the singlet excited state (S_1S_0) after solvent relaxation, the correlated triplet pair state with (T_1T_1) and the decorrelated triplet state (T_1S_0). As the (T_1S_0) lifetime is too long for a meaningful extraction from the fsTA data, it was extracted from a global analysis of the corresponding nsTA data. The optimized lifetimes were then used in global target analyses, while the individual rates for specific deactivation channels were allowed to vary. The fit was deemed satisfactory when the species-associated spectra of (T_1T_1) possessed about double the intensity of (T_1S_0) at the prominent pentacene triplet absorptions in the range from 450 to 525 nm (as the number of absorbing triplets is halved). It was not possible to include delayed fluorescence in this model, as the low fluorescence quantum yields of ***trans-*** and ***cis*-Pt** (<< 1%) preclude a determination of a possible rate for this process.

The fsTA data were corrected for scattered light, time-zero and for probe chirp before target analysis.

Analysis of the TREPR data

The TCW spectra were corrected for pre-time zero, DC offsets, magnetic field independent background and phase. The spectra were fit with a linear combination of spin polarized triplet spectra generated using the ‘pepper’ function in EasySpin.

Supplemental photophysical data

Supplemental steady-state characterization

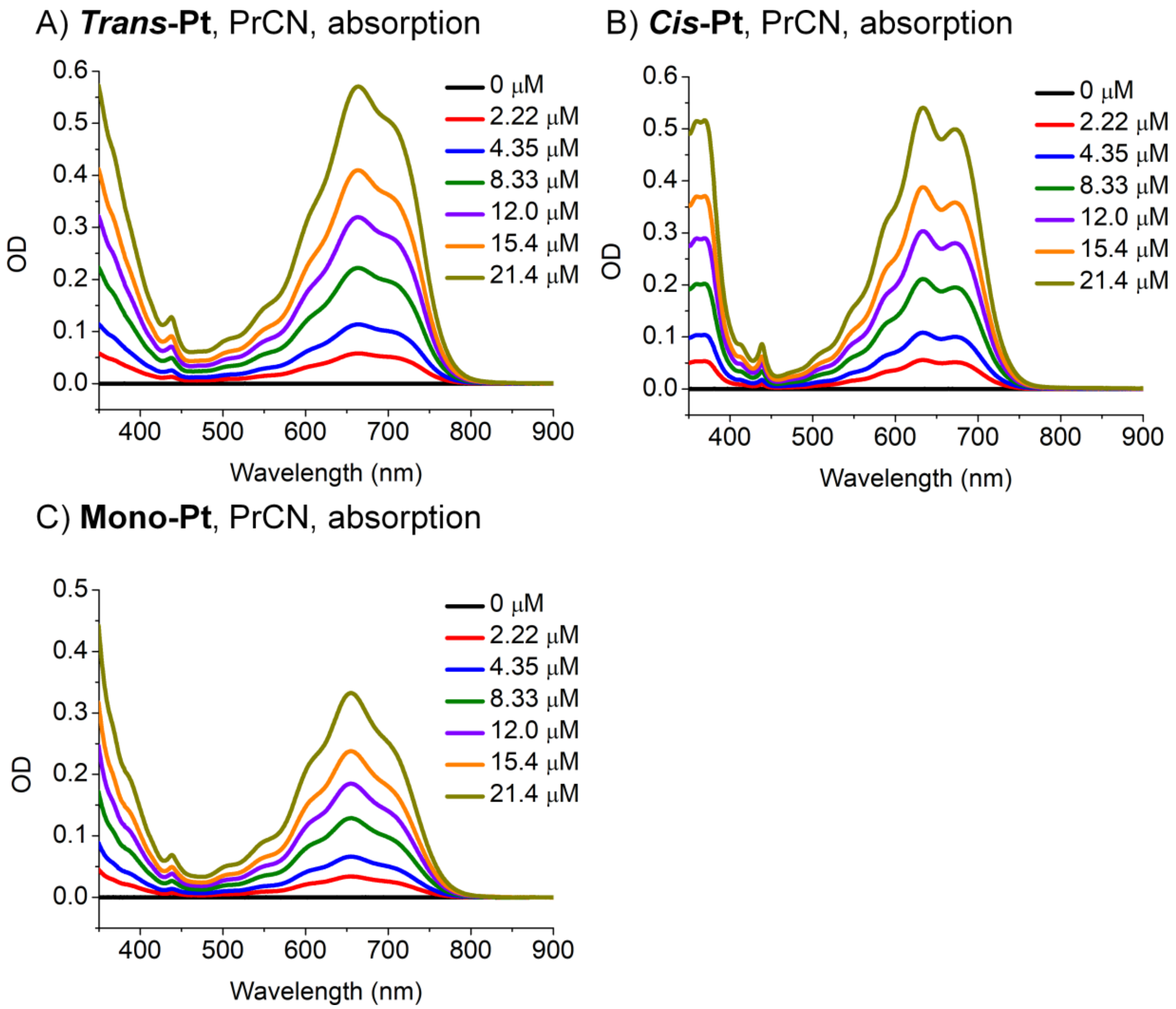

Figure S13: Concentration-dependent steady-state absorption spectra of *trans*-, *cis*-, mono-Pt. Measured in butyronitrile (PrCN) at room temperature at various concentrations.

A) ***Trans*-Pt**.

B) ***Cis*-Pt**.

C) **Mono-Pt**.

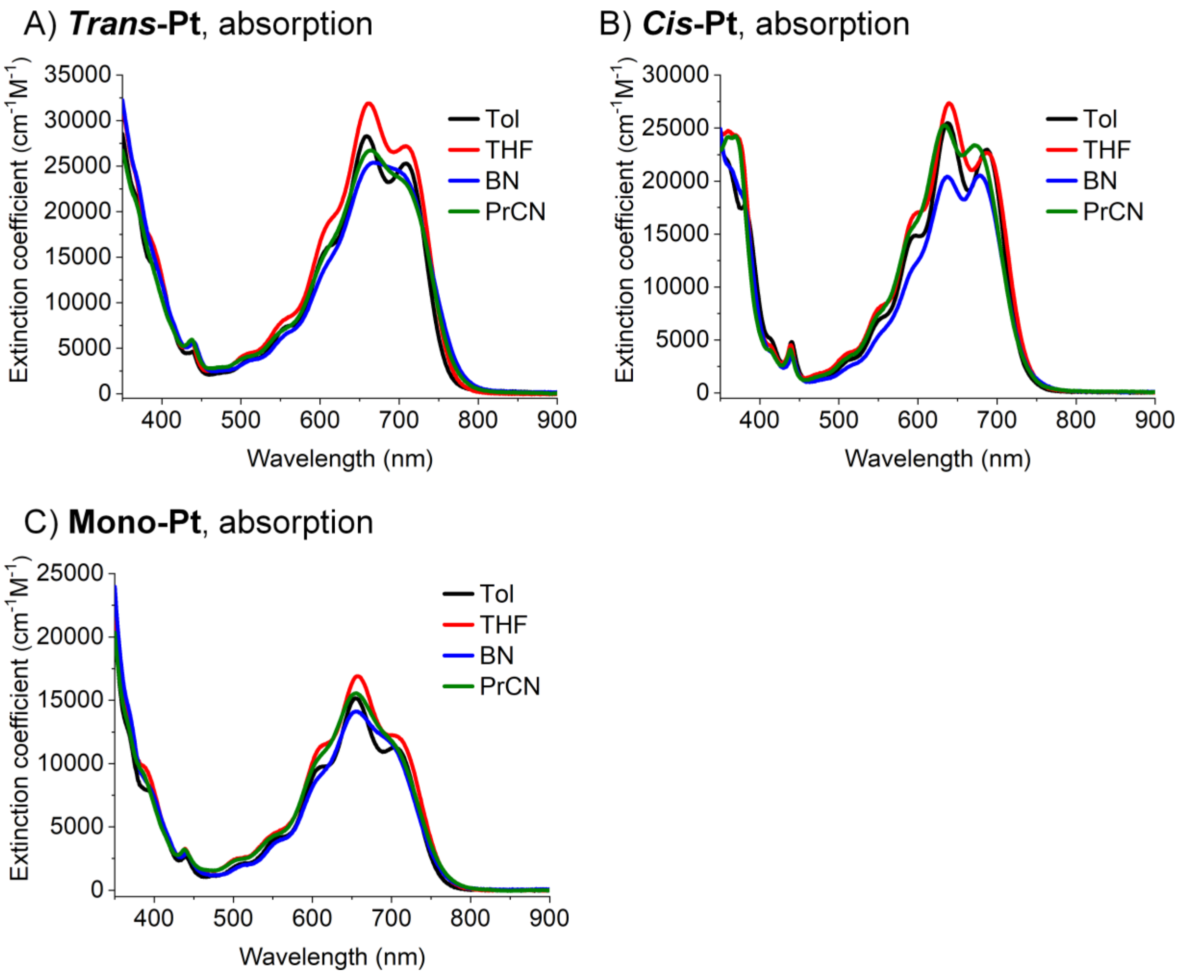

Figure S14: Polarity-dependent steady-state absorption spectra of *trans*-, *cis*-, mono-Pt. Measured at room temperature in various solvents of different polarity: toluene (Tol), THF, benzonitrile (BN) and butyronitrile (PrCN).

A) ***Trans*-Pt**.

B) ***Cis*-Pt**.

C) **Mono-Pt**.

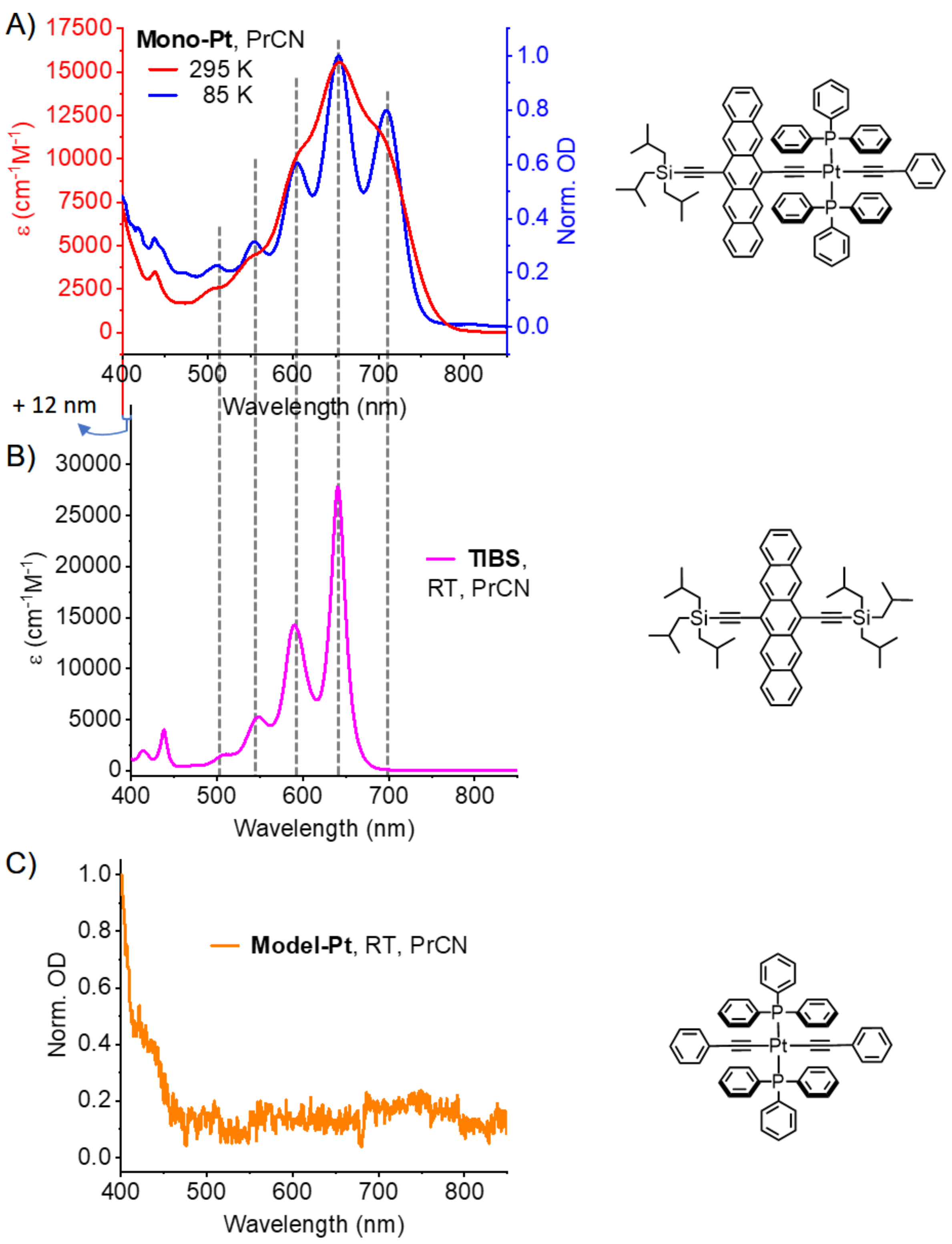

Figure S15: Comparison of the steady-state absorption spectra of mono-Pt, TIBS and model-Pt. Correlation lines are drawn between A) and B) to demonstrate the significant distortions of the pentacene potential energy surface by the platinum atom.
A) **Mono-Pt** measured at room temperature and at 85 K in butyronitrile.
B) **TIBS** measured at room temperature in butyronitrile.
C) **Model-Pt** measured at room temperature in butyronitrile.

Table S1. Vibronic maxima of the $S_1 \leftarrow S_0$ transition maximum in butyronitrile.

**Molecule**	**0-*4 transition**	**0-*3 transition**	**0-*2 transition**	**0-*1 transition**	**0-*0 transition**
**Mono-Pt**	510 nm/ 2.43 eV	553 nm/ 2.24 eV	604 nm/ 2.05 eV	652 nm/ 1.90 eV	709 nm/ 1.75 eV
***Trans*-Pt**	510 nm/ 2.43 eV	555 nm/ 2.23 eV	608 nm/ 2.04 eV	658 nm/ 1.88 eV	715 nm/ 1.73 eV
***Cis*-Pt**	510 nm/ 2.43 eV	553 nm/ 2.24 eV	592 nm/ 2.09 eV	640 nm/ 1.94 eV	694 nm/ 1.79 eV
**Molecule**	**0-*3 transition**	**0-*2 transition**	**0-*1 transition**	**0-*0 transition**	---
**TIBS**	508 nm/ 2.44 eV	548 nm/ 2.26 eV	590 nm/ 2.10 eV	641 nm/ 1.93 eV	

Table S2. Vibronic maxima of the $S_2 \leftarrow S_0$ transition maximum in butyronitrile.

**Molecule**	**0-*1 transition**	**0-*0 transition**
**Mono-Pt**	419 nm/ 2.96 eV	438 nm/ 2.83 eV
***Trans*-Pt**	413 nm/ 3.00 eV	437 nm/ 2.84 eV
***Cis*-Pt**	412 nm/ 3.01 eV	438 nm/ 2.83 eV
**TIBS**	413 nm/ 3.00 eV	438 nm/ 2.83 eV

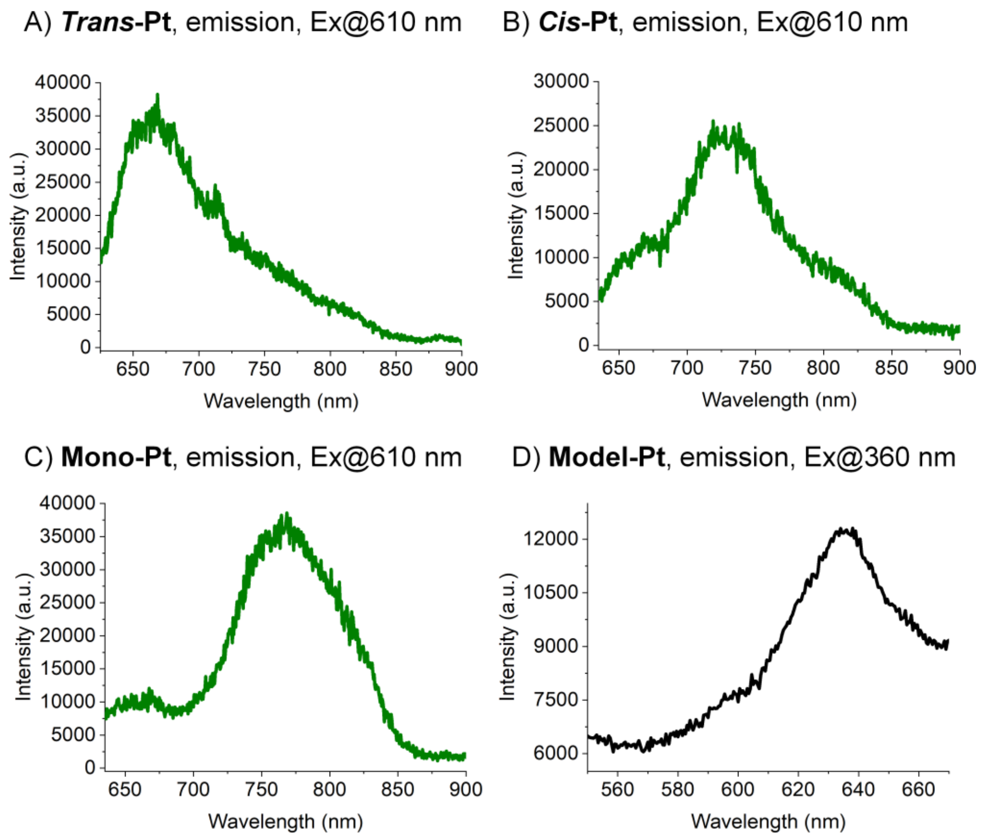

Figure S16: Steady-state emission spectra of *trans*-, *cis*-, mono- and model-Pt.
Measured in butyronitrile at room temperature.
A) ***Trans*-Pt**, excitation at 610 nm.
B) ***Cis*-Pt**, excitation at 610 nm.
C) **Mono-Pt**, excitation at 610 nm.
D) **Model-Pt**, excitation at 360 nm.

Supplemental transient absorption data in butyronitrile

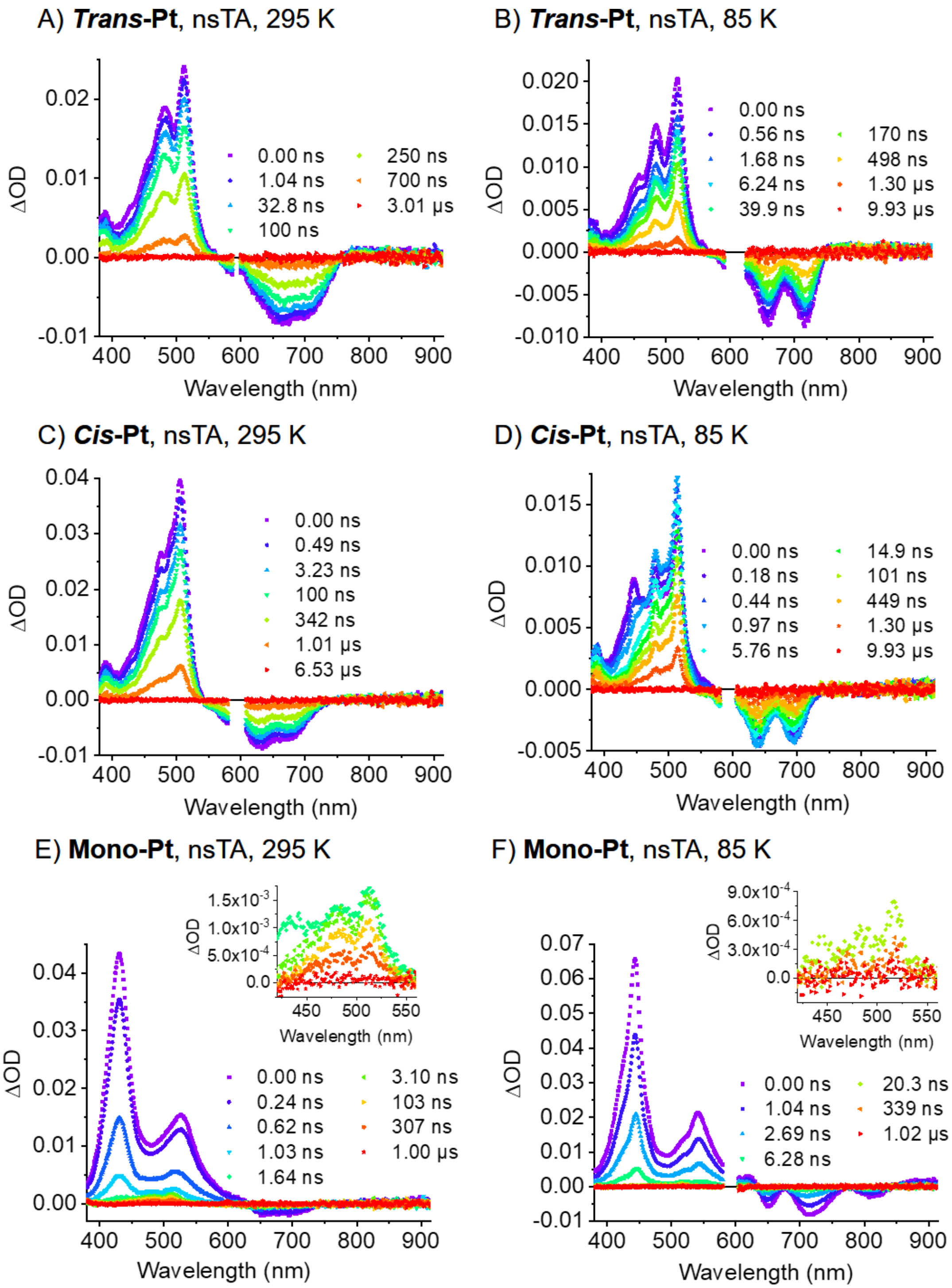

Figure S17: Nanosecond transient absorption spectra of *trans*-, *cis*- and mono-Pt
Measured at the specified temperature in oxygen-free butyronitrile.
A & B) ***Trans*-Pt** (295 K, excitation at 590 nm & 85 K, excitation at 610 nm).
C & D) ***Cis*-Pt** (295 K, excitation at 590 nm & 85 K, excitation at 590 nm).
E & F) **Mono-Pt** (295 K, excitation at 590 nm & 85 K, excitation at 590 nm).

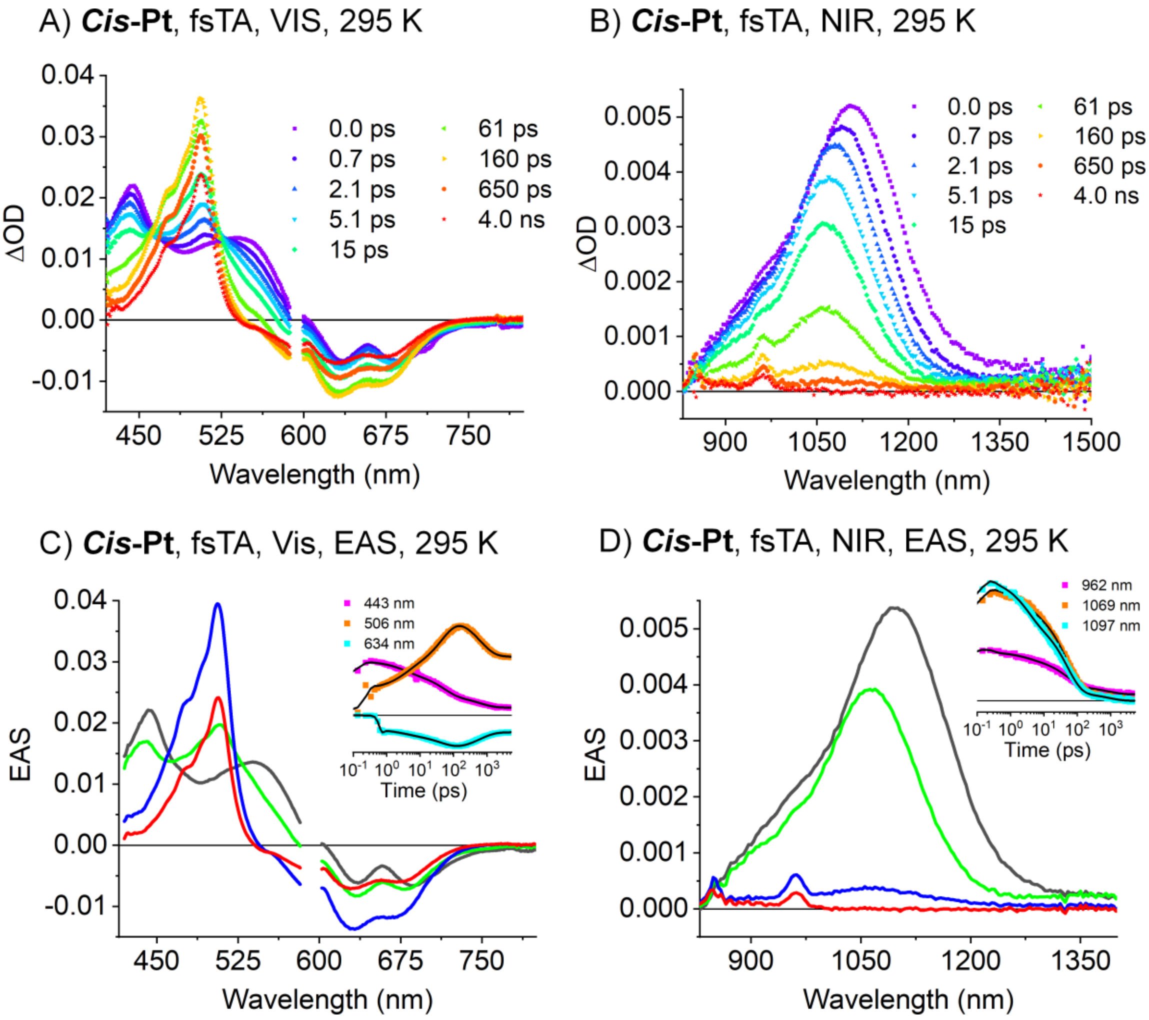

Figure S18: Femtosecond transient absorption data of *cis*-Pt
A & B) fsTA raw data of ***cis*-Pt** (VIS & NIR) measured in oxygen-free butyronitrile and at room temperature, excitation at 590 nm. C & D) EAS of the (S_0S_1)* state before solvent relaxation (gray), the (S_0S_1) state after solvent relaxation (green), the correlated triplet pair state (T_1T_1) (blue) and the decorrelated triplet state (S_0T_1) (red) of ***cis*-Pt** obtained upon global analysis with a sequential model of TA data measured in oxygen-free butyronitrile at room temperature. C) ***Cis*-Pt**, visible. See A) for the raw data D) ***Cis*-Pt**, NIR. See B) for the raw data. Insets: Raw data single-wavelength kinetics and fits to the data

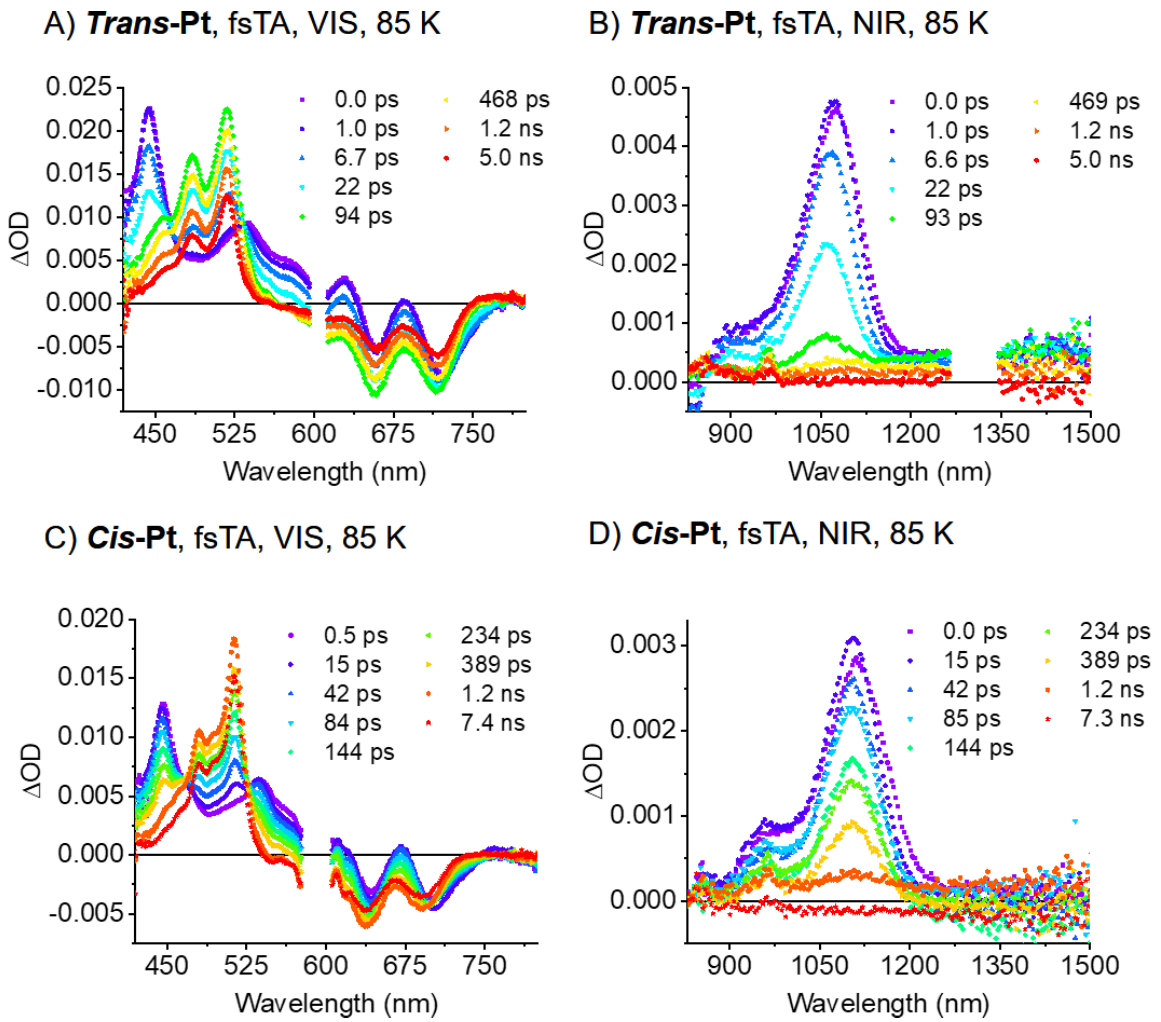

Figure S19: Femtosecond transient absorption spectra of *trans-* and *cis*-Pt at cryogenic temperatures

Measured in oxygen-free butyronitrile.

A & B) ***Trans*-Pt** (VIS & NIR) measured at 85 K, excitation at 610 nm.

C & D) ***Cis*-Pt** (VIS & NIR) measured at 85 K, excitation at 590 nm.

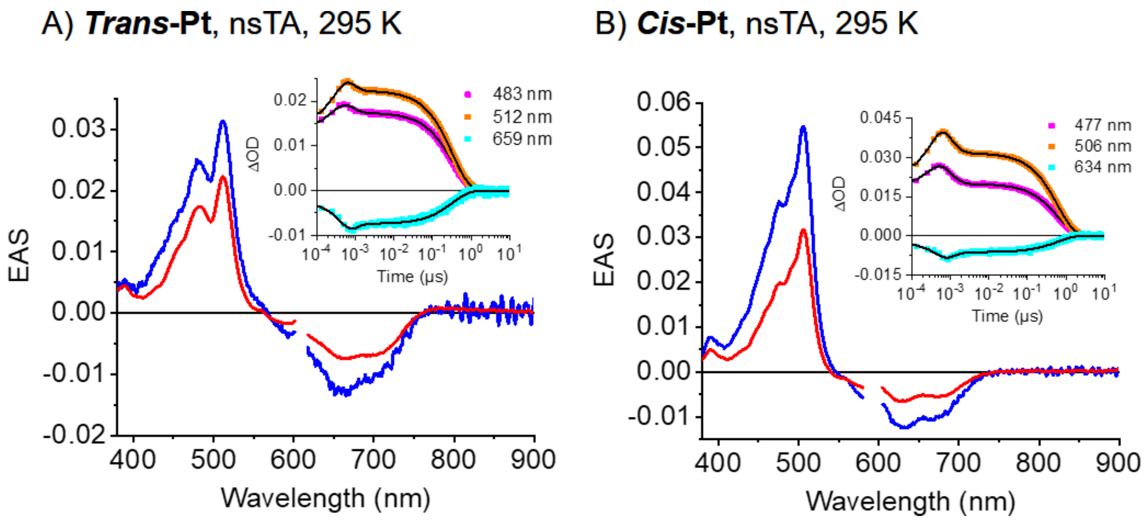

Figure S20: Nanosecond transient absorption data of *trans-* and *cis*-Pt
EAS of the correlated triplet pair state (T_1T_1) (blue) and the decorrelation triplet state $(T_1 + T_1)$ or (S_0T_1) (red) of ***trans-* and *cis*-Pt.** Obtained upon global analysis with a sequential model of nsTA data measured in oxygen-free butyronitrile at room temperature. See **Figure S17** for the raw data.
A) ***Trans*-Pt**.
B) ***Cis*-Pt**.
Insets: Raw data single wavelength kinetics and fits to the data.

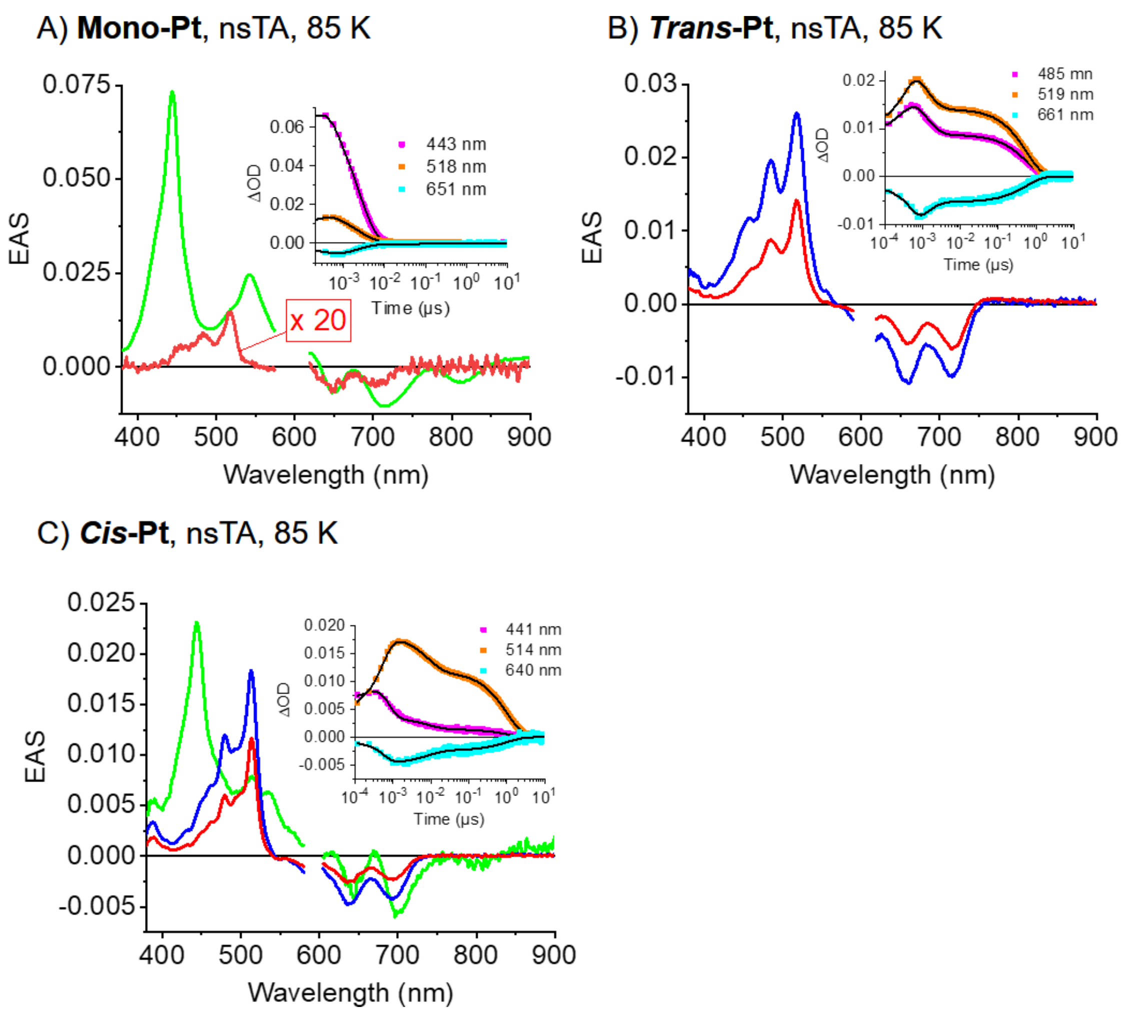

Figure S21: Nanosecond transient absorption data of mono-, *trans*-, and *cis*-Pt at cryogenic temperatures
EAS were obtained upon global analysis with a sequential model of nsTA data measured in oxygen-free butyronitrile at 85 K. See **Figure S17** for the raw data.
A) EAS of the singlet state S_1 after relaxation (green) and the triplet state T_1 (red) of **mono-Pt.**
B) EAS of the (S_0S_1) state after relaxation (green), the correlated triplet pair state (T_1T_1) (blue) and the decorrelation triplet state (T_1 + T_1) or (S_0T_1) (red) of ***trans*-Pt**.
C) EAS of the (S_0S_1) state after relaxation (green), the correlated triplet pair state (T_1T_1) (blue) and the decorrelation triplet state (T_1 + T_1) or (S_0T_1) (red) of ***cis*-Pt**.
Insets: Raw data single wavelength kinetics and fits to the data.

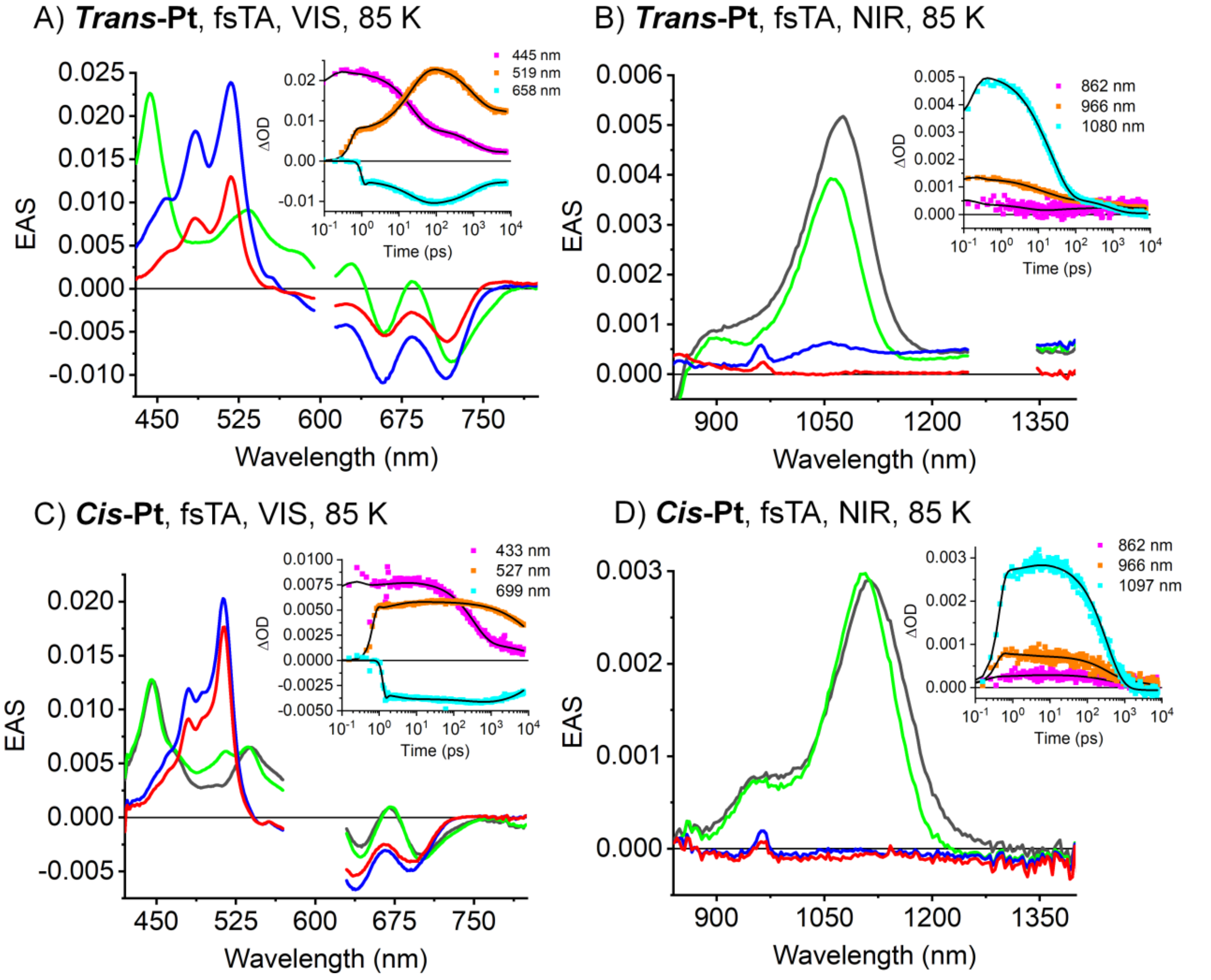

Figure S22: Femtosecond transient absorption data of *trans-*, *cis*-Pt at cryogenic temperatures
EAS were obtained upon global analysis with a sequential model of fsTA data measured in oxygen-free butyronitrile at 85 K. See **Figure S19** for the raw data.
A) EAS of the (S_0S_1) state after relaxation (green), the correlated triplet pair state (T_1T_1) (blue) and the decorrelation triplet state ($T_1 + T_1$) or (S_0T_1) (red) of ***trans*-Pt**.
B) EAS of the (S_0S_1)* state before relaxation (grey), EAS of the (S_0S_1) state after relaxation (green), the correlated triplet pair state (T_1T_1) (blue) and the decorrelation triplet state ($T_1 + T_1$) or (S_0T_1) (red) of ***trans*-Pt**.
C and D) EAS of the (S_0S_1)* state before relaxation (grey), EAS of the (S_0S_1) state after relaxation (green), the correlated triplet pair state (T_1T_1) (blue) and the decorrelation triplet state ($T_1 + T_1$) or (S_0T_1) (red) of ***cis*-Pt**.
Insets: Raw data single wavelength kinetics and fits to the data.

Table S3. Lifetimes of the transient states of mono-, *cis*- and *trans*-Pt in butyronitrile at 85 K.[a, b]

**Solvent**	**Mono-Pt**	***Cis*-Pt**	***Trans*-Pt**
(S_1S_0)	---	0.33 ± 0.02 ns	22 ± 1 ps
S_1	2.34 ± 0.12 ns	---	---
(T_1T_1)	---	8.52 ± 0.43 ns	1.11 ± 0.06 ns
(T_1S_0) / $(T_1 + T_1)$	---	1.02 ± 0.05 µs	556 ± 28 ns
T_1	370 ± 19 ns	---	---

[a] See **Figures S21 and S22** for the global fits and **Figures S17 and S19** for the raw data.

Global target analysis

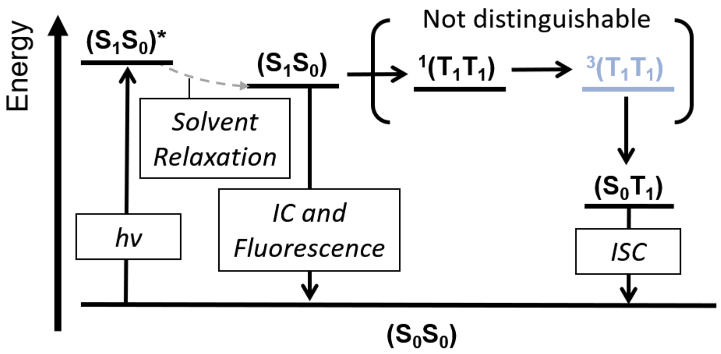

Figure S23. Kinetic model used to fit the fsTA data of ***cis*-** and ***trans*-Pt** in butyronitrile (PrCN) at RT via target analysis. As it is was not possible to distinguish $^1(T_1T_1)$ and $^3(T_1T_1)$ in the TA data (see manuscript), only one correlated triplet pair species (T_1T_1) could be implemented in the target analysis.

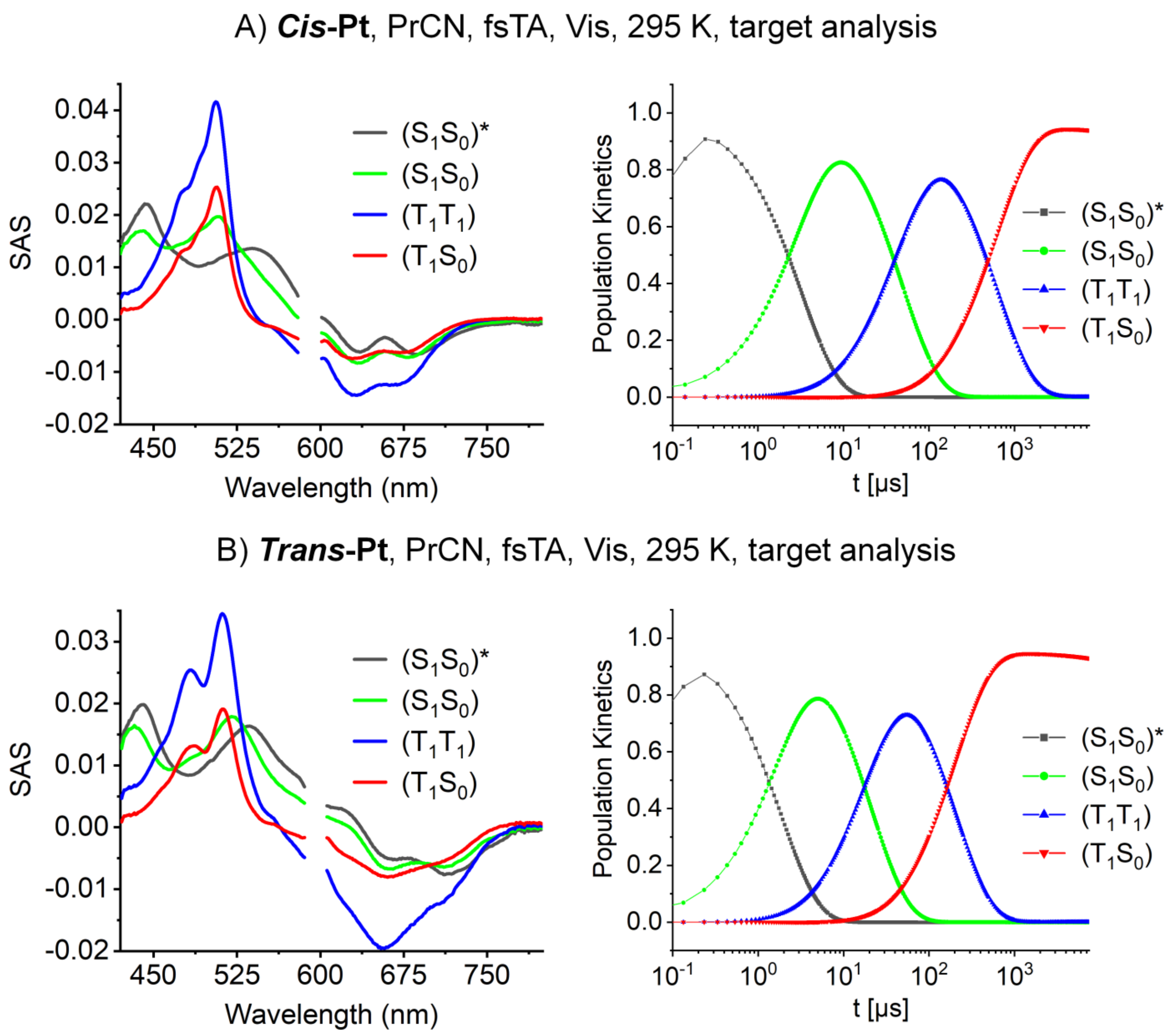

Figure S24. Species-associated spectra (SAS) and the corresponding population kinetics of the fsTA data of ***cis-*** and ***trans*-Pt** in oxygen-free butyronitrile (PrCN) as obtained by target analysis.
See **Figure S23** for the kinetic model and **Table S4** for the used rate constants. The linear combination of each SAS on the left and the corresponding population kinetics on the right yields the complete 3-D TA data set (ΔOD versus time and wavelength).
A) ***Cis*-Pt**, see **Figure S18** for the raw data
B) ***Trans*-Pt**, see **Figure 4** for the raw data.

Table S4. Rate constants and quantum yields for ***cis-*** and ***trans*-Pt** using the kinetic model shown in **Figure S23**.[a]

**Initial State**	**Resulting State**	**Rate Constant / Quantum Yield**[b]	
		***Cis*-Pt**	***Trans*-Pt**
$(S_1S_0)^*$	(S_1S_0)	3.10 x 10^{11} s^{-1} / 100%	5.26 x 10^{11} s^{-1} / 100%
	(S_0S_0)	0 s^{-1} / 0%	0 s^{-1} / 0%
(S_1S_0)	(T_1T_1)	1.94 x 10^{10} s^{-1} / 95%[c]	4.55 x 10^{10} s^{-1} / 95%[c]
	(S_0S_0)	1.02 x 10^{9} s^{-1} / 5%	2.40 x 10^{9} s^{-1} / 5%
(T_1T_1)	(T_1S_0)	1.60 x 10^{9} s^{-1} / 100 %	5.01 x 10^{9} s^{-1} / 100 %
	(S_0S_0)	0 s^{-1} / 0%	0 s^{-1} / 0%
(T_1S_0)	(S_0S_0)	1.64 x 10^{6} s^{-1} / 100%	3.03 x 10^{6} s^{-1} / 100%

[a] See **Figure 24** for the corresponding target analyses.

[b] The quantum yield is always given with respect to the initial state on the left side of the corresponding row. It is calculated from the target analysis as the ratio of the corresponding rate and the sum over all rates that lead to a deactivation of the corresponding initial state.

[c] Please note: a quantum yield of 95% for the transition from (S_1S_0) to (T_1T_1) corresponds to a triplet yield of 2×95% = 190%, as (T_1T_1) consists of two correlated triplets.

Supplemental polarity-dependent characterization

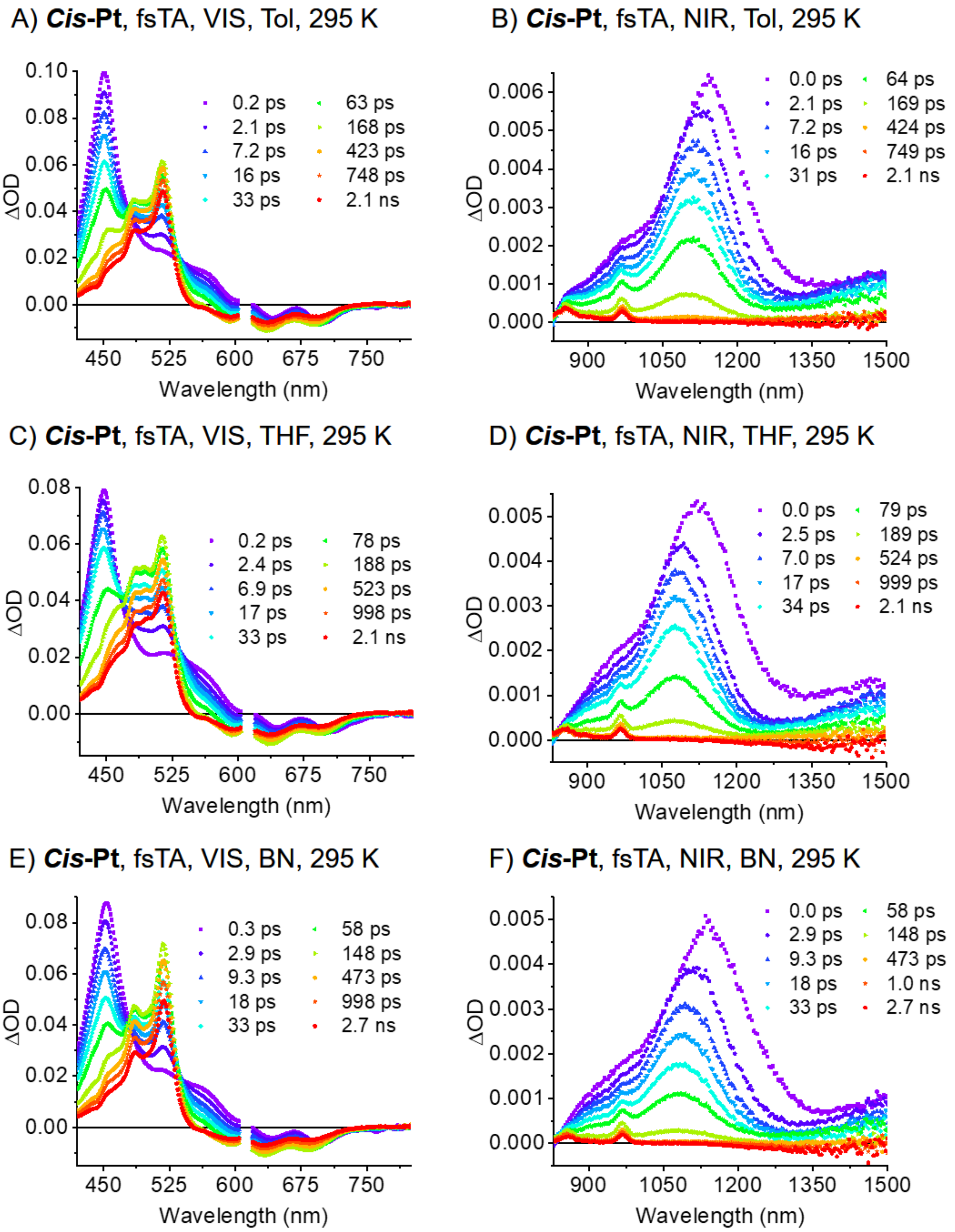

Figure S25: Femtosecond transient absorption spectra of *cis*-Pt in various solvents
Measured at room temperature with an excitation wavelength of 610 nm.
A & B) ***Cis*-Pt** (VIS & NIR) measured in argon saturated toluene.
C & D) ***Cis*-Pt** (VIS & NIR) measured in argon saturated THF.
E & F) ***Cis*-Pt** (VIS & NIR) measured in argon saturated benzonitrile.

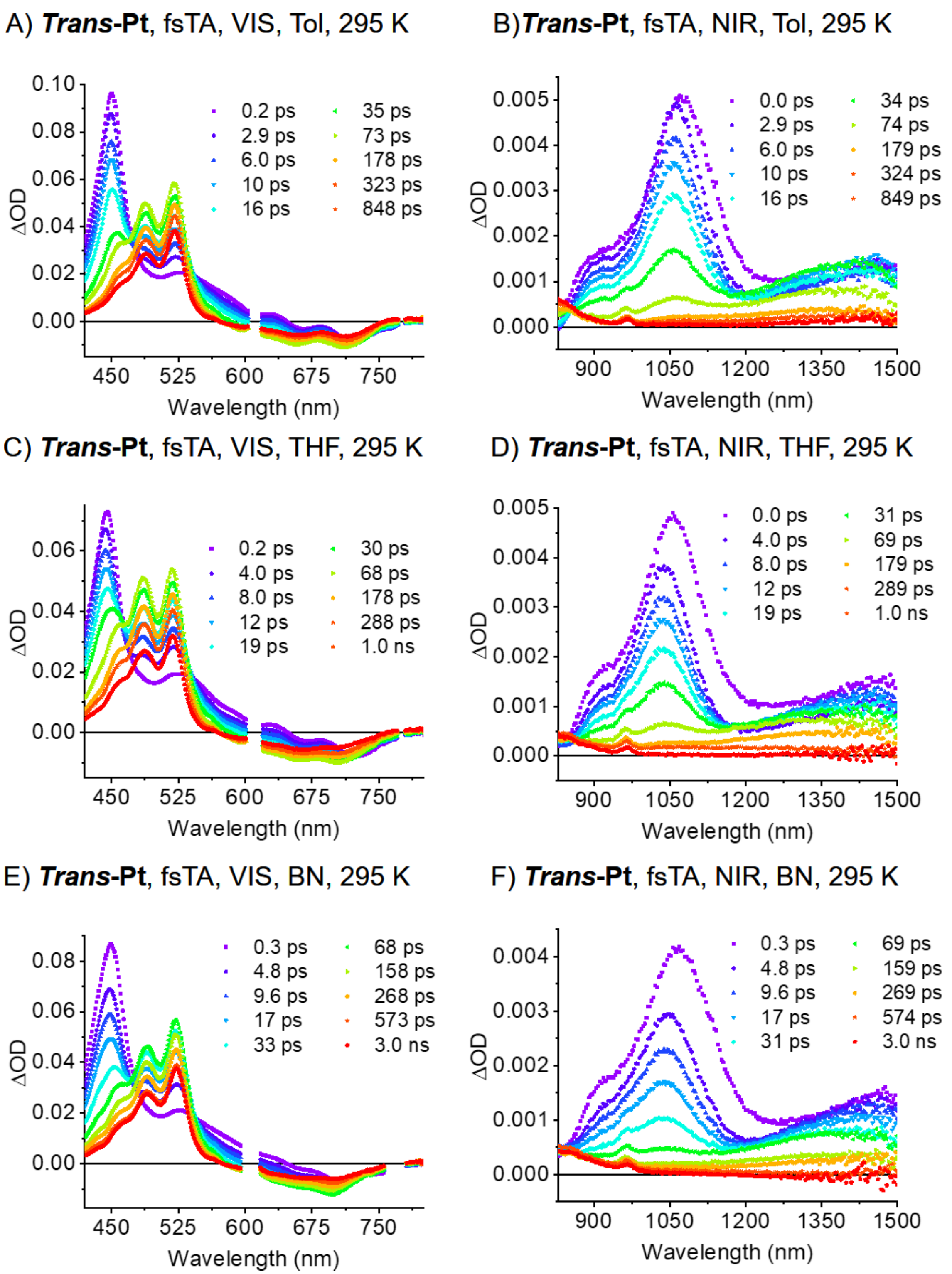

Figure S26: Femtosecond transient absorption spectra of *trans*-Pt in various solvents
Measured at room temperature with an excitation wavelength of 610 nm.
A & B) ***Trans*-Pt** (VIS & NIR) measured in argon saturated toluene.
C & D) ***Trans*-Pt** (VIS & NIR) measured in argon saturated THF.
E & F) ***Trans*-Pt** (VIS & NIR) measured in argon saturated benzonitrile.

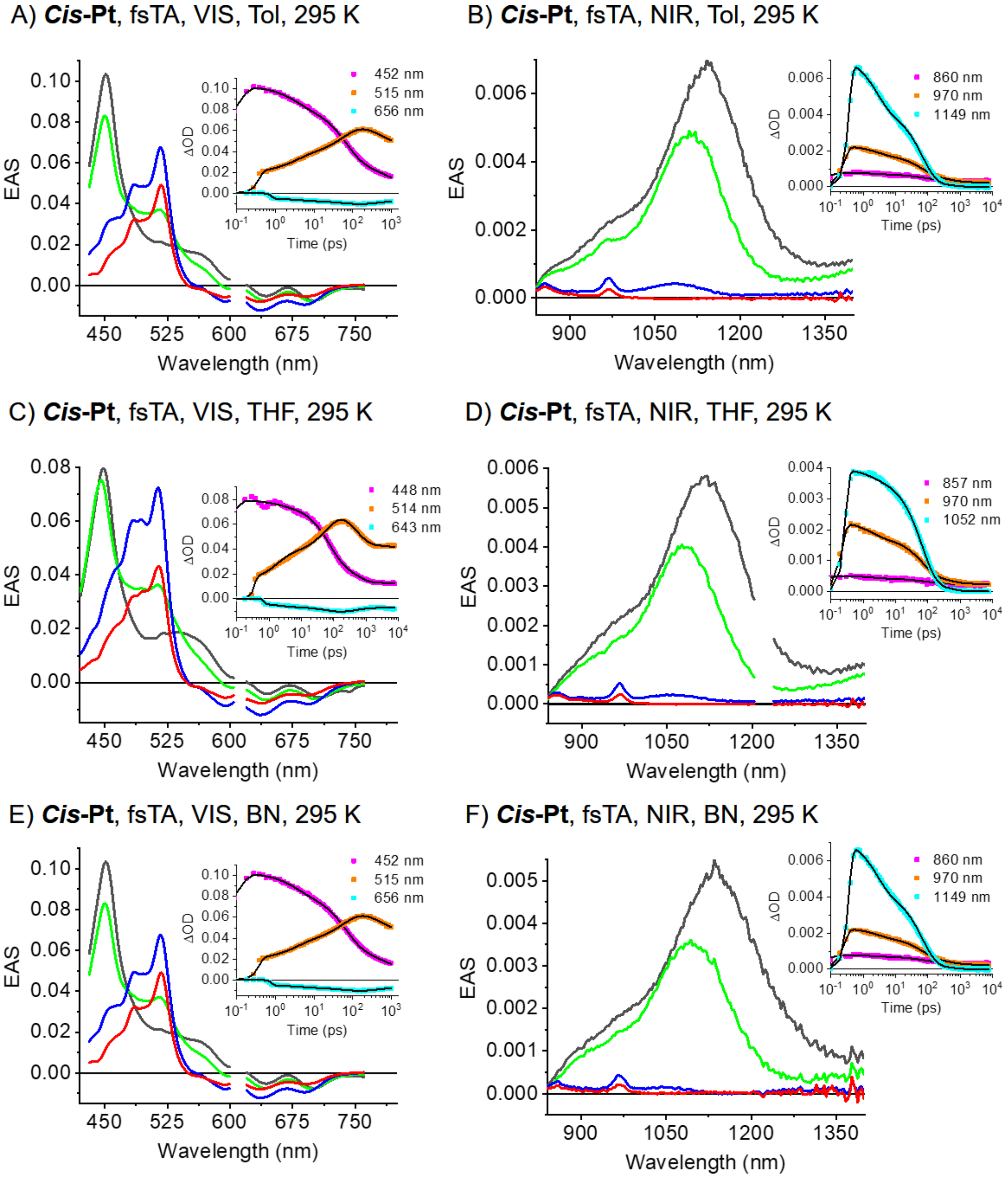

Figure S27: Transient absorption data of *cis*-Pt in various solvents

All figures show the EAS of the $(S_0S_1)^*$ state before solvent relaxation (grey), the (S_0S_1) state after solvent relaxation (green), the correlated triplet pair state (T_1T_1) (blue) and the decorrelation triplet state $(T_1 + T_1)$ or (S_0T_1) (red) of ***cis*-Pt**. The EAS were obtained upon global analysis with a sequential model of fsTA data measured at room temperature. See **Figure S23** for the raw data.

A & B) EAS of ***cis*-Pt** (VIS & NIR) measured in argon saturated toluene.

C & D) EAS of ***cis*-Pt** (VIS & NIR) measured in argon saturated THF.

E & F) EAS of ***cis*-Pt** (VIS & NIR) measured in argon saturated benzonitrile.

Insets: Raw data single wavelength kinetics and fits to the data.

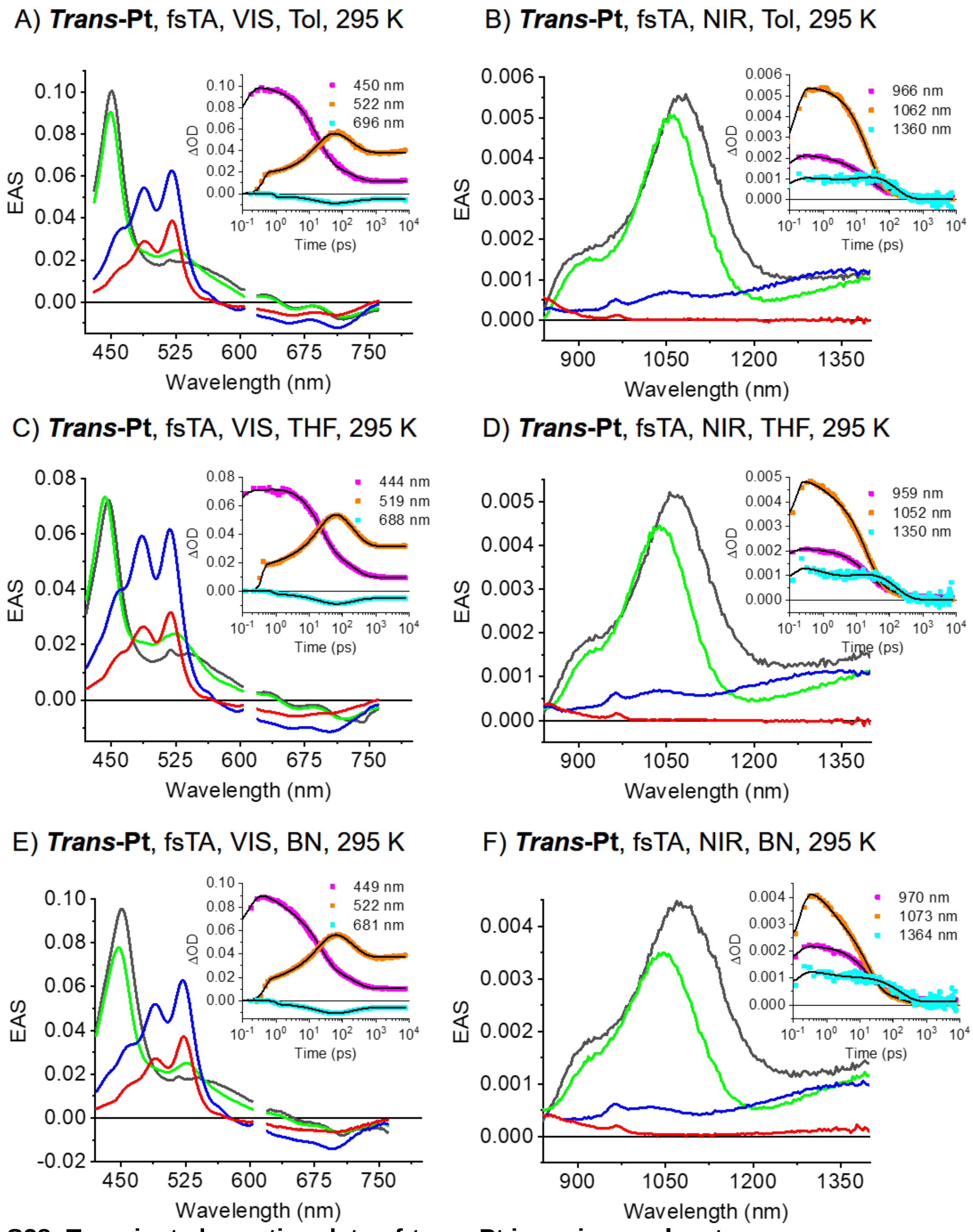

Figure S28: Transient absorption data of *trans*-Pt in various solvents

All figures show the EAS of the $(S_0S_1)^*$ state before solvent relaxation (grey), the (S_0S_1) state after solvent relaxation (green), the correlated triplet pair state (T_1T_1) (blue) and the decorrelation triplet state $(T_1 + T_1)$ or (S_0T_1) (red) of ***trans*-Pt**. The EAS were obtained upon global analysis with a sequential model of fsTA data measured at room temperature. See **Figure S24** for the raw data.

A & B) EAS of ***trans*-Pt** (VIS & NIR) measured in argon saturated toluene.

C & D) EAS of ***trans*-Pt** (VIS & NIR) measured in argon saturated THF.

E & F) EAS of ***trans*-Pt** (VIS & NIR) measured in argon saturated benzonitrile.

Insets: Raw data single wavelength kinetics and fits to the data.

Supplemental transient and steady-state IR data

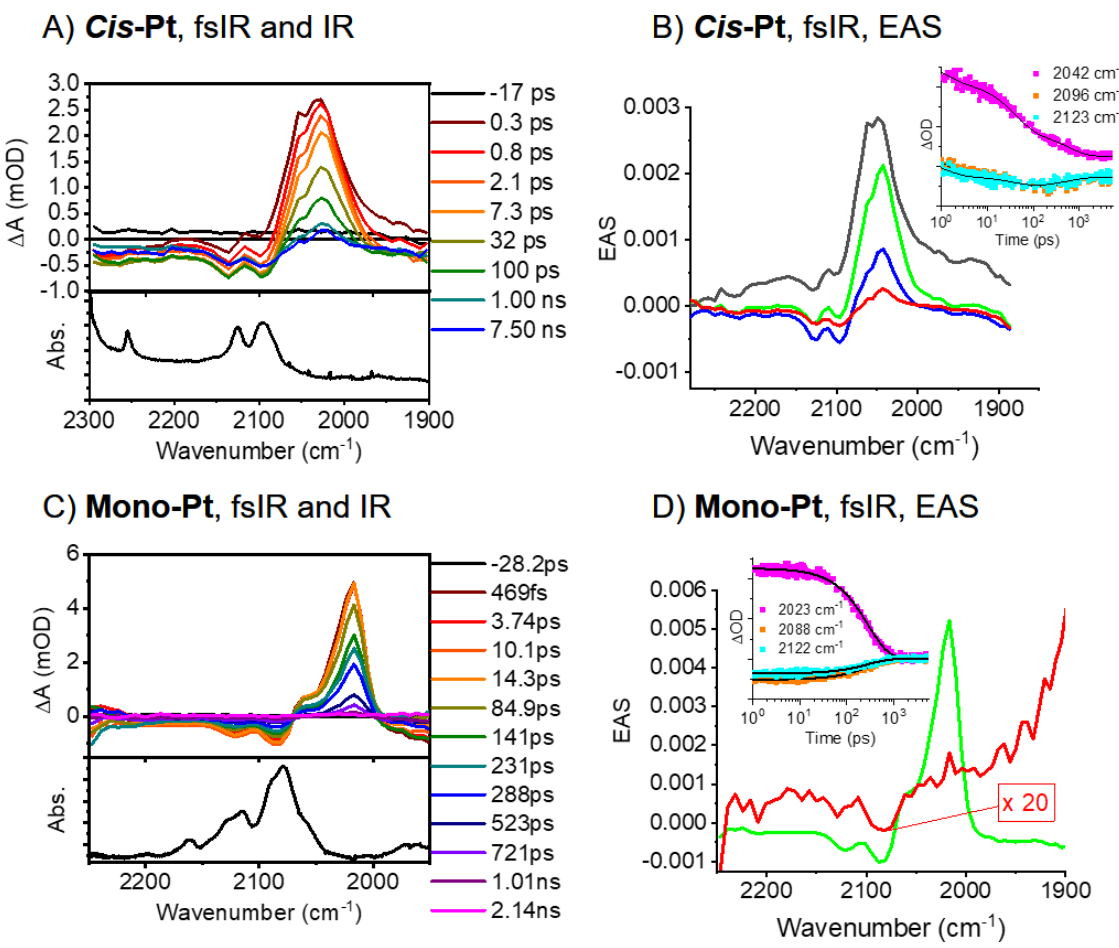

Figure S29: Transient and steady-state IR absorption spectra of *cis*- and mono-Pt
Measured at room temperature in oxygen-free dichloromethane. The transient IR spectra were excited at 650 nm.
A) Transient and steady-state IR absorption spectra of ***cis*-Pt**.
B) EAS of the (S_0S_1)* state before solvent relaxation (gray), (S_0S_1) state after solvent relaxation (green), the correlated triplet pair state (T_1T_1) (blue) and the decorrelation triplet state (T_1S_0) (red) of ***cis*-Pt** obtained upon global analysis with a sequential model of fsIR data. See A) for the raw data. Inset: Raw data single-wavenumber kinetics and fits to the data.
C) Transient and steady-state IR absorption spectra of **mono-Pt**.
D) EAS of the (S_1) state after solvent relaxation (green) and the triplet state (T_1) (red) of **mono-Pt** obtained upon global analysis with a sequential model of fsIR data. See C) for the raw data. Inset: Raw data single-wavenumber kinetics and fits to the data.

Comparison of *trans-* and *cis*-Pt with other covalent pentacene dimers

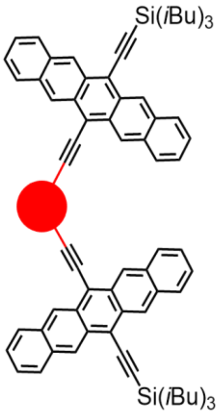

Linker	Molecule	τ(S_0S_1)	τ(T_1T_1)	τ(T_1)
—Pt—	***trans*-Pt**	21 ± 1 ps	200 ± 10 ps	330 ± 20 ns
Pt	***cis*-Pt**	49 ± 2 ps	620 ± 30 ps	610 ± 30 ns
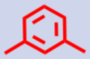	***m*-3**	16 ± 1 ps / 63 ± 3 ps	2.2 ± 0.1 ns	-
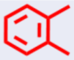	***o*-3**	0.5 ± 0.2 ps	12 ± 1 ps	-
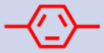	***p*-3**	2.7 ± 0.9 ps	17 ± 1 ps	-
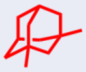	**Nc-*m***	400 ± 10 ps	14 ± 1 ns / 95 ± 1 ns	32 ± 0.5 µs
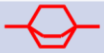	**Nc-*p***	4.0 ± 0.2 ns	130 ± 5 ns	34 ± 2 µs

Figure S30: Comparison of *trans-* and *cis*-Pt with other covalent pentacene dimers.[19, 21, 22] The lifetimes of ***trans-*** and ***cis*-Pt** were measured in butyronitrile, while those of **m-3**, **o-3**, **p-3**, **Nc-m** and **Nc-p** were measured in benzonitrile. τ(S_0S_1) is the measured singlet deactivation time, not the corrected singlet fission lifetime.

Supplemental theory results

HF/LANL2DZ-optimized geometries

Trans-Pt

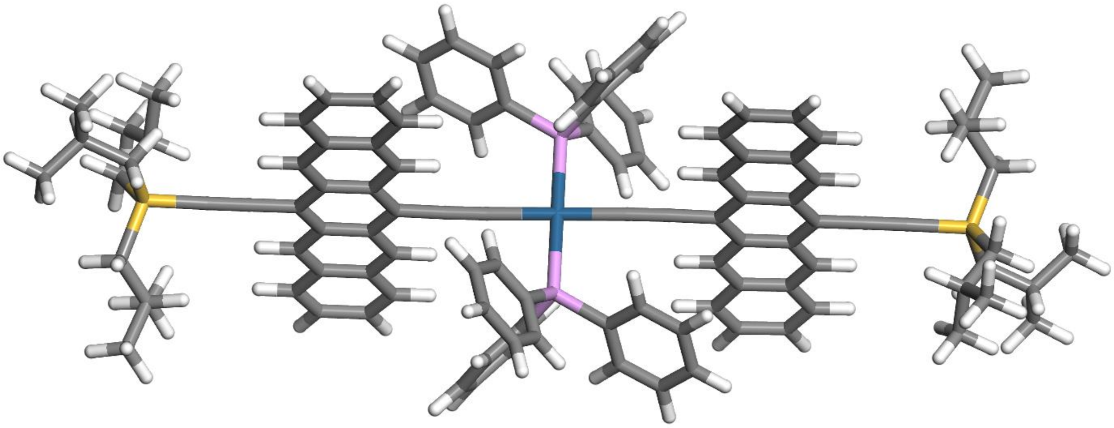

Optimized geometry (coordinates in Å) of *trans*-Pt.

Atomic Number	x	y	z
15	-0.0037	0.98601	-2.25435
14	-12.04633	0.05461	0.21757
6	-6.85934	-1.25354	-0.23128
6	-2.04048	-0.08909	0.02538
6	-7.54627	-0.06324	0.09037
6	-3.25695	-0.10365	0.03954
6	-6.82687	1.11757	0.37499
6	-8.98274	-0.04903	0.12291
6	-7.48351	2.33722	0.69584
1	-8.55343	2.34764	0.73203
6	-10.19293	-0.02919	0.1518
6	-6.78854	3.49557	0.95656
6	-12.65451	-0.99138	1.70222
1	-12.72128	-0.31605	2.55306
1	-13.67511	-1.31285	1.49739
6	-7.45751	4.73991	1.28342
1	-8.52964	4.74433	1.3289
6	-11.81907	-2.22532	2.12869
1	-10.79815	-1.90021	2.30079
6	-4.69085	2.31645	0.59925
1	-3.62208	2.30718	0.54161
6	-11.79003	-3.32155	1.04699
1	-12.79606	-3.65758	0.80951
1	-11.22433	-4.18215	1.38981
1	-11.32762	-2.97025	0.13141
6	-5.38641	1.10552	0.33964
6	-12.36219	-2.80039	3.45195
1	-11.76582	-3.64478	3.78501
1	-13.38733	-3.14115	3.33355
1	-12.34837	-2.05212	4.238

**6**	-12.46093	1.90125	0.50551
**1**	-12.23527	2.12101	1.54914
**1**	-11.74916	2.48005	-0.0776
**6**	-13.8878	2.41037	0.18258
**1**	-14.10041	2.19636	-0.86133
**6**	-13.95779	3.93988	0.36447
**1**	-13.75656	4.21349	1.39651
**1**	-14.94064	4.32123	0.1034
**1**	-13.22731	4.44169	-0.26191
**6**	-14.97131	1.72486	1.03678
**1**	-15.0192	0.65772	0.85116
**1**	-15.95096	2.13808	0.81887
**1**	-14.77689	1.87019	2.09559
**6**	-12.78342	-0.62969	-1.41165
**1**	-12.9297	-1.69642	-1.2631
**1**	-13.78271	-0.21084	-1.52819
**6**	-11.99441	-0.42378	-2.72923
**1**	-10.99289	-0.81878	-2.58909
**6**	-12.65866	-1.21388	-3.87443
**1**	-12.72017	-2.2713	-3.63693
**1**	-12.09557	-1.11154	-4.79741
**1**	-13.66766	-0.85397	-4.05717
**6**	-11.86373	1.06156	-3.11419
**1**	-12.84294	1.51804	-3.23226
**1**	-11.33311	1.16763	-4.05501
**1**	-11.31516	1.6231	-2.36732
**6**	-4.69175	-0.09154	0.04487
**6**	-5.41808	-1.26861	-0.24844
**6**	-4.75315	-2.48107	-0.5753
**1**	-3.68243	-2.48711	-0.57474
**6**	-5.44142	-3.63054	-0.88758
**6**	-4.76622	-4.86353	-1.24
**1**	-3.69368	-4.86581	-1.25695
**6**	-5.4662	-5.98128	-1.5447
**1**	-4.95904	-6.88859	-1.80902
**6**	-6.91535	-5.96877	-1.5207
**1**	-7.44586	-6.86855	-1.7644
**6**	-7.59058	-4.84083	-1.19823
**1**	-8.66352	-4.83067	-1.18169
**6**	-6.88631	-3.61657	-0.86912
**6**	-7.54982	-2.4559	-0.54494
**1**	-8.62022	-2.44931	-0.53055
**6**	0.75622	-0.07186	-3.59248
**6**	0.42521	0.13577	-4.93789
**1**	-0.28283	0.89127	-5.21166
**6**	1.00473	-0.64445	-5.93608
**1**	0.74163	-0.47594	-6.96174
**6**	1.92189	-1.64217	-5.60088
**1**	2.36837	-2.24255	-6.36912
**6**	2.25427	-1.85486	-4.26456
**1**	2.96217	-2.61451	-3.99839
**6**	1.6721	-1.07422	-3.26449
**1**	1.94137	-1.23871	-2.2441

**6**	-6.75136	5.86924	1.52505
**1**	-7.25587	6.78453	1.76592
**6**	-5.30333	5.86347	1.4607
**1**	-4.77119	6.77459	1.65255
**6**	-4.63537	4.72502	1.16045
**1**	-3.56317	4.71706	1.10786
**6**	-5.34492	3.48833	0.89947
**6**	0.9583	2.58116	-2.23405
**6**	0.71835	3.48315	-1.19054
**1**	0.01499	3.23797	-0.41872
**6**	1.39241	4.69972	-1.14067
**1**	1.19468	5.38624	-0.34083
**6**	2.33285	5.01908	-2.12285
**1**	2.86497	5.94864	-2.07669
**6**	2.58864	4.11691	-3.15229
**1**	3.32084	4.34734	-3.90056
**6**	1.90065	2.90255	-3.2102
**1**	2.11737	2.21801	-4.00398
**6**	-1.65766	1.44508	-2.99015
**6**	-2.57745	0.42297	-3.25319
**1**	-2.33444	-0.59419	-3.02117
**6**	-3.81957	0.71507	-3.80537
**1**	-4.51669	-0.07697	-3.99383
**6**	-4.16359	2.03792	-4.09433
**1**	-5.12588	2.26488	-4.50922
**6**	-3.25503	3.05945	-3.83303
**1**	-3.5109	4.07796	-4.0495
**6**	-2.00401	2.76398	-3.2843
**1**	-1.31783	3.5641	-3.09943
**15**	0.00371	-0.98625	2.25401
**14**	12.04635	-0.05433	-0.21711
**6**	6.85926	1.25364	0.23131
**6**	2.04047	0.08897	-0.02566
**6**	7.54627	0.06336	-0.09027
**6**	3.25695	0.10357	-0.03974
**6**	6.82695	-1.11749	-0.37491
**6**	8.98274	0.04921	-0.12271
**6**	7.48367	-2.33711	-0.69568
**1**	8.55359	-2.34749	-0.73178
**6**	10.19294	0.02941	-0.1515
**6**	6.78877	-3.49551	-0.95642
**6**	12.65462	0.99165	-1.70172
**1**	12.72148	0.3163	-2.55255
**1**	13.6752	1.31315	-1.49682
**6**	7.45782	-4.73982	-1.28319
**1**	8.52995	-4.7442	-1.32859
**6**	11.81918	2.22556	-2.12829
**1**	10.79829	1.90041	-2.30047
**6**	4.691	-2.31648	-0.59929
**1**	3.62222	-2.30726	-0.54174
**6**	11.79003	3.3218	-1.04661
**1**	12.79602	3.65787	-0.80905
**1**	11.22433	4.18239	-1.3895

**1**	11.32755	2.97051	-0.13107
**6**	5.38648	-1.10551	-0.33967
**6**	12.3624	2.80062	-3.45152
**1**	11.76604	3.64499	-3.78465
**1**	13.38752	3.1414	-3.33303
**1**	12.34867	2.05233	-4.23756
**6**	12.46102	-1.90097	-0.50498
**1**	12.23546	-2.12075	-1.54862
**1**	11.74922	-2.47977	0.07808
**6**	13.88788	-2.41003	-0.18191
**1**	14.1004	-2.196	0.86201
**6**	13.95793	-3.93955	-0.36377
**1**	13.7568	-4.21318	-1.39582
**1**	14.94077	-4.32087	-0.10261
**1**	13.22742	-4.44137	0.26255
**6**	14.97144	-1.72451	-1.03604
**1**	15.01929	-0.65736	-0.85045
**1**	15.95109	-2.1377	-0.81804
**1**	14.77711	-1.86987	-2.09486
**6**	12.78328	0.63002	1.41216
**1**	12.92953	1.69676	1.26361
**1**	13.78257	0.21121	1.52879
**6**	11.99416	0.42411	2.72967
**1**	10.99264	0.81908	2.58945
**6**	12.65829	1.21426	3.87492
**1**	12.7198	2.27167	3.6374
**1**	12.09513	1.11191	4.79786
**1**	13.66728	0.85438	4.05775
**6**	11.86349	-1.06122	3.11466
**1**	12.8427	-1.51767	3.23281
**1**	11.3328	-1.16729	4.05543
**1**	11.315	-1.62279	2.36775
**6**	4.69175	0.09153	-0.04499
**6**	5.418	1.26864	0.24836
**6**	4.75299	2.48107	0.57517
**1**	3.68227	2.48706	0.57454
**6**	5.44119	3.63057	0.88749
**6**	4.76591	4.86353	1.23986
**1**	3.69337	4.86577	1.25673
**6**	5.46582	5.98132	1.5446
**1**	4.95859	6.8886	1.80889
**6**	6.91497	5.96887	1.5207
**1**	7.44542	6.86868	1.76443
**6**	7.59027	4.84096	1.19827
**1**	8.66321	4.83086	1.1818
**6**	6.88608	3.61668	0.86913
**6**	7.54967	2.45603	0.545
**1**	8.62006	2.4495	0.53068
**6**	-0.75619	0.07162	3.59215
**6**	-0.42514	-0.13598	4.93756
**1**	0.28292	-0.89147	5.21132
**6**	-1.00466	0.64424	5.93575
**1**	-0.74153	0.47575	6.96141

**6**	-1.92185	1.64193	5.60056
**1**	-2.36833	2.2423	6.36881
**6**	-2.25427	1.85459	4.26425
**1**	-2.9622	2.61422	3.99809
**6**	-1.6721	1.07395	3.26417
**1**	-1.94141	1.23842	2.24379
**6**	6.75175	-5.8692	-1.52484
**1**	7.25631	-6.78447	-1.76564
**6**	5.30371	-5.8635	-1.46061
**1**	4.77163	-6.77464	-1.65247
**6**	4.63568	-4.72506	-1.16044
**1**	3.56347	-4.71715	-1.10793
**6**	5.34515	-3.48834	-0.89943
**6**	-0.95831	-2.58138	2.23369
**6**	-0.71844	-3.48332	1.19013
**1**	-0.01513	-3.23811	0.41827
**6**	-1.39252	-4.69988	1.14024
**1**	-1.19484	-5.38636	0.34036
**6**	-2.3329	-5.01928	2.12248
**1**	-2.86503	-5.94883	2.0763
**6**	-2.5886	-4.11716	3.15197
**1**	-3.32075	-4.34761	3.90029
**6**	-1.90059	-2.9028	3.20989
**1**	-2.11725	-2.2183	4.00373
**6**	1.65767	-1.44536	2.98978
**6**	2.57748	-0.42327	3.25289
**1**	2.33448	0.5939	3.02093
**6**	3.81959	-0.71542	3.80504
**1**	4.51673	0.0766	3.99354
**6**	4.16361	-2.0383	4.09391
**1**	5.1259	-2.26529	4.50877
**6**	3.25503	-3.0598	3.83255
**1**	3.5109	-4.07832	4.04895
**6**	2.00402	-2.76429	3.28385
**1**	1.31783	-3.56438	3.09893
**78**	-2E-6	-9.1E-5	-1.54E-4

***Cis*-Pt**

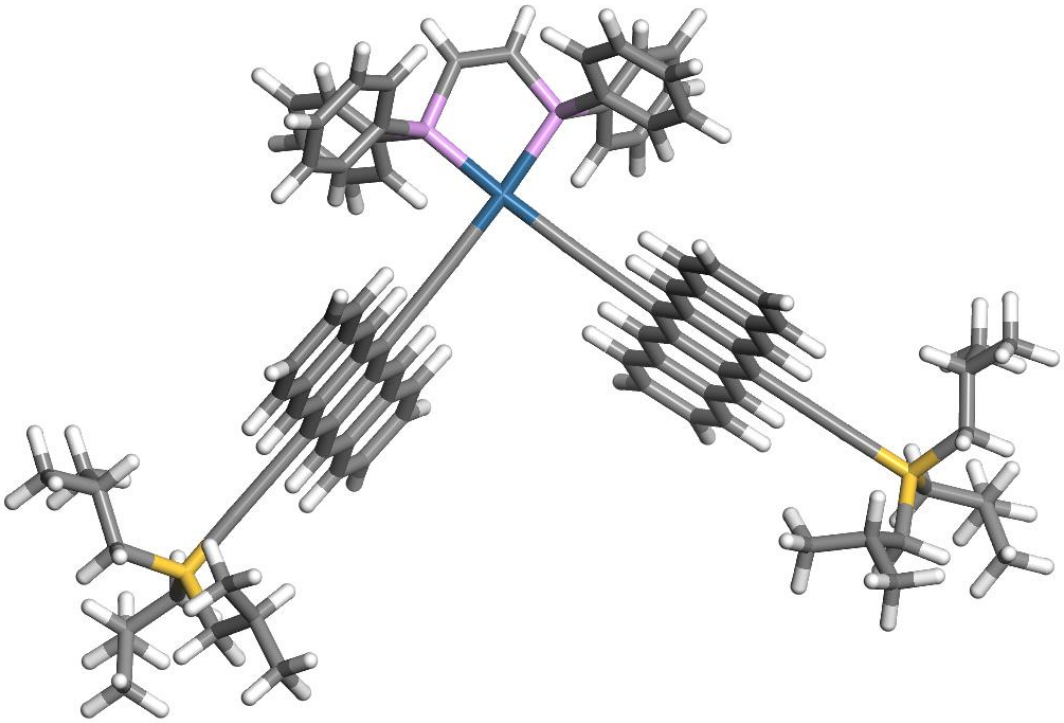

Optimized geometry (coordinates in Å) of *cis*-Pt.

Atomic Number	x	y	z
**15**	1.65227	5.12692	-0.54778
**14**	8.90679	-4.69204	0.35998
**6**	5.13411	-1.00046	1.27374
**6**	1.42236	1.91786	-0.21711
**6**	5.49021	-1.76818	0.14379
**6**	2.3014	1.08401	-0.13199
**6**	4.78059	-1.62232	-1.06799
**6**	6.57777	-2.70309	0.22686
**6**	5.10519	-2.38234	-2.22482
**1**	5.91266	-3.08294	-2.16678
**6**	7.49136	-3.49411	0.29798
**6**	4.41314	-2.24681	-3.40612
**6**	8.43755	-6.23557	1.38381
**1**	7.85476	-6.91288	0.76268
**1**	9.374	-6.75237	1.58281
**6**	4.74321	-3.02214	-4.58623
**1**	5.55816	-3.7177	-4.52618
**6**	7.68908	-6.00941	2.72818
**1**	7.98399	-5.04966	3.1473
**6**	2.98893	-0.56123	-2.38076
**1**	2.1706	0.12765	-2.4285
**6**	8.07069	-7.09547	3.75406
**1**	7.81839	-8.08271	3.37713
**1**	7.54134	-6.95036	4.6912

**1**	9.13573	-7.08081	3.9652
**6**	3.69489	-0.67878	-1.15371
**6**	6.16166	-5.98675	2.52612
**1**	5.65152	-5.76913	3.45945
**1**	5.8143	-6.95497	2.17535
**1**	5.86449	-5.24028	1.80187
**6**	9.25573	-5.16714	-1.46133
**1**	8.49087	-5.88661	-1.75432
**1**	9.06599	-4.27813	-2.05734
**6**	10.64623	-5.72963	-1.84867
**1**	11.40384	-5.01126	-1.54801
**6**	10.74168	-5.89023	-3.3794
**1**	10.00563	-6.60488	-3.7371
**1**	11.72418	-6.24593	-3.67576
**1**	10.56178	-4.94613	-3.8834
**6**	10.962	-7.06888	-1.15585
**1**	11.02413	-6.96341	-0.07891
**1**	11.91259	-7.46423	-1.49964
**1**	10.19561	-7.80691	-1.37494
**6**	10.37904	-3.84989	1.24785
**1**	10.23092	-4.03322	2.31107
**1**	11.28891	-4.38769	0.9819
**6**	10.61017	-2.33189	1.04415
**1**	9.68776	-1.81423	1.28784
**6**	11.70458	-1.82651	2.00543
**1**	11.4418	-2.02807	3.03924
**1**	11.85166	-0.75545	1.9014
**1**	12.65349	-2.31538	1.80135
**6**	10.97615	-1.97959	-0.40954
**1**	11.88295	-2.49447	-0.71562
**1**	11.14962	-0.91315	-0.51274
**1**	10.18439	-2.24803	-1.099
**6**	3.34797	0.10749	-0.03143
**6**	4.05013	-0.05512	1.18505
**6**	3.7072	0.70013	2.33861
**1**	2.88958	1.388	2.26966
**6**	4.38164	0.56197	3.52938
**6**	4.03015	1.32879	4.70802
**1**	3.21307	2.021	4.63777
**6**	4.70979	1.17497	5.86861
**1**	4.44139	1.7429	6.73787
**6**	5.80809	0.23316	5.9565
**1**	6.32824	0.1289	6.88877
**6**	6.17068	-0.50747	4.88292
**1**	6.98128	-1.20758	4.94924
**6**	5.47433	-0.37962	3.61737
**6**	5.81894	-1.12531	2.51351
**1**	6.62483	-1.82674	2.58426
**6**	4.04193	-2.87741	-5.7349
**1**	4.29303	-3.45736	-6.6015
**6**	2.93716	-1.94253	-5.81369
**1**	2.39467	-1.85605	-6.73475
**6**	2.59283	-1.19702	-4.73779

**1**	1.77039	-0.50903	-4.78831
**6**	3.31585	-1.31002	-3.48686
**6**	2.80504	5.10965	-2.00347
**6**	2.6814	4.10607	-2.9654
**1**	1.95173	3.33228	-2.84385
**6**	3.52724	4.09403	-4.0763
**1**	3.43567	3.31373	-4.80517
**6**	4.49519	5.0842	-4.228
**1**	5.14693	5.07162	-5.07921
**6**	4.62513	6.0874	-3.26384
**1**	5.37597	6.84476	-3.37291
**6**	3.78524	6.09772	-2.15403
**1**	3.90914	6.85967	-1.40878
**6**	2.73135	5.46375	0.9277
**6**	2.43953	6.4857	1.83349
**1**	1.59459	7.12764	1.68159
**6**	3.24416	6.67932	2.95733
**1**	3.01319	7.46662	3.64765
**6**	4.33982	5.84822	3.18354
**1**	4.95564	5.99266	4.04909
**6**	4.62958	4.82072	2.28363
**1**	5.46173	4.16904	2.4593
**6**	3.82948	4.62819	1.16019
**1**	4.05645	3.8289	0.48369
**15**	-1.6472	5.1312	-0.53751
**14**	-8.89578	-4.70308	0.37891
**6**	-5.13288	-0.98886	1.30289
**6**	-1.42402	1.92186	-0.20949
**6**	-5.50113	-1.75207	0.17395
**6**	-2.30515	1.09072	-0.11993
**6**	-4.80139	-1.60442	-1.04345
**6**	-6.59047	-2.68486	0.26205
**6**	-5.13784	-2.35982	-2.19996
**1**	-5.94663	-3.05842	-2.13732
**6**	-7.50726	-3.47277	0.32871
**6**	-4.45553	-2.22248	-3.3867
**6**	-8.37683	-6.16946	1.49607
**1**	-7.91615	-6.90985	0.84491
**1**	-9.28006	-6.63761	1.88603
**6**	-4.79789	-2.99315	-4.56637
**1**	-5.6141	-3.6868	-4.50142
**6**	-7.40895	-5.89373	2.67498
**1**	-6.52658	-5.40041	2.28077
**6**	-3.01781	-0.54433	-2.36821
**1**	-2.19796	0.14238	-2.42073
**6**	-8.02744	-4.97318	3.74396
**1**	-8.93927	-5.40638	4.14707
**1**	-7.33639	-4.82639	4.56797
**1**	-8.26846	-3.99563	3.34152
**6**	-3.71348	-0.6639	-1.13548
**6**	-6.96171	-7.22319	3.31455
**1**	-6.25401	-7.05053	4.11998
**1**	-7.81254	-7.7599	3.72596

**1**	-6.48315	-7.86682	2.5834
**6**	-9.11066	-5.30076	-1.42716
**1**	-8.28205	-5.97593	-1.64125
**1**	-8.95776	-4.43546	-2.06708
**6**	-10.43673	-5.98702	-1.83928
**1**	-11.25582	-5.30382	-1.63146
**6**	-10.43992	-6.26475	-3.35598
**1**	-9.63967	-6.94954	-3.62259
**1**	-11.37927	-6.7097	-3.67124
**1**	-10.29719	-5.34929	-3.92108
**6**	-10.69848	-7.29077	-1.06125
**1**	-10.80789	-7.11308	0.00268
**1**	-11.60929	-7.76885	-1.40761
**1**	-9.88104	-7.99273	-1.19902
**6**	-10.47189	-3.87169	1.08021
**1**	-10.44521	-4.01391	2.15747
**1**	-11.33359	-4.4403	0.73121
**6**	-10.71031	-2.36812	0.79184
**1**	-9.8326	-1.81805	1.11691
**6**	-11.91695	-1.85671	1.60399
**1**	-11.76853	-2.01432	2.66772
**1**	-12.07416	-0.7942	1.44279
**1**	-12.82602	-2.37668	1.31357
**6**	-10.91714	-2.07899	-0.70653
**1**	-11.76612	-2.63593	-1.09422
**1**	-11.11065	-1.0235	-0.86941
**1**	-10.04314	-2.3422	-1.29029
**6**	-3.35427	0.11749	-0.01368
**6**	-4.04661	-0.04676	1.20811
**6**	-3.69151	0.70364	2.36119
**1**	-2.87237	1.38921	2.28751
**6**	-4.35614	0.56358	3.55724
**6**	-3.9922	1.32538	4.73537
**1**	-3.17363	2.01533	4.66045
**6**	-4.66239	1.16969	5.9012
**1**	-4.38486	1.73389	6.77002
**6**	-5.76276	0.23088	5.99538
**1**	-6.27517	0.12498	6.93174
**6**	-6.13689	-0.50504	4.92251
**1**	-6.94892	-1.20304	4.99342
**6**	-5.451	-0.37488	3.65146
**6**	-5.80746	-1.11546	2.54796
**1**	-6.61528	-1.81393	2.62295
**6**	-2.80951	5.11732	-1.98563
**6**	-2.69365	4.11466	-2.94948
**1**	-1.96435	3.33966	-2.83356
**6**	-3.54675	4.10513	-4.05483
**1**	-3.46117	3.3255	-4.78514
**6**	-4.51424	5.09688	-4.19903
**1**	-5.1716	5.08618	-5.04593
**6**	-4.63639	6.09915	-3.23291
**1**	-5.38686	6.85769	-3.33616
**6**	-3.78923	6.10698	-2.12861

**1**	-3.90714	6.86822	-1.38167
**6**	-4.10628	-2.84655	-5.72065
**1**	-4.36645	-3.42303	-6.58688
**6**	-2.9998	-1.91426	-5.80606
**1**	-2.46525	-1.82609	-6.7316
**6**	-2.64408	-1.17321	-4.73078
**1**	-1.82037	-0.48712	-4.78629
**6**	-3.35642	-1.28845	-3.47393
**6**	-2.71568	5.47025	0.94516
**6**	-2.41741	6.49326	1.84764
**1**	-1.57342	7.13486	1.68914
**6**	-3.2142	6.6884	2.9768
**1**	-2.97831	7.4765	3.66454
**6**	-4.30843	5.8578	3.2116
**1**	-4.9182	6.00342	4.08123
**6**	-4.60464	4.82928	2.31495
**1**	-5.4357	4.178	2.49714
**6**	-3.81237	4.63522	1.18624
**1**	-4.04416	3.83514	0.51233
**78**	7.38E-4	3.32615	-0.35842
**6**	0.66984	6.68939	-0.78064
**6**	-0.6622	6.69113	-0.7765
**1**	1.21223	7.60314	-0.9543
**1**	-1.20327	7.60628	-0.94687

PM6 (CIS=96) calculated spectra

Trans-Pt

PM6 (CIS=96) calculated spectra of *trans*-Pt in the gas phase

State	eV	nm	Δμ (Debye)	Osc.	
1	0.000	0.0			Singlet
2	1.287	963.4	1.08		Triplet
3	1.315	942.8	0.78	0.285	Singlet
4	1.315	942.6	0.98		Triplet
5	1.398	887.0	0.87	0.054	Singlet
6	1.737	713.7	1.16	0.001	Singlet
7	2.058	602.4	0.48		Triplet
8	2.128	582.5	1.00	0.045	Singlet
9	2.210	561.0	0.26		Triplet
10	2.352	527.0	0.83	0.047	Singlet
11	2.407	515.0	0.56		Triplet
12	2.451	505.9	0.49		Triplet
13	2.468	502.3	0.68		Triplet
14	2.549	486.4	0.23		Triplet
15	2.602	476.6	0.41	0.037	Singlet
16	2.631	471.3	0.40	0.073	Singlet
17	2.716	456.5	0.58		Triplet
18	2.770	447.6	0.84	0.318	Singlet
19	2.773	447.1	1.32	0.078	Singlet
20	2.785	445.2	0.47		Triplet
21	2.885	429.7	0.38		Triplet
22	2.886	429.6	0.36		Triplet
23	2.906	426.6	0.46		Triplet
24	3.048	406.8	0.45		Triplet
25	3.050	406.4	1.18	0.012	Singlet
26	3.060	405.2	0.08		Triplet
27	3.138	395.1	0.40		Triplet
28	3.162	392.2	0.24		Triplet
29	3.162	392.1	0.24		Triplet
30	3.231	383.7	0.69		Triplet
31	3.233	383.5	1.47	0.035	Singlet
32	3.234	383.4	0.87		Triplet
33	3.258	380.5	0.48		Triplet
34	3.308	374.8	0.73	0.018	Singlet

35	3.351	370.0	0.63		Triplet
36	3.357	369.3	1.48	0.533	Singlet
37	3.373	367.6	0.93		Triplet
38	3.373	367.5	0.90		Triplet
39	3.379	366.9	2.19	0.131	Singlet
40	3.400	364.6	8.78		Triplet
41	3.413	363.3	0.78		Triplet
42	3.426	361.9	0.59		Triplet
43	3.439	360.5	0.45		Triplet
44	3.443	360.0	0.33		Triplet
45	3.451	359.2	6.17	0.089	Singlet
46	3.494	354.9	8.00		Triplet
47	3.515	352.7	0.28		Triplet
48	3.519	352.3	1.21		Triplet
49	3.523	351.9	1.90	0.220	Singlet
50	3.530	351.3	1.18		Triplet
51	3.539	350.4	0.68	0.079	Singlet
52	3.543	349.9	5.02	0.039	Singlet
53	3.557	348.5	0.34	0.136	Singlet
54	3.589	345.5	1.60		Triplet
55	3.597	344.7	0.21		Triplet
56	3.634	341.2	2.24	0.111	Singlet
57	3.680	336.9	0.31	0.958	Singlet
58	3.695	335.6	0.44		Triplet
59	3.712	334.0	0.29	0.035	Singlet
60	3.712	334.0	0.42	0.006	Singlet
61	3.718	333.5	0.28	0.101	Singlet
62	3.720	333.3	0.16	0.146	Singlet
63	3.727	332.7	0.31		Triplet
64	3.731	332.3	0.54	0.006	Singlet
65	3.737	331.8	0.53	0.156	Singlet
66	3.761	329.6	0.20	0.008	Singlet
67	3.766	329.2	0.24	0.013	Singlet
68	3.784	327.6	3.44	0.060	Singlet
69	3.791	327.0	1.48		Triplet
70	3.795	326.7	0.19	0.290	Singlet
71	3.805	325.8	0.55	1.078	Singlet
72	3.819	324.7	0.17		Triplet
73	3.823	324.3	0.16		Triplet

74	3.836	323.2	1.13		Triplet	
75	3.859	321.3	0.99		Triplet	
76	3.860	321.2	0.95		Triplet	
77	3.892	318.6	2.70	0.394	Singlet	
78	3.893	318.5	2.29		Triplet	
79	3.896	318.2	1.36		Triplet	
80	3.935	315.1	1.68		Triplet	
81	3.943	314.4	1.65		Triplet	
82	3.945	314.2	0.62		Triplet	
83	3.948	314.0	1.07		Triplet	
84	3.977	311.8	1.34	0.146	Singlet	
85	4.065	305.0	0.88	0.828	Singlet	
86	4.131	300.1	1.25	0.208	Singlet	
87	4.132	300.0	9.98		Triplet	
88	4.150	298.7	1.43		Triplet	
89	4.152	298.6	1.27	0.000	Singlet	
90	4.178	296.8	1.27		Triplet	
91	4.183	296.4	1.18		Triplet	
92	4.229	293.2	0.48		Triplet	
93	4.290	289.0	2.31		Triplet	
94	4.300	288.3	2.09	0.052	Singlet	
95	4.351	285.0	3.14	0.005	Singlet	
96	4.368	283.8	8.17		Triplet	
97	4.373	283.5	1.58	0.016	Singlet	
98	4.401	281.7	1.25		Triplet	
99	4.428	280.0	1.30	0.041	Singlet	
100	4.452	278.5	3.62		Triplet	
101	4.453	278.4	3.65	0.000	Singlet	
102	4.462	277.8	13.60		Triplet	CT
103	4.470	277.4	8.31	0.030	Singlet	
104	4.508	275.0	18.75		Triplet	CT
105	4.512	274.8	5.92		Triplet	
106	4.512	274.8	5.27	0.001	Singlet	
107	4.521	274.2	3.56	0.010	Singlet	
108	4.522	274.2	5.43		Triplet	
109	4.527	273.9	4.19		Triplet	
110	4.540	273.1	2.13		Triplet	
111	4.552	272.4	19.26	0.057	Singlet	CT
112	4.562	271.8	4.24		Triplet	

113	4.562	271.7	1.75	0.068	Singlet	
114	4.571	271.2	3.28	0.021	Singlet	
115	4.571	271.2	3.29		Triplet	
116	4.576	271.0	11.87	0.169	Singlet	CT
117	4.576	270.9	2.57		Triplet	
118	4.594	269.9	16.14	0.010	Singlet	CT
119	4.600	269.5	7.40		Triplet	
120	4.601	269.5	6.75	0.002	Singlet	
121	4.612	268.8	1.82		Triplet	
122	4.614	268.7	1.52	0.002	Singlet	
123	4.622	268.2	6.53	0.002	Singlet	
124	4.624	268.1	6.51		Triplet	
125	4.649	266.7	4.33	0.109	Singlet	
126	4.650	266.6	10.09		Triplet	CT
127	4.654	266.4	13.12		Triplet	CT
128	4.665	265.8	3.06		Triplet	
129	4.676	265.1	1.68	0.557	Singlet	
130	4.694	264.1	8.88		Triplet	
131	4.701	263.8	8.35		Triplet	
132	4.716	262.9	7.29		Triplet	
133	4.717	262.9	7.25	0.000	Singlet	
134	4.717	262.8	6.01		Triplet	
135	4.718	262.8	6.09	0.006	Singlet	
136	4.730	262.1	2.80	0.117	Singlet	
137	4.738	261.7	3.42		Triplet	
138	4.748	261.1	0.73	0.063	Singlet	
139	4.771	259.8	3.99		Triplet	
140	4.772	259.8	6.14	0.029	Singlet	
141	4.773	259.7	14.60		Triplet	CT
142	4.776	259.6	15.47	0.069	Singlet	CT
143	4.782	259.3	20.44		Triplet	CT
144	4.782	259.3	19.71	0.001	Singlet	CT
145	4.787	259.0	7.64	0.185	Singlet	
146	4.793	258.7	7.12		Triplet	
147	4.804	258.1	0.74		Triplet	
148	4.823	257.0	7.45	0.022	Singlet	
149	4.824	257.0	7.82		Triplet	
150	4.826	256.9	1.97	0.288	Singlet	
151	4.851	255.6	22.70		Triplet	CT

152	4.851	255.6	22.65	0.000	Singlet	CT
153	4.853	255.4	24.50		Triplet	CT
154	4.854	255.4	24.46	0.000	Singlet	CT
155	4.917	252.2	6.56	0.025	Singlet	
156	4.917	252.1	7.04		Triplet	
157	4.925	251.7	3.85	0.127	Singlet	
158	4.935	251.2	7.90	0.004	Singlet	
159	4.937	251.1	7.07		Triplet	
160	4.956	250.1	5.40	0.526	Singlet	
161	4.966	249.7	7.21	0.021	Singlet	
162	4.968	249.6	7.35		Triplet	
163	4.974	249.2	10.46	0.002	Singlet	
164	4.974	249.2	11.76		Triplet	
165	4.977	249.1	13.09	0.002	Singlet	

PM6 (CIS=96) calculated spectra of *trans*-Pt in benzonitrile

State	eV	nm	Δμ (Debye)	Osc.		
1	0	0			Singlet	
2	1.275	972.7	1.27		Triplet	
3	1.326	935.2	0.97		Triplet	
4	1.347	920.5	1	0.258	Singlet	
5	1.449	855.8	0.86	0.076	Singlet	
6	1.734	714.9	0.89	0.001	Singlet	
7	2.065	600.5	0.55		Triplet	
8	2.086	594.3	0.58	0.06	Singlet	
9	2.265	547.4	0.33		Triplet	
10	2.358	525.7	1.09	0.03	Singlet	
11	2.385	519.9	0.7		Triplet	
12	2.414	513.7	0.59		Triplet	
13	2.493	497.4	0.93		Triplet	
14	2.572	482.1	0.85	0.035	Singlet	
15	2.583	480	0.74		Triplet	
16	2.638	469.9	1.25	0.012	Singlet	
17	2.643	469	0.55		Triplet	
18	2.708	457.9	0.14		Triplet	
19	2.74	452.5	1.85	0.265	Singlet	
20	2.846	435.7	13.64		Triplet	CT
21	2.863	433.1	0.73	0.117	Singlet	

22	2.884	429.9	0.32		Triplet	
23	2.891	428.8	0.32		Triplet	
24	2.909	426.1	0.58		Triplet	
25	2.917	425.1	13.06	0.067	Singlet	CT
26	3.025	409.8	1.26		Triplet	
27	3.048	406.7	0.33		Triplet	
28	3.052	406.3	1	0.035	Singlet	
29	3.107	399	4.16		Triplet	
30	3.116	397.8	0.2		Triplet	
31	3.128	396.4	1.14		Triplet	
32	3.186	389.1	5.41		Triplet	
33	3.201	387.3	7.61	0.064	Singlet	
34	3.215	385.6	0.24		Triplet	
35	3.249	381.6	5.39		Triplet	
36	3.263	379.9	0.54		Triplet	
37	3.275	378.6	7.35	0.021	Singlet	
38	3.346	370.5	1.07	0.019	Singlet	
39	3.364	368.5	0.89		Triplet	
40	3.376	367.2	0.42		Triplet	
41	3.376	367.2	0.77		Triplet	
42	3.397	365	1.4	0.052	Singlet	
43	3.419	362.6	0.57		Triplet	
44	3.445	359.9	1.92		Triplet	
45	3.456	358.7	0.16		Triplet	
46	3.467	357.6	0.35		Triplet	
47	3.484	355.8	0.8	0.156	Singlet	
48	3.493	354.9	0.74		Triplet	
49	3.501	354.1	0.45	0.939	Singlet	
50	3.517	352.5	3.16	0.636	Singlet	
51	3.53	351.3	0.51		Triplet	
52	3.559	348.4	1.34		Triplet	
53	3.605	343.9	0.17		Triplet	
54	3.61	343.5	1.4		Triplet	
55	3.628	341.8	2.95	0.055	Singlet	CT
56	3.647	339.9	0.49		Triplet	
57	3.679	337	0.57	0.127	Singlet	
58	3.685	336.5	0.41		Triplet	CT
59	3.687	336.2	0.34	0.436	Singlet	
60	3.704	334.7	0.32	0.202	Singlet	

61	3.71	334.2	0.24	0.076	Singlet	
62	3.714	333.8	0.34	0.045	Singlet	
63	3.716	333.6	0.28	0.113	Singlet	CT
64	3.725	332.8	0.41	0.146	Singlet	
65	3.743	331.2	0.64	0.043	Singlet	
66	3.749	330.7	0.45	0.002	Singlet	
67	3.775	328.4	0.63	0.009	Singlet	
68	3.776	328.3	3.43		Triplet	CT
69	3.785	327.6	6.17	0.069	Singlet	
70	3.794	326.8	0.9		Triplet	
71	3.797	326.5	0.87	0.956	Singlet	
72	3.801	326.2	1.15		Triplet	
73	3.824	324.2	1.44		Triplet	
74	3.83	323.7	1.18		Triplet	CT
75	3.832	323.6	4.32	0.05	Singlet	CT
76	3.834	323.3	2.79		Triplet	
77	3.86	321.2	4.32	0.656	Singlet	CT
78	3.879	319.6	2.69		Triplet	CT
79	3.886	319.1	1.33		Triplet	CT
80	3.908	317.3	0.88		Triplet	CT
81	3.914	316.8	3.09	0.023	Singlet	
82	3.921	316.2	1.1		Triplet	
83	3.926	315.8	1.19		Triplet	
84	4.077	304.1	15.35		Triplet	CT
85	4.079	303.9	15.26	0.006	Singlet	CT
86	4.092	303	11.06		Triplet	CT
87	4.094	302.9	2.03		Triplet	CT
88	4.109	301.7	1.3	0.056	Singlet	CT
89	4.129	300.3	3.33	0.869	Singlet	CT
90	4.177	296.8	7.55		Triplet	CT
92	4.224	293.5	23.11	0	Singlet	CT
91	4.224	293.5	20.24		Triplet	CT
93	4.225	293.4	1.77		Triplet	
94	4.227	293.3	0.97		Triplet	CT
95	4.277	289.9	7.49		Triplet	CT
96	4.278	289.8	7.71	0.001	Singlet	CT
97	4.288	289.2	6.19		Triplet	CT
98	4.328	286.5	2.16		Triplet	CT
99	4.365	284	28.53		Triplet	CT

100	4.379	283.1	0.99		Triplet	CT
101	4.382	282.9	31.27	0.007	Singlet	CT
102	4.393	282.2	3.69	0.005	Singlet	
103	4.395	282.1	3.4		Triplet	
104	4.414	280.9	1.32	0.019	Singlet	
105	4.419	280.6	1.05	0.067	Singlet	
106	4.448	278.7	5.16		Triplet	
107	4.461	277.9	2.9	0.137	Singlet	CT
108	4.481	276.7	3.79	0.011	Singlet	CT
109	4.482	276.6	8.9		Triplet	
110	4.484	276.5	7.59	0.004	Singlet	
111	4.485	276.4	23.16		Triplet	CT
112	4.487	276.3	13.3	0.014	Singlet	
113	4.5	275.5	0.29		Triplet	
114	4.524	274.1	5.27	0.003	Singlet	
115	4.526	273.9	5.85		Triplet	CT
116	4.533	273.5	8.77		Triplet	CT
117	4.539	273.1	11.77	0.003	Singlet	CT
118	4.541	273	8.29		Triplet	CT
119	4.549	272.6	3.35	0.049	Singlet	
120	4.554	272.2	11.68	0.026	Singlet	CT
121	4.565	271.6	9.22		Triplet	CT
122	4.567	271.5	3.26		Triplet	CT
123	4.57	271.3	8.82	0.013	Singlet	CT
124	4.572	271.2	3.67		Triplet	
125	4.585	270.4	21.12		Triplet	CT
126	4.608	269	10.37		Triplet	
127	4.611	268.9	5.52	0.07	Singlet	
128	4.614	268.7	8.46		Triplet	
129	4.615	268.6	5.08	0.043	Singlet	CT
130	4.646	266.8	8.04	1.182	Singlet	CT
131	4.663	265.9	21.89	0.008	Singlet	CT
132	4.664	265.8	7.63	0.386	Singlet	CT
133	4.67	265.5	9.41		Triplet	CT
134	4.682	264.8	2.67	0.519	Singlet	CT
135	4.701	263.7	7.78		Triplet	CT
136	4.702	263.6	8.01	0.021	Singlet	
137	4.716	262.9	8.05	0.061	Singlet	
138	4.724	262.4	12.2		Triplet	

139	4.733	262	0.96		Triplet	
140	4.745	261.3	3.98		Triplet	
141	4.755	260.7	3.11		Triplet	
142	4.763	260.3	20.94		Triplet	CT
143	4.778	259.5	0.72	0.125	Singlet	
144	4.78	259.4	21.13	0.001	Singlet	CT
145	4.78	259.4	19.47		Triplet	CT
146	4.805	258	5.96	1.344	Singlet	
147	4.812	257.7	9.33	0.139	Singlet	
148	4.813	257.6	17.35		Triplet	CT
149	4.814	257.6	23.5	0.001	Singlet	
150	4.814	257.6	7.23		Triplet	
151	4.82	257.2	14.92		Triplet	CT
153	4.825	257	11.14	0.134	Singlet	
152	4.825	257	13.4		Triplet	CT
154	4.826	256.9	10.22	0.036	Singlet	CT
155	4.83	256.7	13.2		Triplet	
156	4.843	256	18.97	0.018	Singlet	CT
157	4.845	255.9	18.28		Triplet	CT
158	4.865	254.8	9.44	0.476	Singlet	
159	4.874	254.4	22.52	0.267	Singlet	CT
160	4.876	254.3	4.2		Triplet	
162	4.885	253.8	12.1	0.023	Singlet	
161	4.885	253.8	12.65		Triplet	
163	4.899	253.1	11.19	0.015	Singlet	
164	4.901	253	11.34		Triplet	
165	4.911	252.4	16.18		Triplet	CT
166	4.915	252.2	16.16	0.022	Singlet	CT
167	4.92	252	3.2		Triplet	
168	4.932	251.4	5.78	0.149	Singlet	
169	4.935	251.2	8.42		Triplet	
170	4.938	251.1	8.25	0.269	Singlet	
171	4.946	250.7	1.11	0.616	Singlet	
172	4.964	249.8	19.06	0.019	Singlet	CT
173	4.964	249.8	22.56		Triplet	CT
174	4.969	249.5	1.31	0.094	Singlet	
175	4.975	249.2	20.4		Triplet	CT
176	4.984	248.8	4.19		Triplet	
177	4.992	248.3	8.92		Triplet	

178	4.993	248.3	6.7	0.063	Singlet
179	4.995	248.2	8.67	0.317	Singlet

Cis-Pt

PM6 (CIS=96) calculated spectra of *cis*-Pt in the gas phase

State	eV	nm	Δμ (Debye)	Osc.	
1	0.000	0.0			Singlet
2	0.931	1331.4	1.21		Triplet
3	1.332	930.7	0.93		Triplet
4	1.446	857.7	1.83	0.256	Singlet
5	1.727	718.1	0.51		Triplet
6	2.091	592.8	0.48		Triplet
7	2.173	570.6	0.48		Triplet
8	2.183	568.0	1.69	0.331	Singlet
9	2.375	522.1	1.46	0.006	Singlet
10	2.409	514.7	1.40	0.022	Singlet
11	2.434	509.3	0.27		Triplet
12	2.452	505.7	0.58		Triplet
13	2.487	498.5	0.26		Triplet
14	2.616	474.0	0.54	0.021	Singlet
15	2.659	466.2	0.31	0.026	Singlet
16	2.680	462.7	0.50		Triplet
17	2.731	454.0	0.49		Triplet
18	2.774	446.9	0.66		Triplet
19	2.837	437.0	0.93	0.001	Singlet
20	2.914	425.4	0.37		Triplet
21	2.949	420.4	0.95	0.041	Singlet
22	2.954	419.8	0.25		Triplet
23	2.955	419.5	0.47		Triplet
24	2.957	419.3	0.21		Triplet
25	3.035	408.5	0.73		Triplet
26	3.127	396.5	1.00	0.008	Singlet
27	3.151	393.4	0.34		Triplet
28	3.231	383.7	0.48		Triplet
29	3.241	382.6	1.19		Triplet
30	3.247	381.8	1.52	0.049	Singlet
31	3.262	380.1	1.21		Triplet
32	3.267	379.5	0.45		Triplet
33	3.305	375.1	0.76	0.045	Singlet
34	3.329	372.4	1.00		Triplet
35	3.420	362.5	1.02	0.126	Singlet

36	3.427	361.7	1.73		Triplet	
37	3.463	358.0	0.54		Triplet	
38	3.476	356.7	0.79		Triplet	
39	3.550	349.2	0.99		Triplet	
40	3.552	349.1	2.70	0.033	Singlet	
41	3.612	343.2	0.42	0.004	Singlet	
42	3.631	341.4	0.93		Triplet	
43	3.632	341.4	0.08	0.003	Singlet	
44	3.639	340.7	0.47		Triplet	
45	3.646	340.0	2.66		Triplet	
46	3.663	338.5	0.56	0.099	Singlet	
47	3.684	336.6	1.42		Triplet	
48	3.692	335.8	0.07	0.007	Singlet	
49	3.698	335.3	0.07	0.011	Singlet	
50	3.708	334.4	0.91	0.038	Singlet	
51	3.710	334.2	2.49		Triplet	
52	3.714	333.8	3.27		Triplet	
53	3.724	332.9	2.25	0.151	Singlet	
54	3.742	331.3	1.02	0.139	Singlet	
55	3.746	331.0	11.40		Triplet	CT
56	3.783	327.7	2.49	0.083	Singlet	
57	3.786	327.5	2.97	0.001	Singlet	
58	3.815	325.0	12.32		Triplet	CT
59	3.817	324.8	8.24	0.322	Singlet	
60	3.819	324.7	2.49	0.734	Singlet	
61	3.823	324.3	2.38		Triplet	
62	3.825	324.1	0.50		Triplet	
63	3.831	323.6	10.41		Triplet	CT
64	3.836	323.2	3.95	0.338	Singlet	
65	3.837	323.1	0.41		Triplet	
66	3.839	323.0	2.14		Triplet	
67	3.862	321.0	0.31		Triplet	
68	3.879	319.6	10.78	1.055	Singlet	CT
69	3.880	319.6	6.41		Triplet	
70	3.890	318.7	2.29		Triplet	
71	3.895	318.3	1.60		Triplet	
72	3.914	316.8	5.57	1.020	Singlet	
73	3.967	312.5	3.02	0.127	Singlet	
74	3.983	311.3	14.86		Triplet	CT

75	3.985	311.1	14.85	0.015	Singlet	CT
76	4.003	309.7	1.06	1.386	Singlet	
77	4.014	308.8	11.62	0.036	Singlet	CT
78	4.020	308.4	11.92		Triplet	CT
79	4.054	305.8	15.86		Triplet	CT
80	4.055	305.8	15.42	0.007	Singlet	CT
81	4.066	304.9	3.34		Triplet	
82	4.081	303.8	2.79	0.462	Singlet	
83	4.132	300.1	0.89	0.110	Singlet	
84	4.150	298.8	3.67		Triplet	
85	4.160	298.0	6.28		Triplet	
86	4.201	295.1	6.49		Triplet	
87	4.220	293.8	28.34	0.010	Singlet	CT
88	4.230	293.1	27.80		Triplet	CT
89	4.234	292.8	28.28	0.019	Singlet	CT
90	4.237	292.6	23.72		Triplet	CT
91	4.267	290.6	23.07	0.026	Singlet	CT
92	4.272	290.2	21.83		Triplet	CT
93	4.285	289.4	4.89	0.066	Singlet	
94	4.354	284.7	25.35		Triplet	CT
95	4.355	284.7	25.34	0.006	Singlet	CT
96	4.364	284.1	14.14	0.017	Singlet	CT
97	4.370	283.7	26.65		Triplet	CT
98	4.371	283.6	11.50	0.025	Singlet	CT
99	4.389	282.5	26.01		Triplet	CT
100	4.391	282.3	26.09	0.002	Singlet	CT
101	4.431	279.8	0.77		Triplet	
102	4.456	278.3	0.74	0.065	Singlet	
103	4.464	277.7	0.25	0.035	Singlet	
104	4.472	277.2	3.58		Triplet	
105	4.510	274.9	1.54		Triplet	
106	4.516	274.5	2.74		Triplet	
107	4.549	272.5	30.41	0.004	Singlet	CT
108	4.549	272.5	29.35		Triplet	CT
109	4.561	271.8	2.34		Triplet	
110	4.574	271.1	1.08		Triplet	
111	4.577	270.9	1.09		Triplet	
112	4.581	270.6	1.19	0.014	Singlet	
113	4.619	268.4	0.65		Triplet	

114	4.632	267.7	3.70	0.159	Singlet	
115	4.665	265.8	27.58	0.008	Singlet	CT
116	4.669	265.6	27.88	0.003	Singlet	CT
117	4.671	265.4	27.70		Triplet	CT
118	4.672	265.4	27.89		Triplet	CT
119	4.694	264.1	4.29	0.166	Singlet	
120	4.716	262.9	28.19	0.002	Singlet	CT
121	4.716	262.9	28.17		Triplet	CT
122	4.719	262.7	28.20	0.001	Singlet	CT
123	4.719	262.7	28.20		Triplet	CT
124	4.769	260.0	4.04	0.242	Singlet	
125	4.781	259.3	4.49		Triplet	
126	4.787	259.0	2.14		Triplet	
127	4.791	258.8	6.52		Triplet	
128	4.804	258.1	5.55	0.237	Singlet	
129	4.805	258.0	12.15		Triplet	CT
130	4.808	257.8	12.20	0.004	Singlet	CT
131	4.842	256.1	5.03	0.002	Singlet	
132	4.842	256.0	18.99		Triplet	CT
133	4.850	255.6	18.34	0.001	Singlet	CT
134	4.880	254.1	25.63		Triplet	CT
135	4.885	253.8	26.38	0.013	Singlet	CT
136	4.887	253.7	9.62	0.011	Singlet	
137	4.888	253.7	8.94		Triplet	
138	4.901	253.0	6.98	0.023	Singlet	
139	4.902	252.9	8.89		Triplet	
140	4.911	252.4	6.25	0.011	Singlet	
141	4.912	252.4	8.76		Triplet	
143	4.927	251.6	6.67	0.002	Singlet	
142	4.927	251.6	5.95		Triplet	
144	4.934	251.3	2.41		Triplet	
145	4.941	250.9	4.71	0.094	Singlet	
146	4.948	250.6	3.73	0.119	Singlet	
147	4.949	250.5	4.82		Triplet	
148	4.953	250.3	3.28	0.082	Singlet	
149	4.956	250.2	9.26		Triplet	
150	4.964	249.8	4.36	0.017	Singlet	
151	4.966	249.7	10.85		Triplet	CT
152	4.967	249.6	5.33		Triplet	

153	4.978	249.1	10.27	0.015	Singlet	CT
154	4.978	249.0	13.47		Triplet	CT
155	4.979	249.0	5.55	0.103	Singlet	
156	4.984	248.7	13.55		Triplet	CT
157	4.985	248.7	14.29	0.006	Singlet	CT
158	4.992	248.4	3.57	0.028	Singlet	
159	4.999	248.0	3.53	0.045	Singlet	

References

1. D. Lehnherr, A. H. Murray, R. McDonald and R. R. Tykwinski, *Angew. Chem. Int. Ed.*, 2010, **49**, 6190-6194.
2. K. Campbell, R. McDonald, M. J. Ferguson and R. R. Tykwinski, *J. Organomet. Chem.*, 2003, **683**, 379-387.
3. W. M. Khairul, M. A. Fox, N. N. Zaitseva, M. Gaudio, D. S. Yufit, B. W. Skelton, A. H. White, J. A. K. Howard, M. I. Bruce and P. J. Low, *Dalton Transactions*, 2009, DOI: 10.1039/B809960J, 610-620.
4. S. L. Murov, I. Carmichael and G. L. Hug, *Handbook of Photochemistry*, Marcel Dekker, 2 edn., 1993.
5. R. M. Young, S. M. Dyar, J. C. Barnes, M. Juríček, J. F. Stoddart, D. T. Co and M. R. Wasielewski, *J. Phys. Chem. A*, 2013, **117**, 12438-12448.
6. H. Hikawa, M. Oguni and H. Suga, *J. Non-Cryst. Solids*, 1988, **101**, 90-100.
7. M. Oguni, H. Hikawa and H. Suga, *Thermochim. Acta*, 1990, **158**, 143-156.
8. C. M. Mauck, R. M. Young and M. R. Wasielewski, *J. Phys. Chem. A*, 2017, **121**, 784-792.
9. I. H. M. van Stokkum, D. S. Larsen and R. van Grondelle, *Biochim. Biophys. Acta - Bioenergetics*, 2004, **1657**, 82-104.
10. J. J. Snellenburg, S. Laptenok, R. Seger, K. M. Mullen and I. H. M. van Stokkum, *J. Stat. Softw.*, 2012, **49**, 1-22.
11. K. M. Mullen and I. H. Van Stokkum, *J. Stat. Softw.*, 2007, **18**, 1-46.
12. S. Stoll and A. Schweiger, *J Magn Reson*, 2006, **178**, 42-55.
13. T. H. J. Dunning and P. J. Hay,*Gaussian Basis Sets for Molecular Calculations*, in *Methods of Electronic Structure Theory*, ed. H. F. Schaefer III., Springer, 1 edn., 1977, pp. 1-28.
14. P. J. Hay and W. R. Wadt, *J. Chem. Phys.*, 1985, **82**, 270-283.
15. Frisch, M. J.; Trucks, G. W.; Schlegel, H. B.; Scuseria, G. E.; Robb, M. A.; Cheeseman, J. R.; Scalmani, G.; Barone, V.; Petersson, G. A.; Nakatsuji, H.; Li, X.; Caricato, M.; Marenich, A. V.; Bloino, J.; Janesko, B. G.; Gomperts, R.; Mennucci, B.; Hratchian, H. P.; Ortiz, J. V.; Izmaylov, A. F.; Sonnenberg, J. L.; Williams-Young, D.; Ding, F.; Lipparini, F.; Egidi, F.; Goings, J.; Peng, B.; Petrone, A.; Henderson, T.; Ranasinghe, D.; Zakrzewski, V. G.; Gao, J.; Rega, N.; Zheng, G.; Liang, W.; Hada, M.; Ehara, M.; Toyota, K.; Fukuda, R.; Hasegawa, J.; Ishida, M.; Nakajima, T.; Honda, Y.; Kitao, O.; Nakai, H.; Vreven, T.; Throssell, K.; Montgomery, J. A., Jr.; Peralta, J. E.; Ogliaro, F.; Bearpark, M. J.; Heyd, J. J.; Brothers, E. N.; Kudin, K. N.; Staroverov, V. N.; Keith, T. A.; Kobayashi, R.; Normand, J.; Raghavachari, K.; Rendell, A. P.; Burant, J. C.; Iyengar, S. S.; Tomasi, J.; Cossi, M.; Millam, J. M.; Klene, M.; Adamo, C.; Cammi, R.; Ochterski, J. W.; Martin, R. L.; Morokuma, K.; Farkas, O.; Foresman, J. B.; Fox, D. J. Gaussian 16, Revision C.01, Gaussian, Inc., Wallingford CT, 2016.
16. J. J. P. Stewart, *J. Mol. Model.*, 2007, **13**, 1173-1213.
17. G. Rauhut, T. Clark and T. Steinke, *J. Am. Chem. Soc.*, 1993, **115**, 9174-9181.
18. Clark, T.; Alex, A.; Beck, B.; Burkhardt, F.; Chandrasekhar, J.; Gedeck, P.; Horn, A. H. C.; Hutter, M.; Martin, B.; Dral, P. O.; Rauhut, G.; Sauer, W.; Schindler, T.; Steinke, T. VAMP, FAU Erlangen-Nürnberg, Erlangen, 2019.
19. B. S. Basel, J. Zirzlmeier, C. Hetzer, S. R. Reddy, B. T. Phelan, M. D. Krzyaniak, P. B. Coto, M. K. Volland, R. M. Young, T. Clark, M. Thoss, R. R. Tykwinski, M. R. Wasielewski and D. M. Guldi, *Chem*, 2018, **4**, 1092–1111.

20. B. J. Walker, A. J. Musser, D. Beljonne and R. H. Friend, *Nat. Chem.*, 2013, **5**, 1019-1024.
21. J. Zirzlmeier, D. Lehnherr, P. B. Coto, E. T. Chernick, R. Casillas, B. S. Basel, M. Thoss, R. R. Tykwinski and D. M. Guldi, *Proc. Natl. Acad. Sci. U.S.A.*, 2015, **112**, 5325-5330.
22. B. S. Basel, J. Zirzlmeier, C. Hetzer, B. T. Phelan, M. D. Krzyaniak, S. R. Reddy, P. B. Coto, N. E. Horwitz, R. M. Young, F. J. White, F. Hampel, T. Clark, M. Thoss, R. R. Tykwinski, M. R. Wasielewski and D. M. Guldi, *Nat. Commun.*, 2017, **8**, 15171.
